# Supplementary material for: Isolation and Absolute Configurations of Diversiform C17, C21 and C25 Terpenoids from the Marine Sponge Cacospongia sp
Source: Mar Drugs. 2018 Dec 28;17(1):14. doi: 10.3390/md17010014 (PMC6356455; doi:10.3390/md17010014)
Supplement: Supplementary file 1 [file marinedrugs-17-00014-s001.pdf]

Supplementary Materials

Isolation and Absolute Configurations of Diversiform C17, C21 and C25 Terpenoids from the Marine Sponge *Cacospongia* sp.

Xingwang Zhang [1,2,†], Ping-Lin Li [1,3,†], Guo-Fei Qin [1,3], Shengying Li [2,4], Nicole J. de Voogd [5], Xu-Li Tang [6,*] and Guo-Qiang Li [1,3,*]

1 Key Laboratory of Marine Drugs, Chinese Ministry of Education, School of Medicine and Pharmacy, Ocean University of China, Qingdao 266003, China; zhangxw@qibebt.ac.cn (X.-W.Z.); ipinglin@ouc.edu.cn (P.-L.L.); qinguofei@126.com (G.-F.Q.)
2 Shandong Provincial Key Laboratory of Synthetic Biology, CAS Key Laboratory of Biofuels, Qingdao Institute of Bioenergy and Bioprocess Technology, Chinese Academy of Sciences, Qingdao, Shandong 266101, China
3 Laboratory of Marine Drugs and Biological Products, National Laboratory for Marine Science and Technology, Qingdao 266235, China
4 Laboratory for Marine Biology and Biotechnology, Qingdao National Laboratory for Marine Science and Technology, Qingdao, Shandong 266237, China
5 National Museum of Natural History, 2300 RA Leiden, The Netherlands; nicole.devoogd@naturalis.nl
6 College of Chemistry and Chemical Engineering, Ocean University of China, Qingdao 266100, China; tangxvli@ouc.ed.cn (X-L.T.)
† These authors contributed equally to this paper.
* Correspondence: tangxuli@ouc.edu.cn (X.-L.T.); liguoqiang@ouc.edu.cn (G.-Q.L.); Tel.: +86-532-82032323 (X.-L.T.); +86-532-82032323 (G.-Q.L.)

Received: 26 November 2018; Accepted: 20 December 2018; Published: date

Contents

I. Photos of sponge specimen

II. Computational details

Figure S1. Stable conformers of the key chiral structure in compounds **1a/1b** and **2a/2b** with 4*R*,5*R* (**p1-a**), 4*S*,5*S* (**p1-b**), 4*R*,5*S* (**p1-c**), and 4*S*,5*R* (**p1-d**) configurations.

Table S1. Important thermodynamic parameters (a.u.) of the optimized o key chiral structure in compounds **1a/1b** and **2a/2b** at B3LYP/6-31G(d,p) level in the gas phase.

Table S2. Optimized Z-Matrixes of the key chiral structure in compounds **1a/1b** and **2a/2b** in the Gas Phase (Å) at B3LYP/6-31G(d,p) level.

Figure S2. Stable conformers of the key chiral structure in compound **6** with 3*R*,4*R* (**6a**) and 3*S*,4*S* (**6b**) configurations, respectively

Table S3. Important thermodynamic parameters (a.u.) of the optimized o key chiral structure in compound **6** at B3LYP/6-31G(d,p) level in the gas phase.

Table S4. Optimized Z-Matrixes of the key chiral structure in compound **6** in the Gas Phase (Å) at B3LYP/6-31G(d,p) level.

III. Chiral HPLC separation profiles of 1a/1b 5a/5b

Figure S3. Chiral HPLC separation of compounds **1a** and **1b**.

Figure S4. Chiral HPLC separation of compounds **2a** and **2b**.

Figure S5. Chiral HPLC analysis of compounds **3**.

Figure S6. Chiral HPLC separation of the LAH reduction products of compounds **3** (**3a-r**/**3b-r**).

Figure S7. Chiral HPLC separation of compounds **4a/4b** and **5a/5b**.

Scheme S1. NaOH hydrolysis reaction of **3** and CH_2N_2 diazotization of the hydrolyzates.

IV. HR-MS, 1D and 2D NMR spectra of the new compounds

Figure S8. HR-ESI-MS of compounds **1a/1b**.

Figure S9. ^{1}H NMR spectrum (500 MHz, $CDCl_3$) of compounds **1a/1b**.

Figure S10. ^{13}C NMR spectrum (125 MHz, $CDCl_3$) of compounds **1a/1b**.

Figure S11. DEPT spectrum (125 MHz, $CDCl_3$) of compounds **1a/1b**.

Figure S12. HR-ESI-MS of compounds **2a/2b**.

Figure S13. ^{1}H NMR spectrum (500 MHz, $CDCl_3$) of compounds **2a/2b**.

Figure S14. ^{13}C NMR spectrum (125 MHz, $CDCl_3$) of compounds **2a/2b**.

Figure S15. DEPT spectrum (125 MHz, $CDCl_3$) of compounds **2a/2b**.

Figure S16. HMQC spectrum of compounds **2a/2b**.

Figure S17. ^{1}H-^{1}H COSY spectrum of compounds **2a/2b**.

Figure S18. HMBC spectrum of compounds **2a/2b**.

Figure S19. NOESY spectrum of compounds **2a/2b**.

Figure S20. HR-ESI-MS of compounds **3a/3b**.

Figure S21. ^{1}H NMR spectrum (500 MHz, $CDCl_3$) of compounds **3a/3b**.

Figure S22. ^{13}C NMR spectrum (125 MHz, $CDCl_3$) of compounds **3a/3b**.

Figure S23. HSQC spectrum of compounds **3a/3b**.

Figure S24. ^{1}H-^{1}H COSY spectrum of compounds **3a/3b**.

Figure S25. HMBC spectrum of compounds **3a/3b**.

Figure S26. NOESY spectrum of compounds **3a/3b**.

Figure S27. HR-ESI-MS of compounds **4a/4b**.

Figure S28. ^{1}H NMR spectrum (500 MHz, $CDCl_3$) of compounds **4a/4b**.

Figure S29. ^{13}C NMR spectrum (125 MHz, $CDCl_3$) of compounds **4a/4b**.

Figure S30. DEPT spectrum (125 MHz, $CDCl_3$) of compounds **4a/4b**.

Figure S31. HR-ESI-MS of compounds **5a/5b**.

Figure S32. ^{1}H NMR spectrum (500 MHz, $CDCl_3$) of compounds **5a/5b**.
Figure S33. ^{13}C NMR spectrum (125 MHz, $CDCl_3$) of compounds **5a/5b**.
Figure S34. DEPT spectrum (125 MHz, $CDCl_3$) of compounds **5a/5b**.
Figure S35. HR-ESI-MS of compound **6**.
Figure S36. ^{1}H NMR spectrum (500 MHz, $CDCl_3$) of compound **6**.
Figure S37. ^{13}C NMR spectrum (125 MHz, $CDCl_3$) of compound **6**.
Figure S38. DEPT spectrum (125 MHz, $CDCl_3$) of compound **6**.
Figure S39. HSQC spectrum of compound **6**.
Figure S40. ^{1}H-^{1}H COSY spectrum of compound **6**.
Figure S41. HMBC spectrum of compound **6**.
Figure S42. NOESY spectrum of compound **6**.
Figure S43. HR-ESI-MS of compound **7.**
Figure S44. ^{1}H NMR spectrum (500 MHz, DMSO-d_6) of compound **7**.
Figure S45. ^{13}C NMR spectrum (125 MHz, DMSO-d_6) of compound **7**.
Figure S46. DEPT spectrum (125 MHz, DMSO-d_6) of compound **7**.
Figure S47. HSQC spectrum of compound **7**.
Figure S48. ^{1}H-^{1}H COSY spectrum of compound **7**.
Figure S49. HMBC spectrum of compound **7**.

I. Photos of sponge specimen

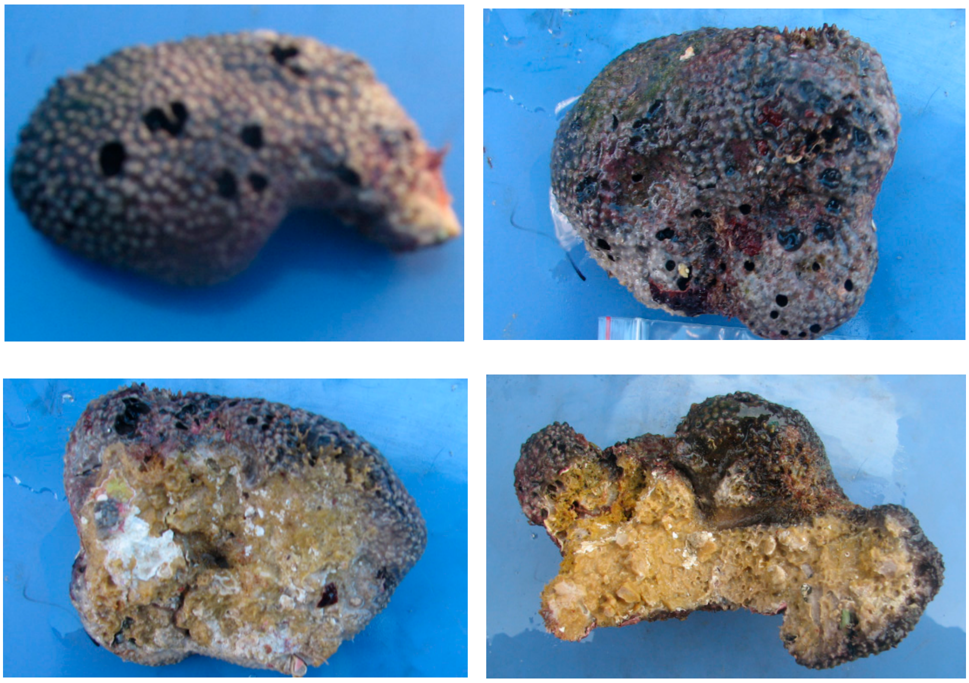

***Cacospongia* sp.**

The sponge *Cacospongia* sp. was in irregular shaped, and with a detailed feather of tight encrusting, rough, black-brownish crust with irregularity distributed holes on the surface, thick and solid, had the dimensions of around 4 × 5 cm ~ 6 × 8 cm.

II. Computational details

The quantum chemical calculations were performed using the density functional theory (DFT) as carried out in the Gaussian 09 [1]. The initial structures of the key chiral structures in compounds **1**, **2**, and **6** were built with Spartan 10 software, and all trial structures were first minimized based on molecular mechanics calculations. Conformational search was performed by Spartan 10 software using MMFF force filed, and conformers occurring within a 10 kcal/mol energy window from the global minimum were chosen for geometry optimization in the gas phase with the DFT method at the B3LYP/DGDZVP level. The B3LYP/DGDZVP harmonic vibrational frequencies were further calculated to confirm their stability. The spin-allowed excitation energies and rotatory (*R*n) and oscillator strengths (*f*n) of the lowest excited states of stable conformers were calculated for ECD spectra using TD-DFT method with the basis set RB3LYP/DGDZVP. Solvent effects of methanol solution were evaluated at the same DFT level by using the SCRF/PCM method in agreement with the experiment condition. Electronic transitions were expanded as Gaussian curves with a FQHM (full width at half maximum) for each peak of 0.32 eV. The ECD spectra were combined after Boltzmann weighting according to their population contribution.

Reference

1. Frisch, M.J.; Trucks, G.W.; Schlegel, H.B.; Scuseria, G.E.; Robb, M.A.; Cheeseman, J.R.; Scalmani, G.; Barone, V.; Mennucci, B.; Petersson, G.A.; et al. *Gaussian 09*, Revision A.1; Gaussian, Inc.: Wallingford, CT, USA, 2009.

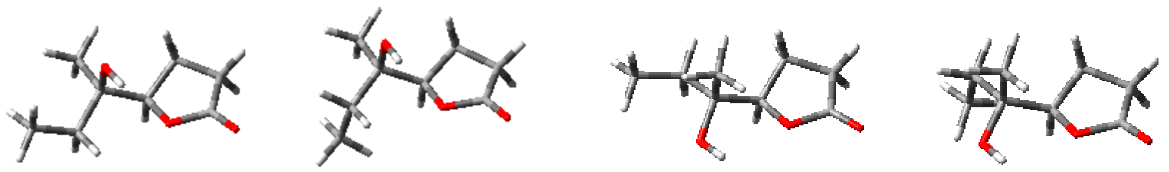

p1-a1 (52.5%) **p1-a2 (24.9%)** **p1-a3 (6.5%)** **p1-a4 (5.9%)**

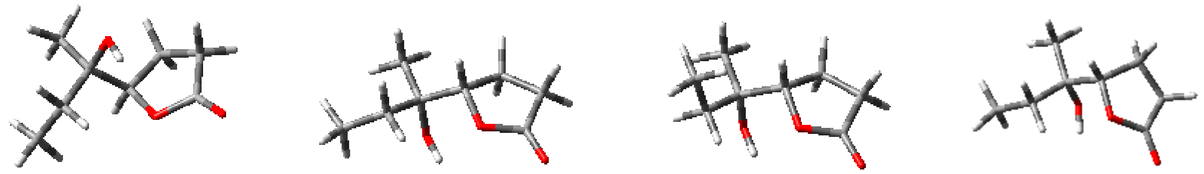

p1-a5 (5.3%) **p1-b1 (57.0%)** **p1-b2 (27.1%)** **p1-b3 (6.5%)**

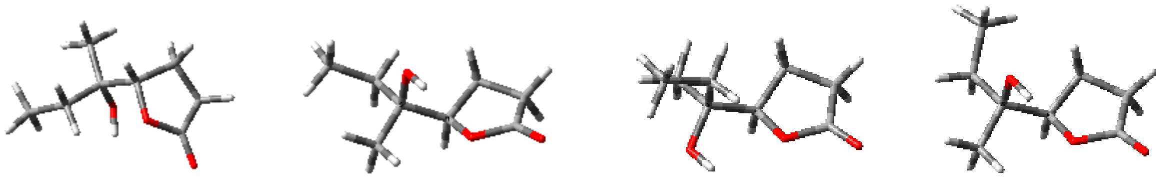

p1-b4 (5.7%) **p1-c1 (72.9%)** **p1-c2 (13.8%)** **p1-c3 (2.8%)**

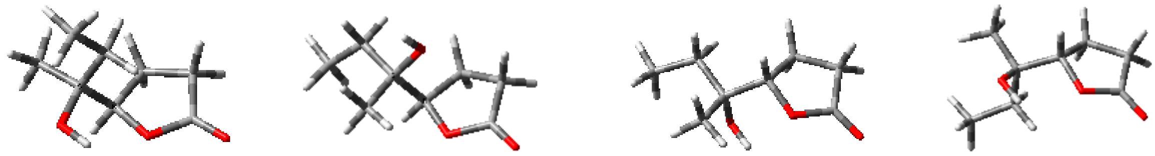

p1-c4 (2.8%) **p1-c5 (1.4%)** **p1-d1 (71.7%)** **p1-d2 (13.5%)**

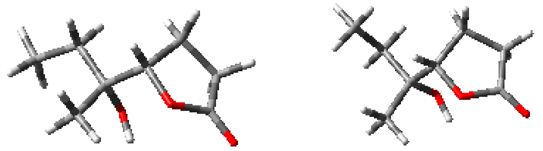

p1-d3 (8.7%) **p1-d4 (2.38%)**

Figure S1. Stable conformers of the key chiral structure in compounds **1a/1b** and **2a/2b** with 4*R*,5*R* (**p1-a**), 4*S*,5*S* (**p1-b**), 4*R*,5*S* (**p1-c**), and 4*S*,5*R* (**p1-d**) configurations.

Table S1. Important thermodynamic parameters (a.u.) of the optimized o key chiral structure in compounds **1a/1b** and **2a/2b** at B3LYP/6-31G(d,p) level in the gas phase.

Conformations	E+ZPE	G	Conformations	E+ZPE	G
**p1-a1**	–538.775574	–538.812577	**p1-b1**	–538.775574	–538.812577
**p1-a2**	–538.774784	–538.811876	**p1-b2**	–538.774784	–538.811876
**p1-a3**	–538.773475	–538.810603	**p1-b3**	–538.772980	–538.810536
**p1-a4**	–538.773266	–538.810519	**p1-b4**	–538.772705	–538.810404
**p1-a5**	–538.772705	–538.810404	**p1-d1**	–538.775222	–538.812165
**p1-c1**	–538.775223	–538.812166	**p1-d2**	–538.773373	–538.810593
**p1-c2**	–538.773373	–538.810594	**p1-d3**	–538.772821	–538.810171
**p1-c3**	–538.772135	–538.809101	**p1-d4**	–538.772003	–538.808919
**p1-c4**	–538.772033	–538.809104			
**p1-c5**	–538.771025	–538.808496			

Table S2. Optimized Z-Matrixes of the key chiral structure in compounds **1a/1b** and **2a/2b** in the Gas Phase (Å) at B3LYP/6-31G(d,p) level.

**p1-a1**				**p1-a2**				**p1-a3**			
C	3.039	−0.5952	0.0608	C	2.9196	−0.5972	−0.2572	C	2.668	−0.9015	−1.0558
C	1.9738	0.4403	0.3252	C	1.9236	0.4716	0.1215	C	1.4076	−0.0781	−1.1745
C	0.7704	−0.1377	−0.397	C	0.6038	−0.1479	−0.3022	C	0.4403	−0.8481	−0.3007
O	0.9166	−1.5588	−0.2199	O	0.7841	−1.5514	−0.0442	O	1.2573	−1.3676	0.764
C	2.231	−1.8546	0.0037	C	2.1173	−1.8463	−0.0619	C	2.5518	−1.4472	0.3352
C	−0.6114	0.3233	0.1374	C	−0.6424	0.37	0.4567	C	−0.753	−0.0488	0.2833
C	−1.7449	−0.4126	−0.6172	C	−1.9256	−0.4205	0.109	C	−1.6416	0.4935	−0.8566
C	−3.1549	−0.0684	−0.1495	C	−2.3428	−0.387	−1.3538	C	−2.9111	1.2002	−0.393
O	−0.7047	−0.0128	1.5286	O	−0.4296	0.1935	1.8691	O	−1.5425	−0.9746	1.0498
C	−0.751	1.845	0.025	C	−0.837	1.8724	0.2306	C	−0.3112	1.0614	1.2464
O	2.6698	−2.9873	0.1075	O	2.5696	−2.9744	0.037	O	3.4637	−1.921	0.9912
H	3.5309	−0.4378	−0.9033	H	3.7934	−0.5826	0.3984	H	3.5623	−0.2841	−1.1646
H	3.7726	−0.632	0.8688	H	3.2189	−0.5236	−1.3064	H	2.6808	−1.7319	−1.7675
H	2.252	1.4354	−0.0328	H	2.1228	1.4302	−0.3659	H	1.5986	0.9254	−0.779
H	1.7986	0.5052	1.4059	H	1.9643	0.6299	1.2055	H	1.0635	0.0259	−2.2073
H	0.8363	0.0664	−1.474	H	0.4662	−0.0263	−1.3839	H	0.0519	−1.7241	−0.8385
H	−1.6248	−1.4956	−0.4877	H	−1.8106	−1.4674	0.4183	H	−1.9512	−0.3443	−1.4949
H	−1.6706	−0.2085	−1.6923	H	−2.7544	−0.0396	0.721	H	−1.0714	1.1855	−1.4878
H	−3.4116	0.9694	−0.3799	H	−2.5082	0.6352	−1.7049	H	−2.6792	2.1216	0.1492
H	−3.8816	−0.706	−0.6635	H	−1.5948	−0.8606	−1.9956	H	−3.5166	0.5577	0.2534
H	−3.2719	−0.2272	0.9265	H	−3.2812	−0.9364	−1.4835	H	−3.5218	1.4743	−1.2596
H	−0.6063	−0.9817	1.5936	H	−0.2911	−0.7607	2.0184	H	−0.9376	−1.4019	1.6866
H	−1.6812	2.198	0.4822	H	−0.008	2.4482	0.6569	H	0.1835	1.8853	0.724
H	−0.7252	2.1739	−1.019	H	−0.9158	2.1199	−0.8321	H	0.3734	0.6764	2.0101
H	0.0491	2.3584	0.5691	H	−1.7422	2.227	0.737	H	−1.1656	1.4671	1.7995
H	−0.7252	2.1739	−1.019	C	2.9196	−0.5972	−0.2572	C	2.668	−0.9015	−1.0558
H	0.0491	2.3584	0.5691	C	1.9236	0.4716	0.1215	C	1.4076	−0.0781	−1.1745
**p1-a4**				**p1-a5**				**p1-b1**			
C	2.3185	−1.2024	−1.2472	C	2.6886	−0.5104	0.4861	C	−3.0366	−0.6101	0.055
C	1.1523	−0.243	−1.2643	C	1.9784	0.5337	−0.3389	C	−1.9766	0.4286	0.3281
C	0.304	−0.7549	−0.1192	C	0.607	−0.066	−0.6036	C	−0.77	−0.1369	−0.3991
O	1.2603	−1.223	0.8501	O	0.8439	−1.4849	−0.6014	O	−0.9096	−1.5602	−0.2357
C	2.4302	−1.5246	0.2123	C	1.9929	−1.7692	0.0772	C	−2.2224	−1.8645	−0.0159
C	−0.6614	0.2651	0.5328	C	−0.512	0.2826	0.4199	C	0.6098	0.3242	0.1409
C	−1.6517	0.8888	−0.4729	C	−1.8035	−0.5433	0.1929	C	1.7468	−0.3989	−0.6213
C	−2.5061	−0.1222	−1.2301	C	−2.4659	−0.3643	−1.1653	C	3.1551	−0.0511	−0.1507
O	−1.4336	−0.4423	1.5192	O	−0.0519	−0.0169	1.7489	O	0.705	−0.0256	1.5289
C	0.0744	1.385	1.2838	C	−0.8207	1.785	0.4042	C	0.7433	1.8474	0.0461
O	3.4044	−2.0112	0.7608	O	2.368	−2.9012	0.3339	O	−2.6555	−3.0006	0.075
H	3.2311	−0.7319	−1.6195	H	3.7526	−0.5556	0.2418	H	−3.5294	−0.447	−0.9076
H	2.1007	−2.1197	−1.8019	H	2.551	−0.35	1.5583	H	−3.7693	−0.6586	0.8635
H	1.5211	0.7706	−1.073	H	2.5088	0.6426	−1.2943	H	−2.2605	1.4251	−0.0216
H	0.6234	−0.2342	−2.2214	H	1.9697	1.5188	0.1324	H	−1.8013	0.486	1.409

H	−0.2623	−1.644	−0.4264	H	0.2827	0.2181	−1.6112	H	−0.8363	0.078	−1.4741
H	−1.1157	1.5139	−1.1972	H	−1.5928	−1.6102	0.3437	H	1.6325	−1.4838	−0.5017
H	−2.3407	1.5503	0.0689	H	−2.5303	−0.2904	0.9765	H	1.6701	−0.1858	−1.6945
H	−3.0639	−0.7681	−0.5451	H	−2.7503	0.6766	−1.3419	H	3.4067	0.99	−0.3728
H	−1.897	−0.7529	−1.884	H	−3.3784	−0.9674	−1.2156	H	3.8857	−0.6823	−0.6681
H	−3.2315	0.4017	−1.8615	H	−1.812	−0.6914	−1.9782	H	3.2717	−0.2176	0.9247
H	−0.7972	−0.8892	2.1094	H	−0.1071	−0.9823	1.8751	H	0.6109	−0.9955	1.5829
H	0.668	2.012	0.6127	H	−1.0673	2.1429	−0.6001	H	1.6716	2.1988	0.5082
H	0.7349	0.9814	2.0587	H	−1.6626	2.0134	1.0684	H	0.7183	2.1885	−0.9939
H	−0.6402	2.0307	1.8079	H	0.0222	2.3729	0.7832	H	−0.0599	2.3519	0.5946
**p1-b2**				**p1-b3**				**p1-b4**			
C	−2.9171	−0.6077	−0.2629	C	−2.6035	−0.6085	0.7985	C	−2.6867	−0.5271	0.4842
C	−1.9257	0.4615	0.1268	C	−2.1181	0.5319	−0.0618	C	−1.9805	0.5289	−0.3292
C	−0.6032	−0.1477	−0.3035	C	−0.8335	0.0098	−0.6845	C	−0.6072	−0.0629	−0.6012
O	−0.7774	−1.5546	−0.0605	O	−1.0551	−1.4098	−0.7795	O	−0.8389	−1.4826	−0.6148
C	−2.1092	−1.8553	−0.0813	C	−2.0088	−1.798	0.1154	C	−1.9867	−1.7787	0.0605
C	0.6407	0.3674	0.461	C	0.489	0.2881	0.0942	C	0.5112	0.2783	0.4253
C	1.9275	−0.4136	0.1049	C	1.6681	−0.458	−0.5846	C	1.8052	−0.5404	0.1885
C	2.3443	−0.3629	−1.3574	C	3.0196	−0.2813	0.1005	C	2.4648	−0.3463	−1.1691
O	0.4288	0.1752	1.8713	O	0.3599	−0.1846	1.4413	O	0.0531	−0.0372	1.7513
C	0.8289	1.8729	0.2509	C	0.7606	1.796	0.1712	C	0.8148	1.7818	0.4256
O	−2.5566	−2.9862	0.0052	O	−2.2994	−2.9619	0.3373	O	−2.358	−2.9151	0.3043
H	−3.7906	−0.6039	0.3931	H	−3.6944	−0.6669	0.8038	H	−3.7507	−0.5734	0.2396
H	−3.2171	−0.5241	−1.3112	H	−2.22	−0.5452	1.8199	H	−2.5494	−0.3786	1.5581
H	−2.1291	1.4243	−0.3505	H	−2.8526	0.7036	−0.8598	H	−2.5116	0.6469	−1.2829
H	−1.9669	0.6084	1.2124	H	−2.0236	1.4704	0.4875	H	−1.9752	1.5085	0.1535
H	−0.4661	−0.0139	−1.3838	H	−0.7363	0.4003	−1.7047	H	−0.2846	0.2337	−1.6056
H	1.8171	−1.4643	0.4031	H	1.7572	−0.141	−1.631	H	1.5993	−1.6095	0.3297
H	2.7545	−0.0356	0.7207	H	1.4608	−1.5356	−0.5944	H	2.5328	−0.2917	0.973
H	2.5043	0.6637	−1.698	H	2.9738	−0.5603	1.1574	H	2.7424	0.6977	−1.3381
H	1.5988	−0.8338	−2.0042	H	3.3758	0.7501	0.0253	H	1.8114	−0.6708	−1.9839
H	3.2856	−0.9061	−1.4929	H	3.7671	−0.9192	−0.3832	H	3.3808	−0.9435	−1.2259
H	0.2942	−0.7817	2.0103	H	0.4576	−1.1548	1.4266	H	0.1119	−1.004	1.8671
H	−0.0026	2.4406	0.6834	H	−0.0523	2.3296	0.6737	H	−0.0293	2.3626	0.8123
H	0.9064	2.1323	−0.8091	H	0.8961	2.2301	−0.8247	H	1.0584	2.1513	−0.575
H	1.7326	2.226	0.7612	H	1.6513	2.0132	0.7706	H	1.657	2.0058	1.0908
**p1-c1**				**p1-c2**				**p1-c3**			
C	1.2183	−1.5555	−2.2604	C	−0.7771	−1.788	−2.3306	C	1.3409	−1.1669	−2.237
C	1.3796	−0.9107	−0.9064	C	−1.1772	−1.3691	−0.9356	C	1.3905	−0.4352	−0.9175
C	0.2917	0.1489	−0.9103	C	0.1398	−0.8979	−0.3556	C	0.0496	0.2693	−0.8694
O	−0.7637	−0.4323	−1.6961	O	0.8339	−0.3101	−1.4698	O	−0.837	−0.6465	−1.5464
C	−0.2502	−1.4014	−2.5059	C	0.3463	−0.8419	−2.6297	C	−0.1181	−1.4641	−2.3682
C	−0.2248	0.571	0.4949	C	0.0606	0.1094	0.8206	C	−0.524	0.5823	0.5385
C	0.9112	1.2576	1.2884	C	−0.5091	1.4851	0.3991	C	0.3338	1.5787	1.3526
C	0.5199	1.7546	2.6767	C	−0.5219	2.5321	1.5101	C	1.5867	1.0187	2.0162
O	−0.6204	−0.6086	1.2091	O	1.4065	0.3187	1.2823	O	−0.6541	−0.6366	1.2806

C	−1.4536	1.4808	0.3488	C	−0.7178	−0.5016	1.9896	C	−1.9422	1.1692	0.394
O	−0.8951	−2.0111	−3.3433	O	0.7909	−0.5941	−3.7377	O	−0.6129	−2.288	−3.1182
H	1.5067	−2.6089	−2.2413	H	−1.5992	−1.6628	−3.0388	H	1.9317	−2.0848	−2.209
H	1.7698	−1.016	−3.0356	H	−0.3966	−2.813	−2.355	H	1.6522	−0.5291	−3.0692
H	1.207	−1.6642	−0.1293	H	−1.9061	−0.5547	−1.0022	H	1.488	−1.1639	−0.1043
H	2.3793	−0.4944	−0.7523	H	−1.6315	−2.1823	−0.3617	H	2.2367	0.2551	−0.8668
H	0.6331	1.0406	−1.453	H	0.7462	−1.7597	−0.0431	H	0.0813	1.1903	−1.4664
H	1.7363	0.5476	1.4265	H	−1.5301	1.3777	0.0175	H	−0.2802	1.978	2.1715
H	1.3066	2.1045	0.7144	H	0.0977	1.8969	−0.4167	H	0.6028	2.4377	0.7255
H	0.0925	0.9533	3.2866	H	0.4766	2.6836	1.9307	H	2.3429	0.7171	1.2904
H	1.4051	2.1348	3.1974	H	−1.2024	2.2507	2.3191	H	1.3551	0.1627	2.6574
H	−0.2022	2.5744	2.6196	H	−0.8657	3.4926	1.1119	H	2.0407	1.7887	2.6495
H	−1.2294	−0.3497	1.9218	H	1.9271	0.6132	0.5105	H	−1.2311	−1.2264	0.7588
H	−2.2594	0.9741	−0.1936	H	−0.3356	−1.5015	2.2273	H	−2.6178	0.4732	−0.1152
H	−1.8806	1.7422	1.3226	H	−1.7862	−0.5854	1.7671	H	−1.9305	2.1124	−0.162
H	−1.2046	2.4054	−0.1811	H	−0.5959	0.088	2.9049	H	−2.3898	1.3511	1.3784
**p1-c4**				**p1-c5**				**p1-d1**			
C	−1.1056	−1.0431	−2.2284	C	1.0744	−1.6642	−1.7586	C	−2.8433	−1.2034	−0.2404
C	−1.0342	−1.6809	−0.8629	C	1.607	−0.3919	−1.1439	C	−1.4406	−0.9905	−0.7529
C	0.2323	−1.1241	−0.2419	C	0.3615	0.4269	−0.8555	C	−0.7261	−0.4205	0.4596
O	1.0482	−0.7033	−1.3513	O	−0.5581	0.0246	−1.8879	O	−1.74	0.3397	1.1408
C	0.3136	−0.6588	−2.4977	C	−0.2214	−1.2121	−2.3556	C	−2.9735	−0.1102	0.7738
C	0.0744	0.0458	0.7716	C	−0.2775	0.2236	0.5502	C	0.5142	0.462	0.1416
C	−0.5959	1.2958	0.1601	C	0.6281	0.7109	1.7043	C	1.6127	−0.396	−0.5276
C	−0.6595	2.5037	1.0903	C	1.0181	2.1807	1.661	C	2.9003	0.3493	−0.8627
O	1.3981	0.4169	1.1939	O	−0.4821	−1.1834	0.7656	O	0.1203	1.486	−0.7826
C	−0.662	−0.4343	2.0278	C	−1.6563	0.8992	0.6094	C	1.0179	1.1384	1.4258
O	0.7553	−0.3058	−3.5784	O	−0.889	−1.8444	−3.1587	O	−4.0224	0.2915	1.2505
H	−1.7265	−0.1439	−2.2277	H	0.8844	−2.438	−1.0112	H	−3.5836	−1.0938	−1.036
H	−1.4658	−1.75	−2.98	H	1.7471	−2.0414	−2.5328	H	−2.9514	−2.17	0.2596
H	−1.9403	−1.5252	−0.272	H	2.2525	−0.5686	−0.2807	H	−1.4675	−0.2723	−1.5807
H	−0.9217	−2.7656	−0.9869	H	2.2115	0.1332	−1.8947	H	−0.982	−1.9114	−1.1243
H	0.785	−1.9341	0.251	H	0.5701	1.4902	−1.0152	H	−0.4301	−1.2298	1.1404
H	−1.6155	1.0575	−0.1624	H	1.5394	0.1017	1.7457	H	1.2288	−0.8035	−1.4709
H	−0.037	1.6132	−0.7283	H	0.1236	0.5125	2.66	H	1.8617	−1.2493	0.1153
H	0.3353	2.7907	1.4439	H	0.1397	2.8317	1.6686	H	2.7075	1.2273	−1.4857
H	−1.2964	2.309	1.9576	H	1.619	2.4128	0.7775	H	3.5778	−0.3092	−1.4162
H	−1.0837	3.3611	0.5573	H	1.6209	2.4302	2.5405	H	3.4252	0.6705	0.0415
H	1.9266	0.5567	0.3842	H	−1.0492	−1.2915	1.5498	H	0.7633	2.2137	−0.7306
H	−0.2165	−1.3615	2.4067	H	−2.3688	0.4032	−0.0595	H	0.2369	1.7585	1.8793
H	−1.7239	−0.6161	1.838	H	−1.6038	1.9572	0.3349	H	1.8485	1.8226	1.2225
H	−0.5742	0.2903	2.8445	H	−2.0842	0.8247	1.6157	H	1.3477	0.3992	2.1631
**p1-d2**				**p1-d3**				**p1-d4**			
C	−2.6745	−1.3554	0.8776	C	−2.6051	−0.6154	−0.7152	C	−2.5557	−0.6515	−0.6938
C	−1.1648	−1.3729	0.9283	C	−1.3775	−1.4421	−0.4234	C	−1.2044	−1.3069	−0.5536
C	−0.8302	0.1036	0.9075	C	−0.6559	−0.6684	0.6674	C	−0.5109	−0.5048	0.5336

O	–1.8437	0.6893	0.0717	O	–1.7232	–0.015	1.3803	O	–1.5939	–0.0483	1.3668
C	–2.9454	–0.1183	0.0756	C	–2.8242	0.095	0.582	C	–2.765	–0.0637	0.6655
C	0.5805	0.4911	0.3933	C	0.3942	0.3869	0.204	C	0.3379	0.7125	0.0678
C	0.7864	0.1683	–1.1056	C	1.5643	–0.3006	–0.5373	C	1.4794	0.3305	–0.9001
C	2.1212	0.6401	–1.6774	C	2.6659	0.6434	–1.0111	C	2.4648	–0.7064	–0.3811
O	0.7072	1.9145	0.5559	O	–0.2224	1.3069	–0.7065	O	–0.502	1.6293	–0.6553
C	1.6584	–0.1516	1.2713	C	0.9005	1.1904	1.4141	C	0.8943	1.472	1.284
O	–3.9981	0.1554	–0.4749	O	–3.827	0.7182	0.889	O	–3.8188	0.3771	1.0931
H	–3.1141	–1.2399	1.8724	H	–3.4615	–1.2465	–0.9639	H	–3.3326	–1.3838	–0.9257
H	–3.0699	–2.2439	0.38	H	–2.4288	0.1179	–1.5061	H	–2.55	0.148	–1.4388
H	–0.775	–1.8888	1.8107	H	–1.6968	–2.4107	–0.0167	H	–1.3504	–2.3344	–0.1946
H	–0.7844	–1.8881	0.0401	H	–0.7863	–1.6545	–1.3158	H	–0.6607	–1.377	–1.4977
H	–0.9743	0.5392	1.9061	H	–0.1756	–1.3707	1.3596	H	0.1093	–1.172	1.1429
H	–0.0003	0.6546	–1.6957	H	1.1876	–0.8049	–1.4339	H	2.0364	1.2388	–1.168
H	0.7	–0.9094	–1.2813	H	2.0115	–1.0703	0.1036	H	1.0606	–0.02	–1.8514
H	2.1595	0.4315	–2.7518	H	3.2013	1.0924	–0.1695	H	1.9767	–1.6664	–0.1904
H	2.2575	1.7171	–1.542	H	2.2632	1.4444	–1.6378	H	2.9544	–0.3745	0.539
H	2.9641	0.1206	–1.2127	H	3.3994	0.0893	–1.6069	H	3.2469	–0.8801	–1.1278
H	–0.0432	2.3205	0.0804	H	–0.7692	1.9222	–0.1836	H	–1.0729	2.0834	–0.0079
H	2.6471	0.2744	1.0696	H	1.45	0.5542	2.1151	H	1.5109	0.8301	1.9198
H	1.7082	–1.2355	1.1261	H	0.0729	1.6619	1.9559	H	0.0878	1.8854	1.9
H	1.4609	0.0456	2.3317	H	1.5479	2.0172	1.103	H	1.4978	2.3288	0.9635

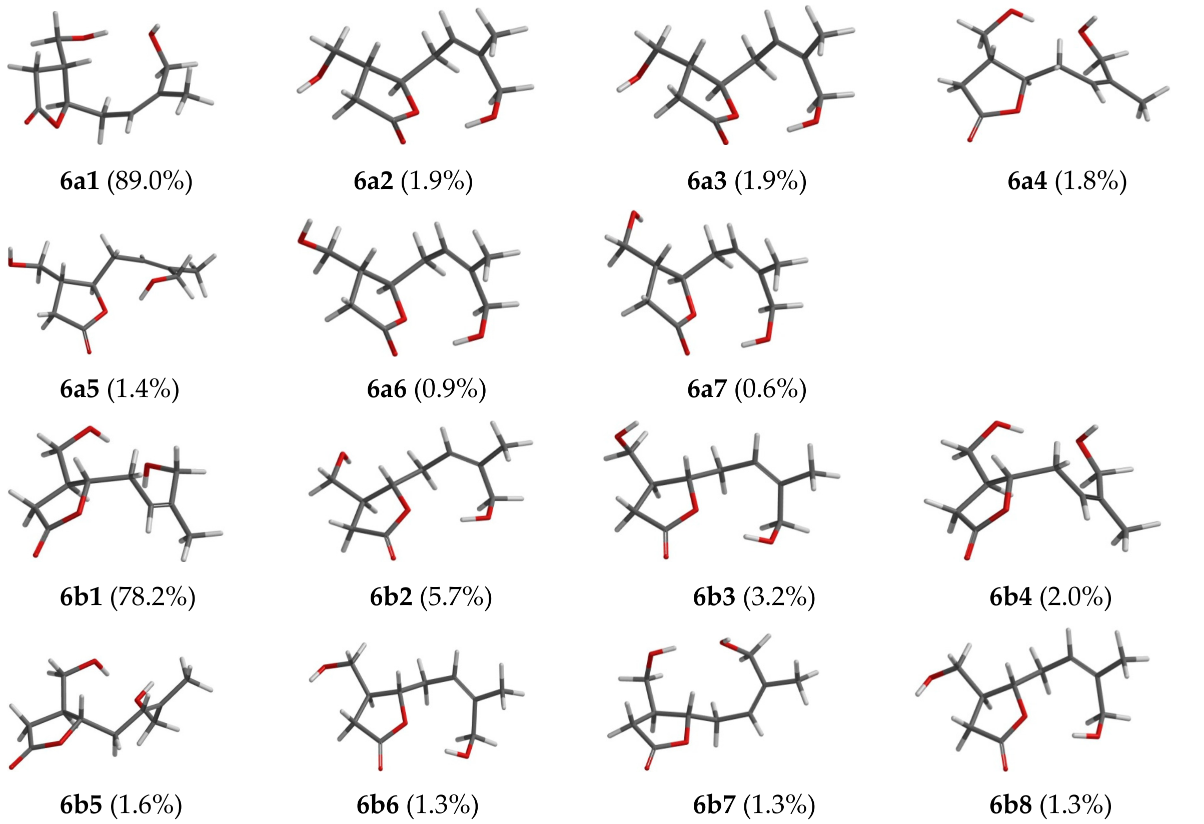

Figure S2. Stable conformers of the key chiral structure in compound **6** with 3*R*,4*R* (**6a**) and 3*S*,4*S* (**6b**) configurations, respectively.

Table S3. Important thermodynamic parameters (a.u.) of the optimized o key chiral structure in compound **6** at B3LYP/6-31G(d,p) level in the gas phase.

Conformations	E+ZPE	G	Conformations	E+ZPE	G
**6a1**	−691.345018	−691.386499	**6b1**	−691.345018	−691.386499
**6a2**	−691.340558	−691.382891	**6b2**	−691.341496	−691.384024
**6a3**	−691.340555	−691.382887	**6b3**	−691.340878	−691.383480
**6a4**	−691.340675	−691.382808	**6b4**	−691.341527	−691.383035
**6a5**	−691.340039	−691.382567	**6b5**	−691.340571	−691.382820
**6a6**	−691.339726	−691.382111	**6b6**	−691.339730	−691.382657
**6a7**	−691.339544	−691.381846	**6b7**	−691.342095	−691.383581
			**6b8**	−691.382656	−691.382656

Table S4. Optimized Z-Matrixes of the key chiral structure in compound **6** in the Gas Phase (Å) at B3LYP/6-31G(d,p) level.

**6a1**				**6a2**				**6a3**			
C	1.021268	0.860014	−0.370334	C	1.726955	−0.326185	0.573242	C	1.726980	−0.325757	0.573247
C	2.395320	0.513100	−0.954254	C	1.770536	1.183251	0.851220	C	1.770275	1.183755	0.851060
C	2.736706	−0.823711	−0.307541	C	0.531616	1.702328	0.14404	C	0.531114	1.702334	0.143931
O	1.943340	−1.002672	0.788104	O	0.185971	0.831347	−0.835339	O	0.185847	0.831254	−0.835415
C	1.064114	0.136807	0.991840	C	0.950670	−0.403060	−0.763941	C	0.950781	−0.402860	−0.763990
O	3.558614	−1.633600	−0.648525	O	−0.107300	2.704971	0.352667	O	−0.108255	2.704691	0.352623
C	0.718684	2.357888	−0.271990	C	3.088588	−1.030104	0.519510	C	3.088629	−1.029591	0.519894
O	−0.503516	2.638670	0.382816	O	3.959235	−0.498222	−0.471791	O	3.959299	−0.498421	−0.471755
C	−2.178895	−1.142594	0.047652	C	−2.261218	−0.978097	0.195910	C	−2.260895	−0.978298	0.195910
C	−2.771730	−2.208181	−0.844945	C	−3.197398	−1.103610	1.370746	C	−3.196986	−1.103681	1.370827
C	−0.996775	−1.320104	0.659433	C	−1.091604	−1.638107	0.159673	C	−1.091284	−1.638329	0.159650
C	−0.272809	−0.357109	1.560438	C	−0.033550	−1.573423	−0.919435	C	−0.033102	−1.573553	−0.919363
H	1.540322	0.784710	1.740806	H	1.657126	−0.391163	−1.601168	H	1.657278	−0.390909	−1.601193
C	−2.992657	0.129838	0.173236	C	−2.758953	−0.044257	−0.894083	C	−2.758680	−0.044554	−0.894125
O	−2.619931	1.141990	−0.787555	O	−2.958823	1.281996	−0.418182	O	−2.958926	1.281650	−0.418207
H	0.244063	0.384098	−0.981391	H	1.129472	−0.807900	1.356680	H	1.129363	−0.807309	1.356687
H	2.436345	0.430912	−2.041897	H	1.766023	1.466679	1.904810	H	1.765802	1.467244	1.904631
H	3.159954	1.240866	−0.648830	H	2.638948	1.652504	0.370209	H	2.638492	1.653221	0.369916
H	1.505228	2.851138	0.313535	H	2.941043	−2.083685	0.257279	H	2.941149	−2.083303	0.258185
H	0.751010	2.799127	−1.282067	H	3.554375	−1.011031	1.514998	H	3.554438	−1.010024	1.515377
H	−1.226742	2.246242	−0.141516	H	4.403369	0.276827	−0.106808	H	4.402175	0.277852	−0.107826
H	−3.732113	−2.565811	−0.451579	H	−4.151644	−1.551349	1.062852	H	−4.151147	−1.551671	1.063044
H	−2.977057	−1.825304	−1.854608	H	−3.432923	−0.109117	1.763482	H	−3.432683	−0.109136	1.763324
H	−2.105284	−3.068075	−0.947851	H	−2.775578	−1.714538	2.173601	H	−2.775011	−1.714362	2.173782
H	−0.472509	−2.259426	0.487134	H	−0.855140	−2.278991	1.009452	H	−0.854845	−2.279291	1.009378
H	−0.058934	−0.852162	2.516735	H	0.553432	−2.500481	−0.921266	H	0.554197	−2.500397	−0.920849
H	−0.859826	0.538750	1.772892	H	−0.478571	−1.497766	−1.916311	H	−0.478011	−1.498291	−1.916322
H	−4.062572	−0.094561	0.064942	H	−3.748336	−0.387244	−1.224280	H	−3.747947	−0.387773	−1.224441
H	−2.855907	0.607165	1.144530	H	−2.098108	−0.058407	−1.766871	H	−2.097720	−0.058534	−1.766813
H	−2.653386	0.751543	−1.670764	H	−2.090019	1.702503	−0.316809	H	−2.090240	1.702499	−0.317090
**6a4**				**6a5**				**6a6**			
C	−1.757981	0.864267	0.312133	C	1.975704	−0.383931	0.255445	C	1.808200	−0.230822	0.111615

C	−2.975665	0.168146	−0.305943	C	2.508169	0.969699	−0.228568	C	1.757259	1.228987	0.587260
C	−2.636465	−1.316047	−0.236469	C	1.254404	1.805922	−0.422866	C	0.419819	1.723385	0.072056
O	−1.300152	−1.460193	−0.005593	O	0.167578	0.969043	−0.487138	O	0.024930	0.941185	−0.966498
C	−0.636519	−0.173686	0.084697	C	0.561985	−0.428475	−0.364018	C	0.843371	−0.246144	−1.102734
O	−3.374270	−2.257871	−0.363912	O	1.140608	2.99834	−0.515029	O	−0.259972	2.644926	0.454022
C	−1.447992	2.242893	−0.285733	C	2.852436	−1.579671	−0.11897	C	3.211861	−0.735463	−0.226917
O	−0.310472	2.865183	0.282016	O	4.176315	−1.462012	0.381296	O	4.105892	−0.638450	0.871158
C	2.642937	−0.813213	0.005753	C	−2.942537	−0.634615	−0.135616	C	−2.157433	−1.065564	0.269192
C	3.698019	−1.851982	−0.299384	C	−4.167006	−0.98611	−0.948881	C	−2.895384	−1.332181	1.556240
C	1.618255	−1.093857	0.826742	C	−1.810848	−1.342311	−0.270653	C	−0.980334	−1.656929	0.004130
C	0.426671	−0.234277	1.185730	C	−0.49387	−1.179556	0.451217	C	−0.101944	−1.455384	−1.210942
H	−0.153182	0.004380	−0.886315	H	0.608746	−0.832059	−1.385752	H	1.404071	−0.137646	−2.040987
C	2.868861	0.482142	−0.754365	C	−3.176429	0.54513	0.792334	C	−2.859071	−0.082229	−0.652984
O	2.256900	1.662548	−0.229937	O	−2.044059	1.128184	1.40333	O	−3.068207	1.182281	−0.036122
H	−1.908464	0.982053	1.392723	H	1.862766	−0.353399	1.349116	H	1.382602	−0.868103	0.897218
H	−3.926143	0.362700	0.194212	H	3.200263	1.467806	0.450958	H	1.833194	1.365977	1.666014
H	−3.101852	0.433392	−1.364691	H	3.014906	0.877191	−1.198116	H	2.548213	1.832430	0.123436
H	−2.296630	2.911364	−0.101845	H	2.952874	−1.633855	−1.209208	H	3.646331	−0.112304	−1.017225
H	−1.350900	2.143764	−1.380347	H	2.379352	−2.517908	0.210639	H	3.160781	−1.764723	−0.617080
H	0.490476	2.469619	−0.102570	H	4.138615	−1.527138	1.344828	H	3.814542	−1.260908	1.550668
H	4.692662	−1.523618	0.029853	H	−4.452929	−0.160757	−1.613459	H	−3.866857	−1.803724	1.357425
H	3.772562	−2.037122	−1.379400	H	−5.031588	−1.176518	−0.299342	H	−3.107502	−0.385335	2.063063
H	3.474436	−2.804682	0.185766	H	−4.005398	−1.876749	−1.561028	H	−2.330603	−1.981361	2.230910
H	1.606615	−2.094144	1.255707	H	−1.823548	−2.145091	−1.009249	H	−0.590860	−2.347191	0.752738
H	−0.056133	−0.631634	2.085233	H	−0.626157	−0.664366	1.403919	H	0.517270	−2.346424	−1.374507
H	0.709046	0.795620	1.404532	H	−0.083054	−2.176657	0.661717	H	−0.696059	−1.327301	−2.120997
H	2.479262	0.382029	−1.775346	H	−3.833354	0.221533	1.611829	H	−3.862970	−0.468302	−0.874260
H	3.951650	0.649103	−0.857937	H	−3.754456	1.300191	0.23037	H	−2.329566	0.021544	−1.605605
H	2.639823	1.833528	0.640863	H	−1.442623	1.412847	0.69656	H	−2.221799	1.657899	−0.012776
**6a7**				**6b1**				**6b2**			
C	1.841732	0.119573	0.472053	C	−1.021268	0.860014	−0.370334	C	−2.176031	−0.063884	0.304773
C	1.507341	1.577287	0.825274	C	−2.395320	0.513100	−0.954254	C	−2.430712	1.399230	−0.081533
C	0.214724	1.847613	0.077302	C	−2.736706	−0.823711	−0.307541	C	−1.034947	1.991661	−0.208981
O	0.089864	0.956416	−0.936977	O	−1.943340	−1.002672	0.788104	O	−0.131140	0.975160	−0.321153
C	1.043050	−0.129740	−0.836145	C	−1.064114	0.136807	0.991840	C	−0.779989	−0.330806	−0.302221
O	−0.621669	2.694626	0.278849	O	−3.558614	−1.633600	−0.648525	O	−0.701301	3.146691	−0.220741
C	3.340143	−0.160114	0.331682	C	−0.718684	2.357888	−0.271990	C	−3.262429	−1.029887	−0.171817
O	3.617134	−1.515481	−0.001486	O	0.503516	2.638670	0.382816	O	−2.986956	−2.390240	0.138501
C	−2.003645	−1.164325	0.265307	C	2.178895	−1.142594	0.047652	C	2.590480	−0.911875	−0.004562
C	−2.868569	−1.378231	1.481399	C	2.771730	−2.208181	−0.844945	C	3.822640	−1.205928	−0.820916
C	−0.740991	−1.621730	0.218787	C	0.996775	−1.320104	0.659433	C	1.434598	−1.558715	−0.218247
C	0.260270	−1.453044	−0.902592	C	0.272809	−0.357109	1.560438	C	0.109263	−1.314422	0.461491
H	1.698405	−0.046337	−1.715114	H	−1.540322	0.784710	1.740806	H	−0.878589	−0.652142	−1.348029
C	−2.688578	−0.397494	−0.853360	C	2.992657	0.129838	0.173236	C	2.753615	0.220262	0.991481
O	−3.139192	0.885509	−0.435096	O	2.619931	1.141990	−0.787555	O	2.838214	1.482658	0.337685
H	1.446945	−0.539465	1.251282	H	−0.244063	0.384098	−0.981391	H	−2.092288	−0.132351	1.399200

H	1.380905	1.774617	1.890826	H	−2.436345	0.430912	−2.041897	H	−3.024658	1.973761	0.631397
H	2.264455	2.277027	0.445631	H	−3.159954	1.240866	−0.648830	H	−2.921562	1.479180	−1.061033
H	3.837632	0.027756	1.288809	H	−1.505228	2.851138	0.313535	H	−3.337853	−0.984691	−1.264441
H	3.780248	0.532015	−0.403969	H	−0.751010	2.799127	−1.282067	H	−4.236963	−0.716364	0.231823
H	3.523493	−1.619129	−0.956434	H	1.226742	2.246242	−0.141516	H	−3.085571	−2.505971	1.092850
H	−3.740617	−1.998244	1.234513	H	3.732113	−2.565811	−0.451579	H	4.637790	−1.576218	−0.185213
H	−3.260026	−0.417721	1.831346	H	2.977057	−1.825304	−1.854608	H	4.182860	−0.283409	−1.289767
H	−2.323739	−1.862212	2.296461	H	2.105284	−3.068075	−0.947851	H	3.634215	−1.947182	−1.602370
H	−0.374603	−2.162710	1.090967	H	0.472509	−2.259426	0.487134	H	1.414404	−2.317311	−1.001529
H	0.997425	−2.263470	−0.861313	H	0.058934	−0.852162	2.516735	H	−0.456407	−2.249955	0.530222
H	−0.219260	−1.509232	−1.884763	H	0.859826	0.538750	1.772892	H	0.234720	−0.934872	1.481329
H	−3.595918	−0.942787	−1.145271	H	4.062572	−0.094561	0.064942	H	3.700868	0.102372	1.530891
H	−2.049208	−0.326347	−1.739499	H	2.855907	0.607165	1.144530	H	1.951972	0.213928	1.741438
H	−2.368085	1.468922	−0.344029	H	2.653386	0.751543	−1.670764	H	1.967597	1.643260	−0.057928
**6b3**				**6b4**				**6b5**			
C	−2.075708	−0.251655	0.659098	C	−1.024496	0.824061	−0.481019	C	1.086823	0.828516	0.089629
C	−2.476319	1.130220	0.129877	C	−2.344317	0.270878	−1.031077	C	2.588993	1.039565	0.332392
C	−1.154198	1.799100	−0.197020	C	−2.601455	−0.975870	−0.193261	C	3.212878	−0.342413	0.229505
O	−0.177625	0.838649	−0.275139	O	−1.818587	−0.935234	0.921766	O	2.303429	−1.211403	−0.28742
C	−0.712172	−0.488632	−0.028676	C	−1.002806	0.265219	0.958668	C	1.014344	−0.591868	−0.542477
O	−0.907666	2.960895	−0.379284	O	−3.363660	−1.880439	−0.414355	O	4.33089	−0.677812	0.523479
C	−3.113863	−1.352770	0.401427	C	−0.892245	2.349054	−0.550295	C	0.477881	1.944623	−0.770261
O	−3.540539	−1.421836	−0.953167	O	0.214691	2.856543	0.171731	O	−0.882891	1.756588	−1.082832
C	2.703276	−0.831140	0.023679	C	2.123790	−1.151143	−0.084951	C	−2.517545	−0.853207	0.013419
C	3.875224	−1.176959	−0.857802	C	2.631170	−2.432183	−0.713514	C	−3.825863	−0.775791	−0.736004
C	1.582342	−1.568282	0.023447	C	1.087076	−1.183727	0.768109	C	−1.423096	−1.387724	−0.547001
C	0.307614	−1.292283	0.783168	C	0.381926	−0.084113	1.523690	C	−0.044792	−1.550677	0.035227
H	−0.856737	−0.954107	−1.014162	H	−1.491757	0.971251	1.642978	H	0.900474	−0.523136	−1.631395
C	2.873030	0.450576	0.817889	C	2.917765	0.044958	−0.575121	C	−2.51947	−0.208289	1.378798
O	2.787934	1.596295	−0.022249	O	2.635868	1.326760	−0.017794	O	−2.471924	1.236774	1.291947
H	−1.906788	−0.197713	1.743300	H	−0.193931	0.363813	−1.031397	H	0.562202	0.809636	1.053386
H	−3.054471	1.744659	0.822426	H	−2.338895	0.017918	−2.092916	H	2.838275	1.490116	1.295243
H	−3.052035	1.027143	−0.797886	H	−3.178609	0.965371	−0.862716	H	3.045141	1.6683	−0.443553
H	−4.012749	−1.138301	0.988174	H	−1.781971	2.810210	−0.103092	H	1.014187	1.990318	−1.727104
H	−2.727759	−2.322302	0.749860	H	−0.867978	2.662078	−1.607192	H	0.653917	2.907683	−0.261389
H	−2.880018	−1.907806	−1.462111	H	1.025249	2.418267	−0.142140	H	−1.37985	1.680878	−0.247208
H	4.767440	−1.398080	−0.257393	H	3.671253	−2.634858	−0.426026	H	−4.140603	0.266062	−0.884285
H	4.125162	−0.317620	−1.489894	H	2.616427	−2.373937	−1.809890	H	−4.632111	−1.276916	−0.184308
H	3.671722	−2.038616	−1.499625	H	2.024999	−3.290141	−0.415568	H	−3.753963	−1.234155	−1.725048
H	1.547388	−2.439100	−0.632505	H	0.653023	−2.166759	0.944604	H	−1.522221	−1.759981	−1.567067
H	−0.167605	−2.242081	1.062734	H	0.241827	−0.412669	2.561228	H	−0.036104	−1.459861	1.126078
H	0.492993	−0.750098	1.715925	H	0.937418	0.850816	1.546193	H	0.30577	−2.564745	−0.191378
H	3.876617	0.478859	1.258286	H	2.746252	0.156706	−1.653446	H	−1.642099	−0.485	1.963714
H	2.156446	0.509781	1.647617	H	3.991280	−0.177801	−0.467409	H	−3.407361	−0.507546	1.952572
H	1.868214	1.644304	−0.326550	H	2.940470	1.335895	0.898638	H	−3.342602	1.54875	1.010989
**6b6**				**6b7**				**6b8**			

C	–2.050706	–0.317192	0.259415	C	1.683183	0.718520	0.711806	C	–2.050678	–0.317166	0.259412
C	–2.394925	1.127952	–0.127411	C	2.865577	0.302871	–0.169392	C	–2.394911	1.127900	–0.127986
C	–1.040028	1.812238	–0.237868	C	2.647486	–1.190206	–0.377414	C	–1.040059	1.812219	–0.237979
O	–0.068962	0.856445	–0.342153	O	1.343929	–1.491521	–0.089102	O	–0.068915	0.856399	–0.341955
C	–0.627215	–0.487251	–0.316066	C	0.613753	–0.283626	0.245586	C	–0.627171	–0.487260	–0.316059
O	–0.781463	2.985669	–0.250387	O	3.435716	–2.027215	–0.731630	O	–0.781429	2.985642	–0.250277
C	–3.040242	–1.372898	–0.239085	C	1.244066	2.187473	0.591182	C	–3.040202	–1.372981	–0.238772
O	–4.314033	–1.263742	0.375712	O	0.769133	2.527974	–0.698424	O	–4.314038	–1.263626	0.375941
C	2.766538	–0.869158	0.037134	C	–2.755591	–0.677426	0.113379	C	2.766524	–0.869045	0.037022
C	4.023156	–1.116230	–0.756652	C	–3.887919	–1.451678	–0.516788	C	4.023269	–1.116051	–0.756652
C	1.652402	–1.588016	–0.168042	C	–1.681616	–1.291322	0.636536	C	1.652453	–1.587915	–0.168346
C	0.307349	–1.394605	0.489646	C	–0.492994	–0.602860	1.254193	C	0.307411	–1.394739	0.489435
H	–0.672688	–0.830106	–1.359479	H	0.161819	0.088079	–0.678277	H	–0.672793	–0.830020	–1.359527
C	2.852016	0.301806	0.997865	C	–2.889666	0.835841	0.099510	C	2.851960	0.301724	0.997946
O	2.866499	1.544388	0.302817	O	–1.986875	1.492803	–0.816441	O	2.866127	1.544421	0.303159
H	–1.998883	–0.391915	1.352783	H	1.925862	0.517974	1.765387	H	–1.998779	–0.391401	1.352810
H	–3.029884	1.662497	0.581271	H	3.855294	0.491660	0.250327	H	–3.030306	1.662533	0.580228
H	–2.873285	1.183754	–1.115807	H	2.803839	0.803207	–1.144232	H	–2.872784	1.183177	–1.116647
H	–2.676071	–2.374993	0.010334	H	2.105423	2.832695	0.799838	H	–2.676136	–2.375020	0.011004
H	–3.114376	–1.318349	–1.338200	H	0.491987	2.407796	1.364232	H	–3.114280	–1.318761	–1.337907
H	–4.737097	–0.458651	0.050105	H	–0.172303	2.285964	–0.739212	H	–4.737113	–0.458697	0.049949
H	4.849940	–1.418236	–0.100383	H	–4.047491	–1.155913	–1.562585	H	4.849915	–1.418296	–0.100293
H	4.334759	–0.188759	–1.249858	H	–4.835640	–1.265068	0.004914	H	4.335099	–0.188514	–1.249550
H	3.888360	–1.890666	–1.516698	H	–3.699425	–2.527983	–0.499227	H	3.888551	–1.890319	–1.516888
H	1.684812	–2.370906	–0.926684	H	–1.628449	–2.377169	0.578136	H	1.684948	–2.370683	–0.927127
H	–0.192405	–2.365881	0.599192	H	–0.798809	0.345591	1.712102	H	–0.192212	–2.366109	0.598794
H	0.397227	–0.974040	1.496387	H	–0.062908	–1.219684	2.051274	H	0.397335	–0.974378	1.496271
H	3.799817	0.257936	1.547015	H	–2.654466	1.266486	1.076815	H	3.799877	0.257879	1.546921
H	2.045980	0.271440	1.742243	H	–3.921862	1.122111	–0.141264	H	2.046045	0.271049	1.742470
H	1.987400	1.644914	–0.093749	H	–2.095761	1.082411	–1.685048	H	1.987093	1.644749	–0.093578

III. Chiral HPLC separation profiles of 1a/1b 5a/5b

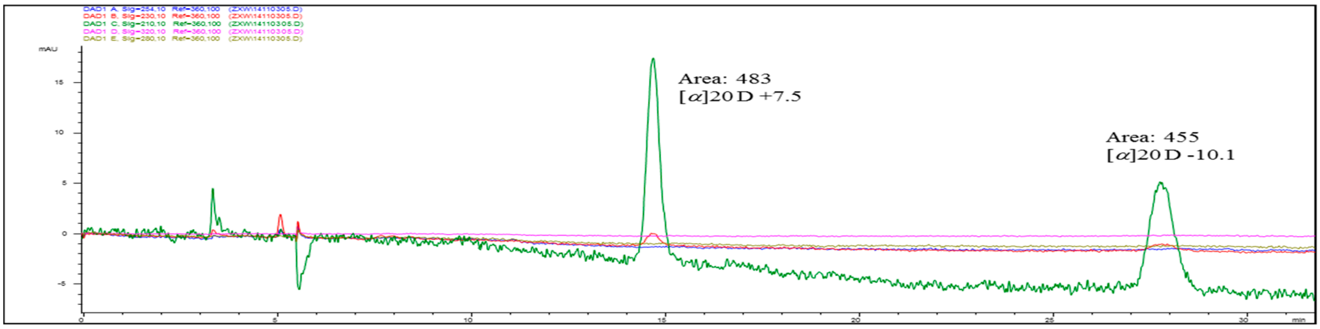

Figure S3. Chiral HPLC separation of compounds **1a** (left) and **1b** (right).

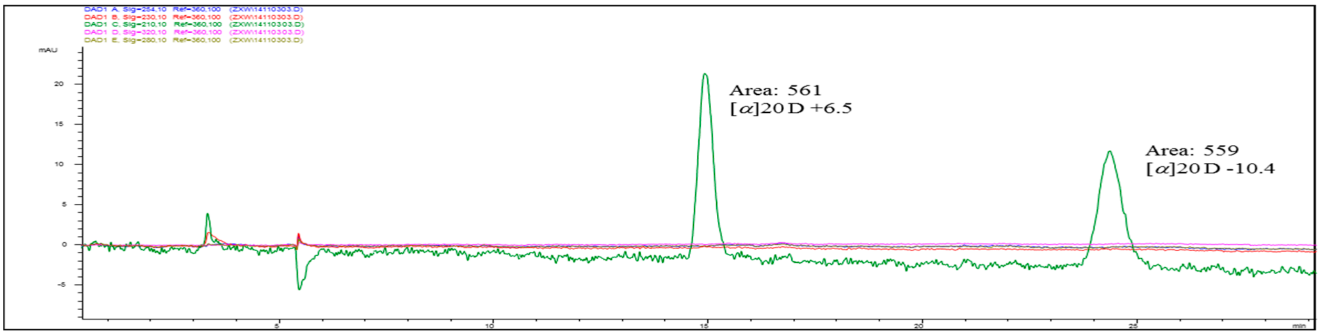

Figure S4. Chiral HPLC separation of compounds **2a** (left) and **2b** (right).

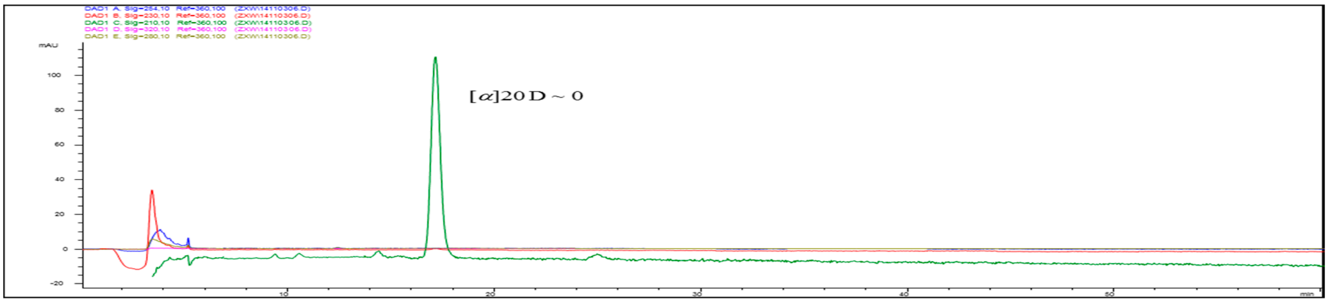

Figure S5. Chiral HPLC analysis of compounds **3a/3b**.

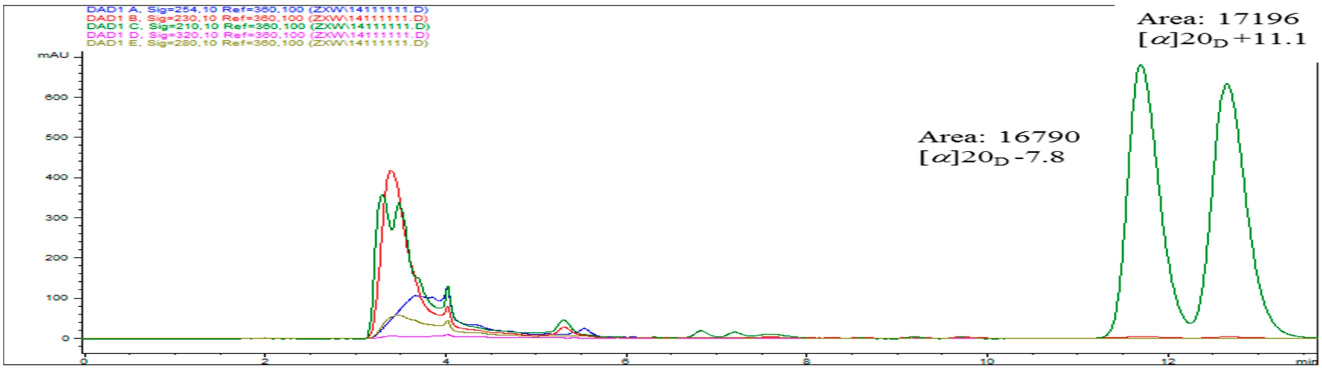

Figure S6. Chiral HPLC separation of the LAH reduction products of compound **3** (**3a-r** left and **3b-r** right).

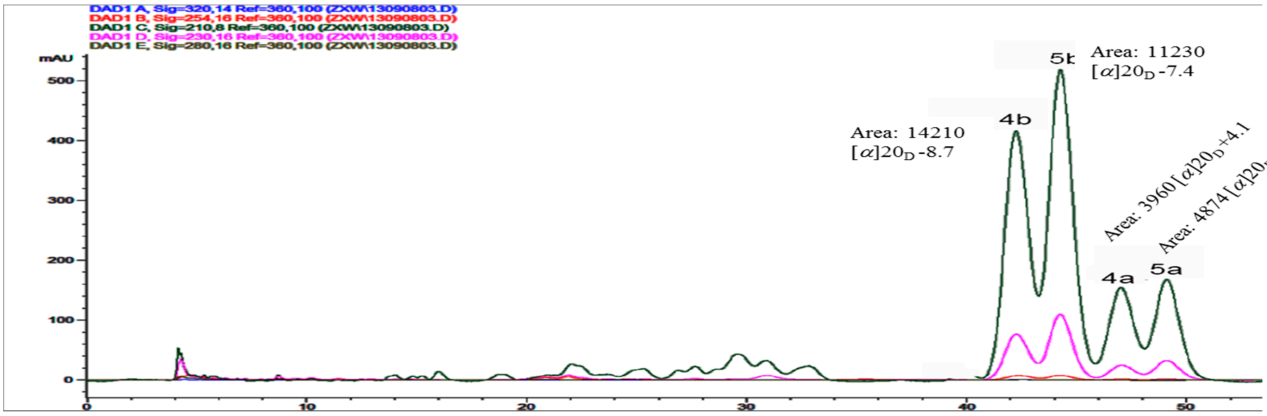

Figure S7. Chiral HPLC separation of compounds **4a/4b** and **5a/5b**.

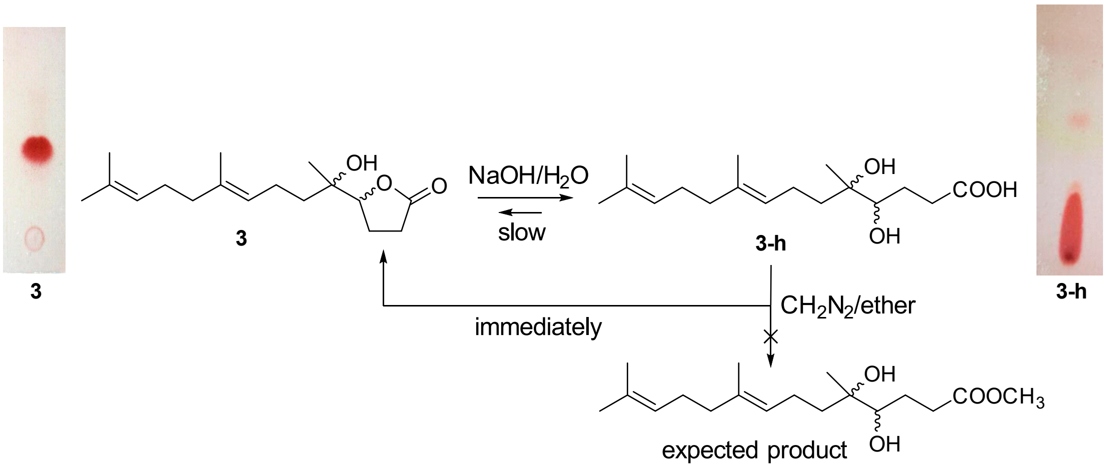

Scheme S1. NaOH hydrolysis reaction of **3** and CH_2N_2 diazotization of the hydrolyzates.

IV. HR-MS, 1D and 2D NMR spectra of the new compounds

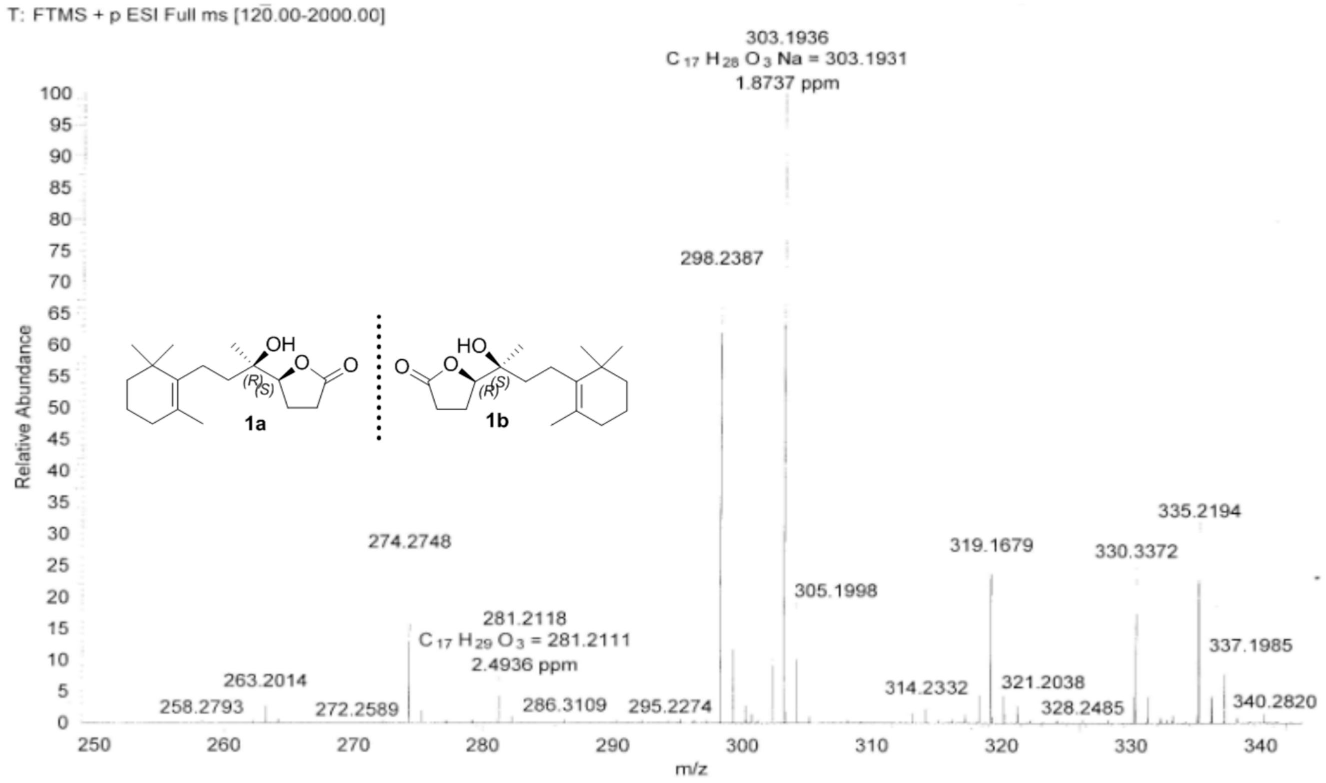

Figure S8. HR-ESI-MS of compounds **1a/1b**.

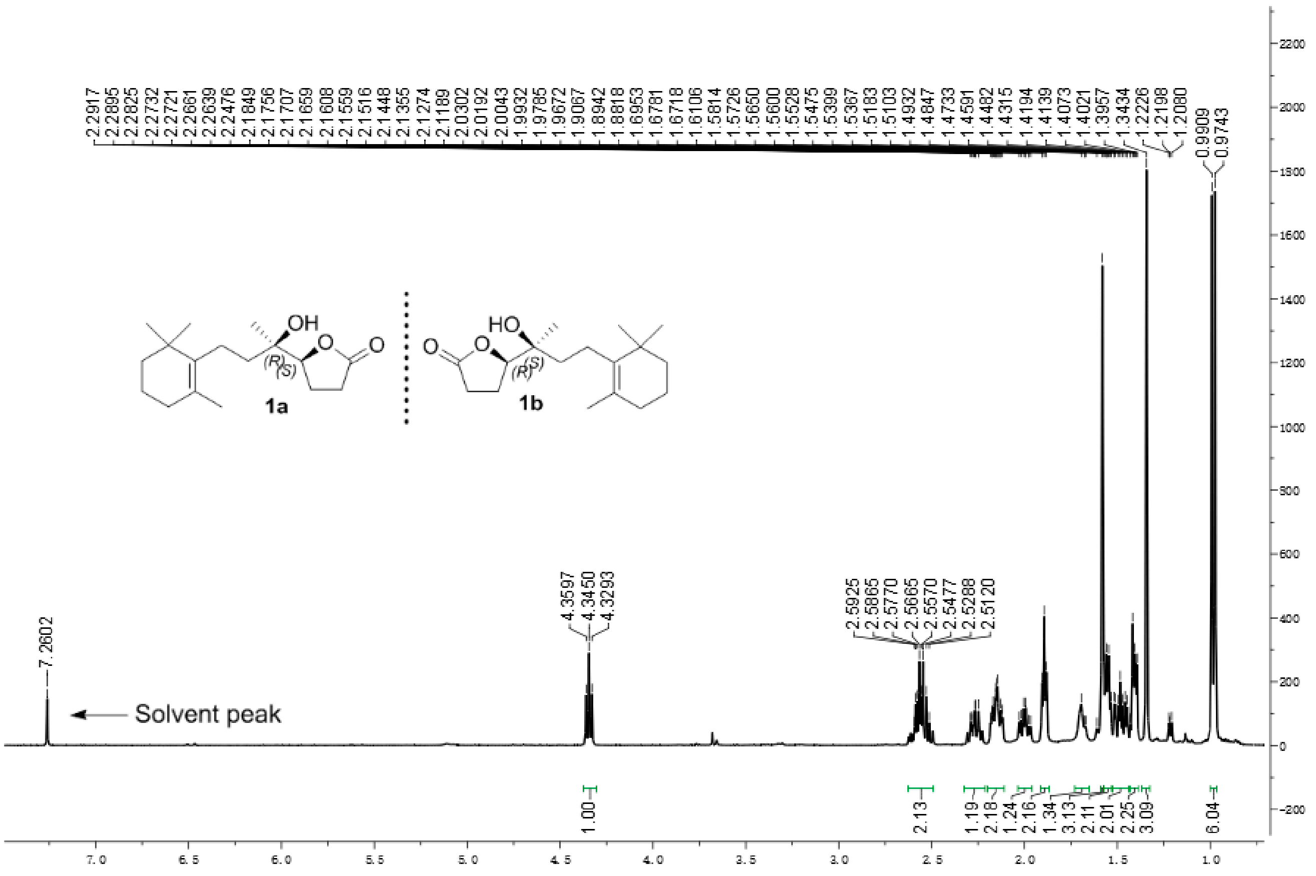

Figure S9. ^{1}H NMR spectrum (500 MHz, $CDCl_3$) of compounds **1a/1b**.

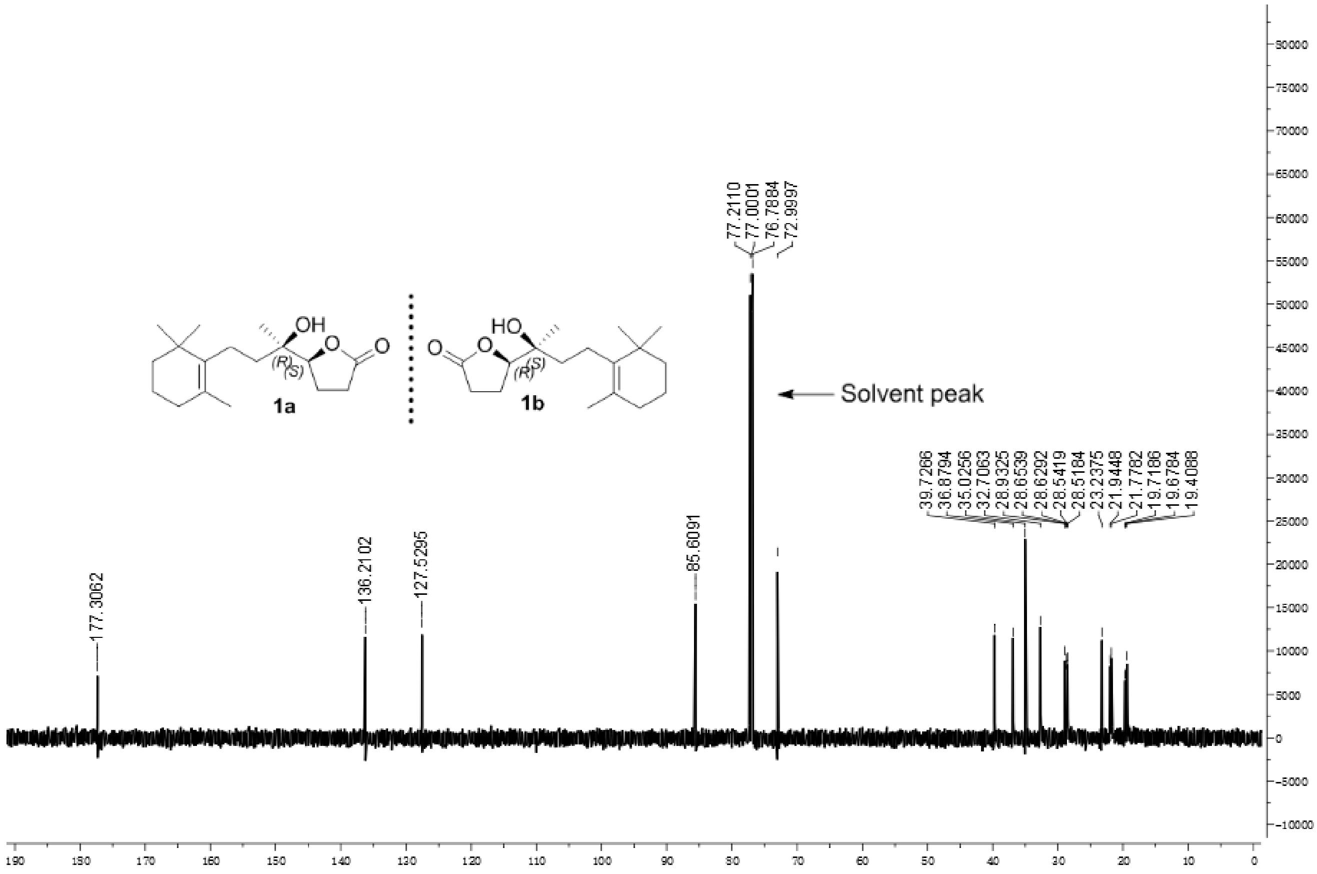

Figure S10. ^{13}C NMR spectrum (125 MHz, $CDCl_3$) of compounds **1a/1b**.

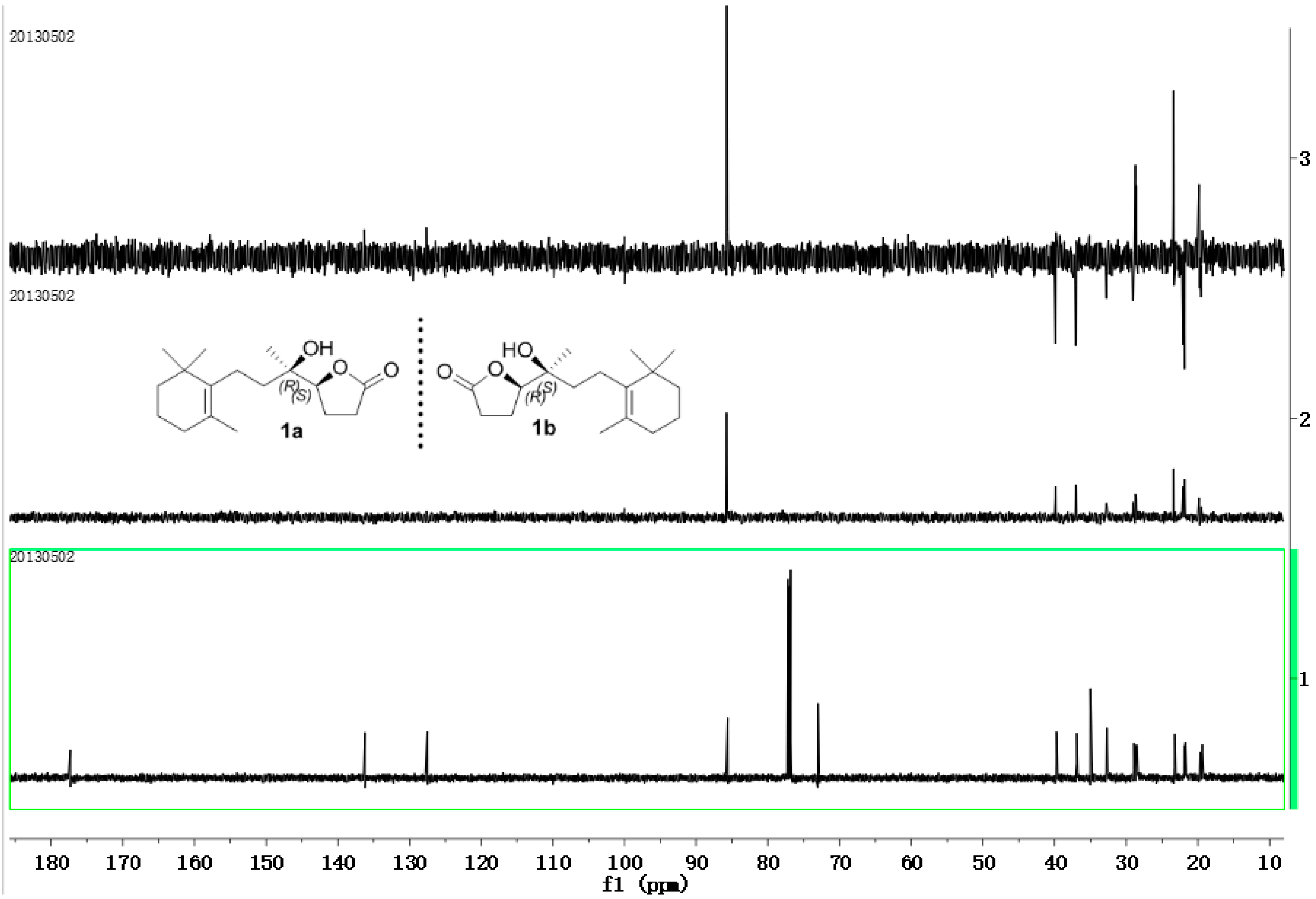

Figure S11. DEPT spectrum (125 MHz, $CDCl_3$) of compounds **1a/1b**.

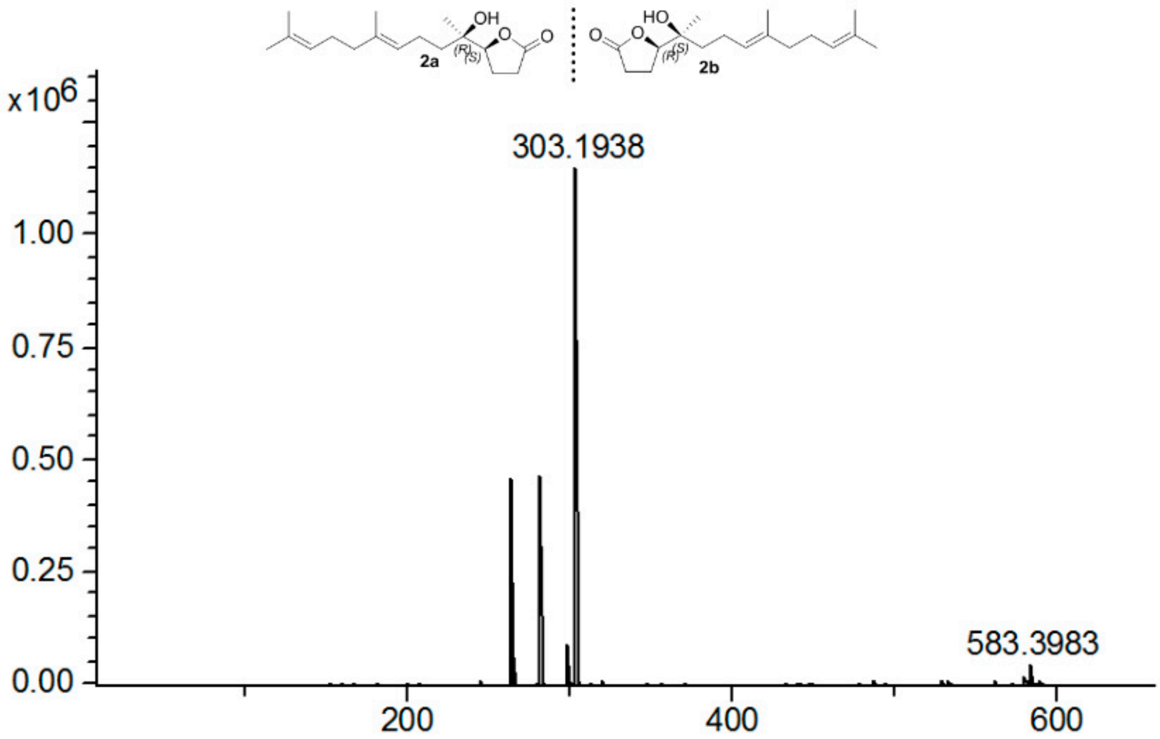

Figure S12. HR-ESI-MS of compounds **2a/2b**.

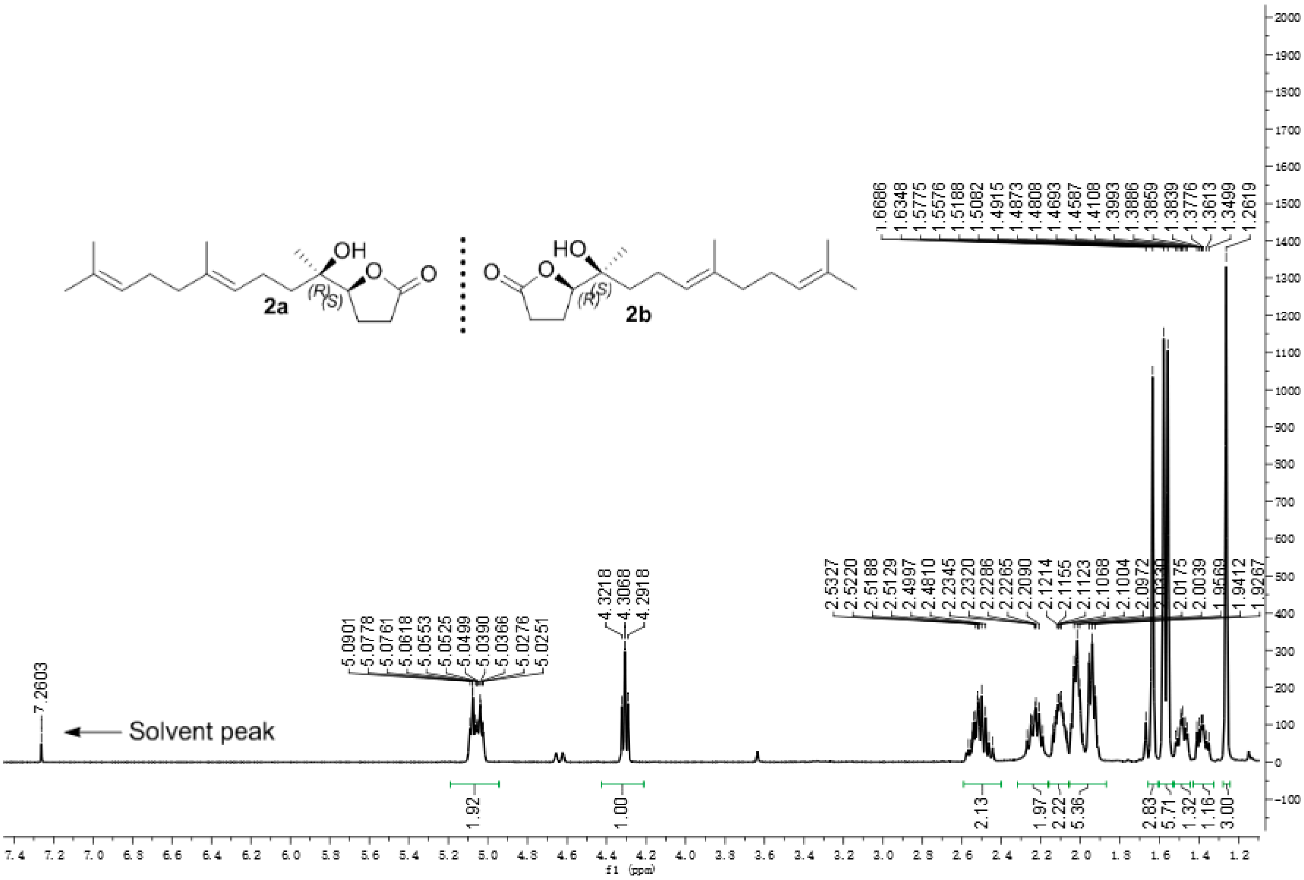

Figure S13. ^{1}H NMR spectrum (500 MHz, $CDCl_3$) of compounds **2a/2b**.

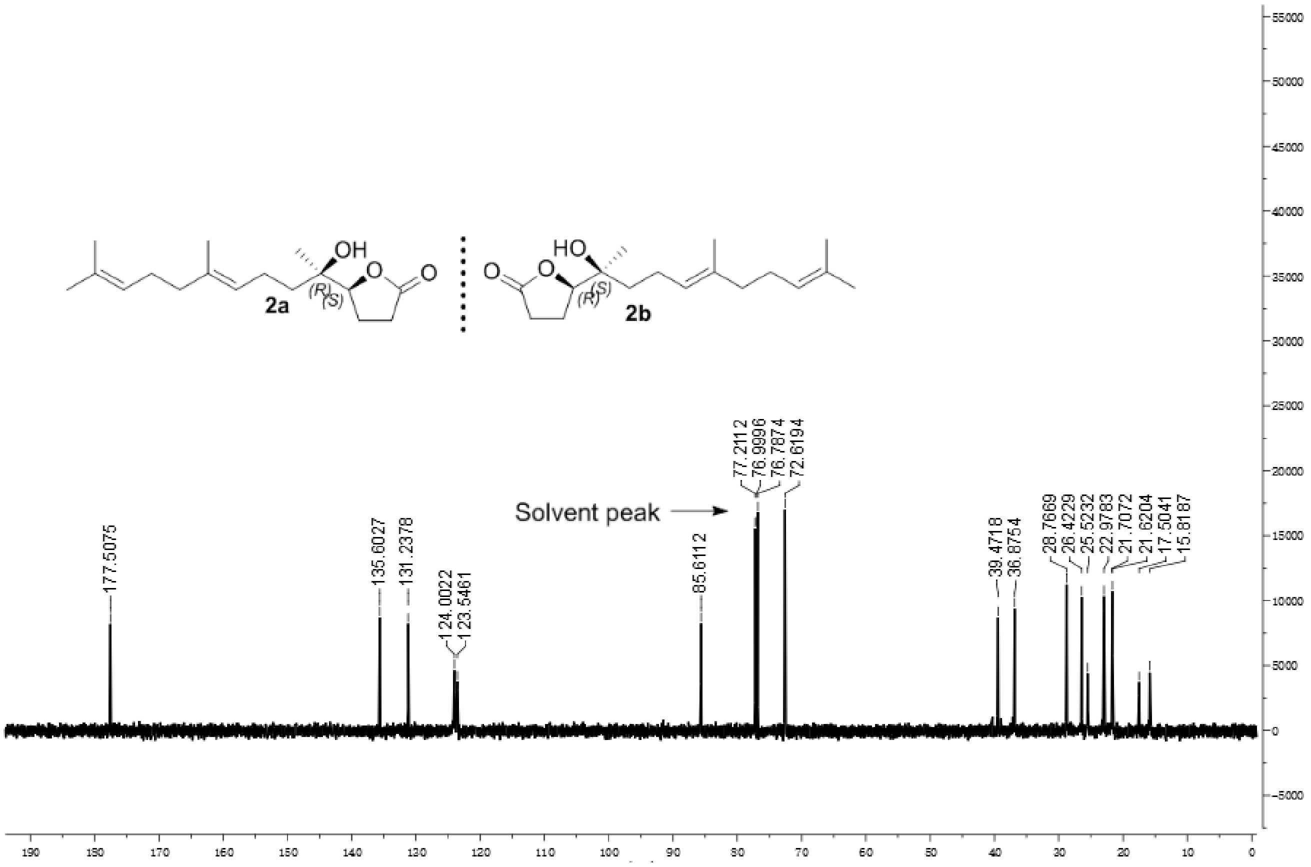

Figure S14. ^{13}C NMR spectrum (125 MHz, $CDCl_3$) of compounds **2a/2b**.

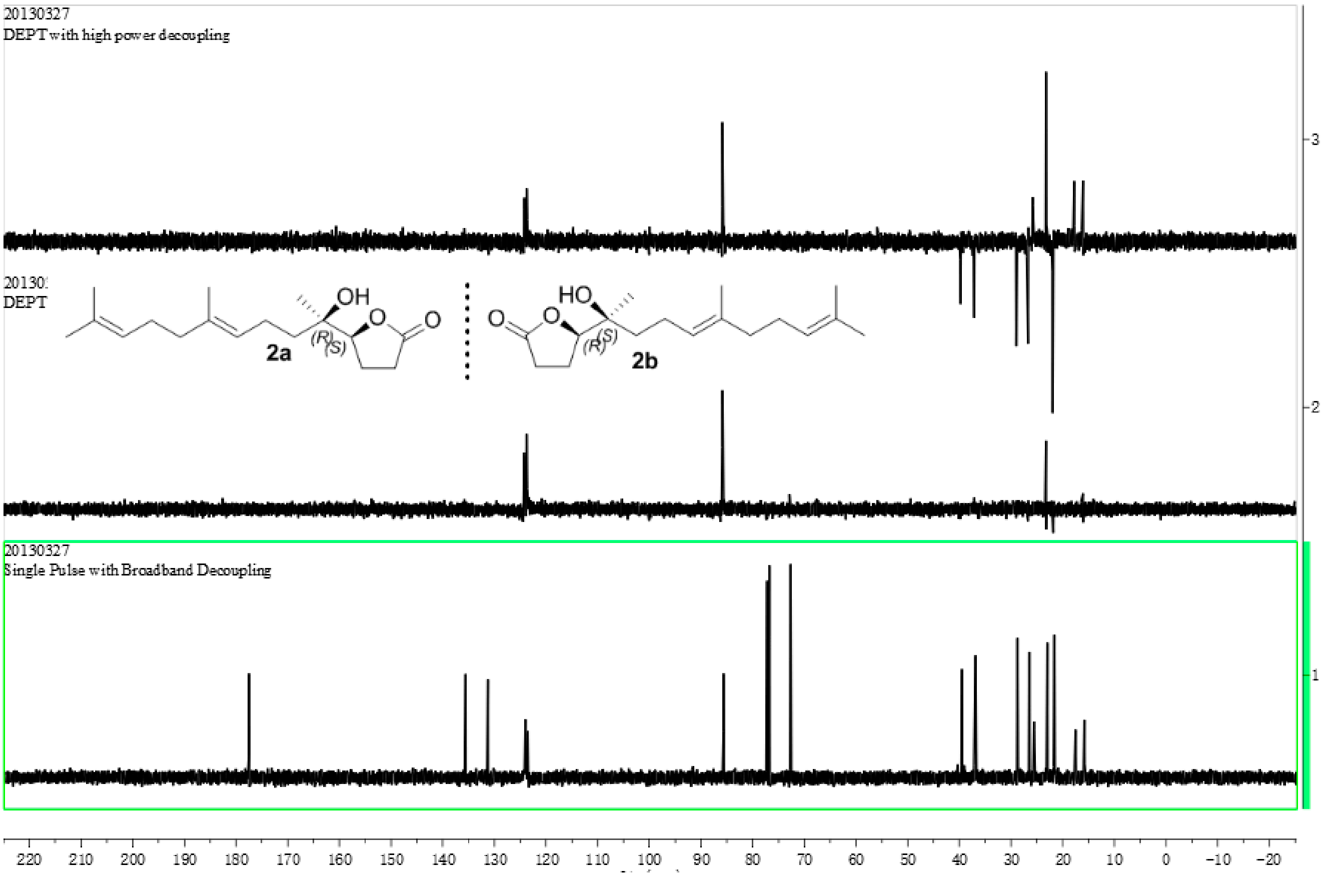

Figure S15. DEPT spectrum (125 MHz, $CDCl_3$) of compounds **2a/2b**.

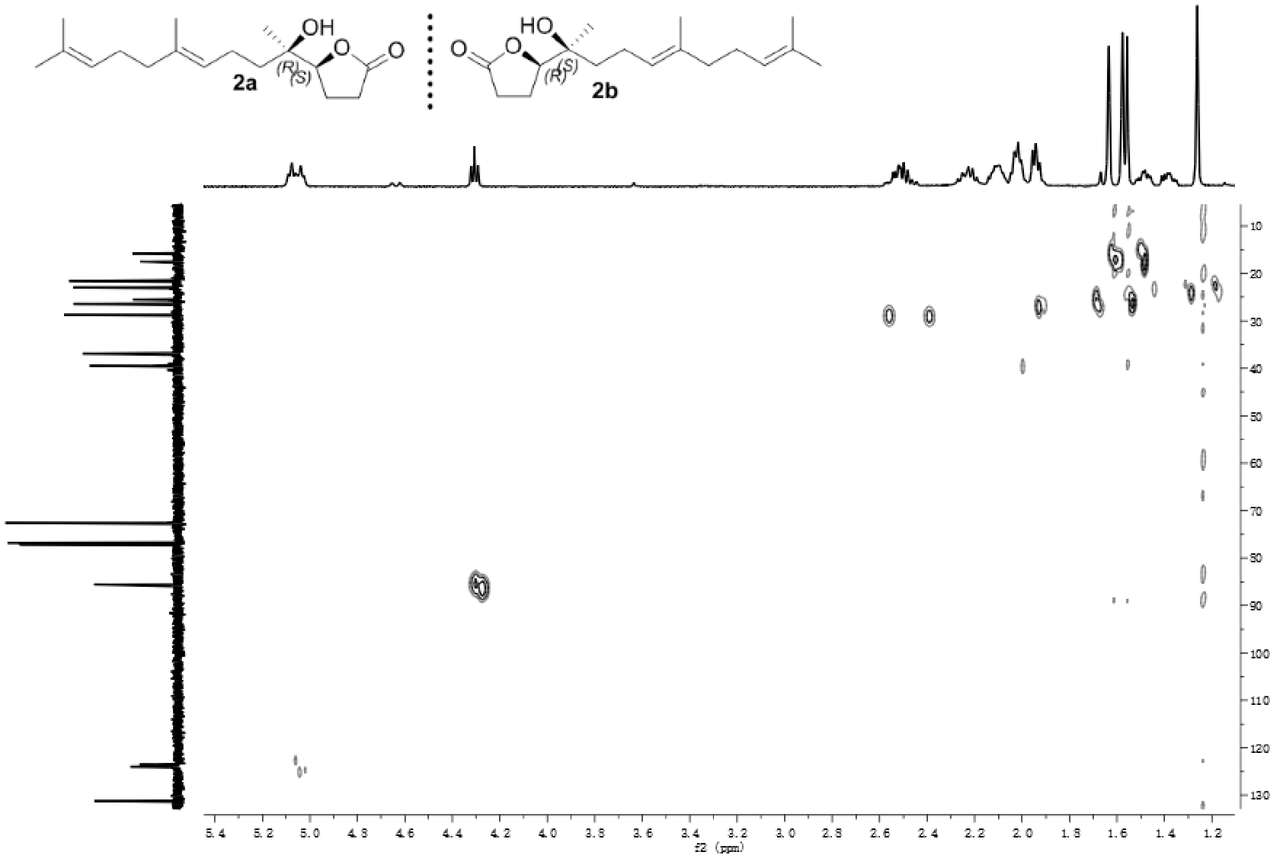

Figure S16. HMQC spectrum of compounds **2a/2b**.

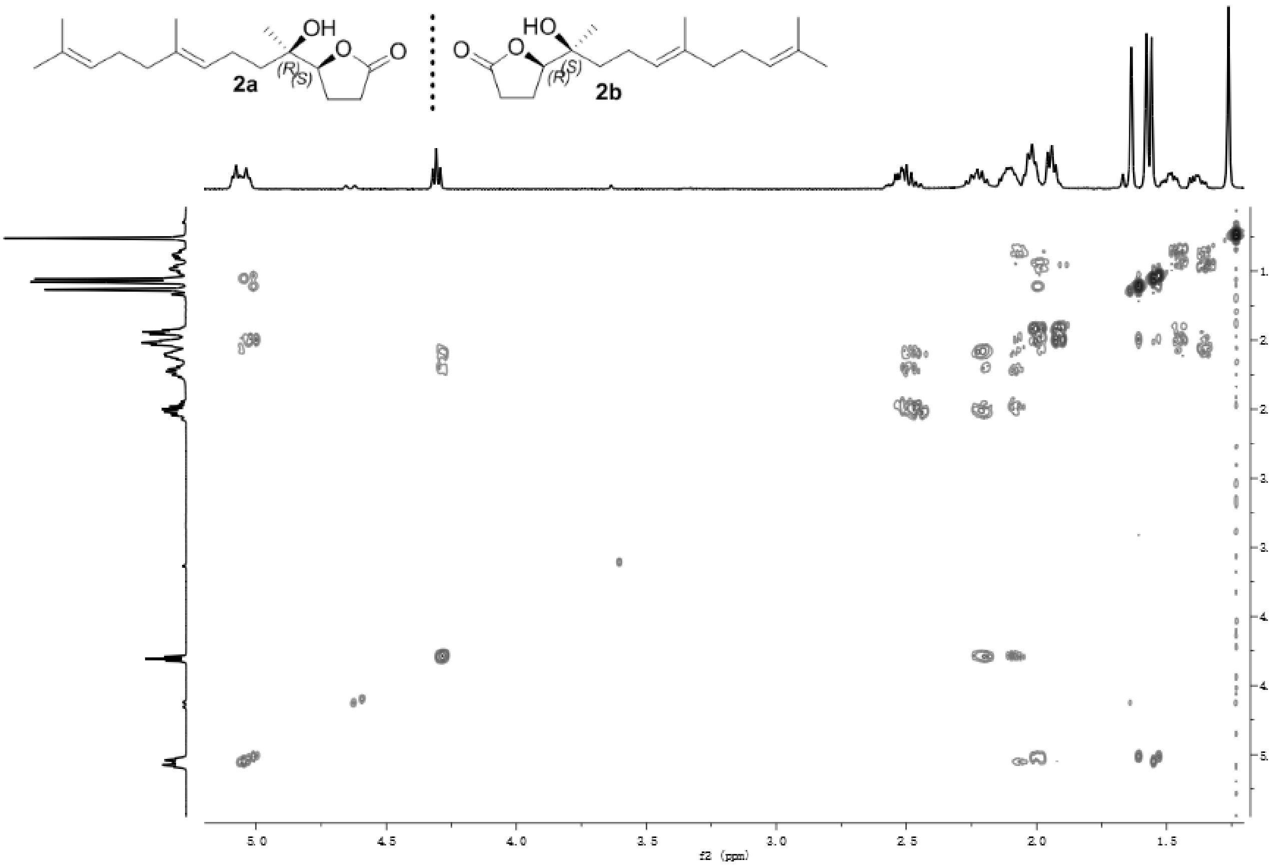

Figure S17. ^{1}H-^{1}H COSY spectrum of compounds **2a/2b**.

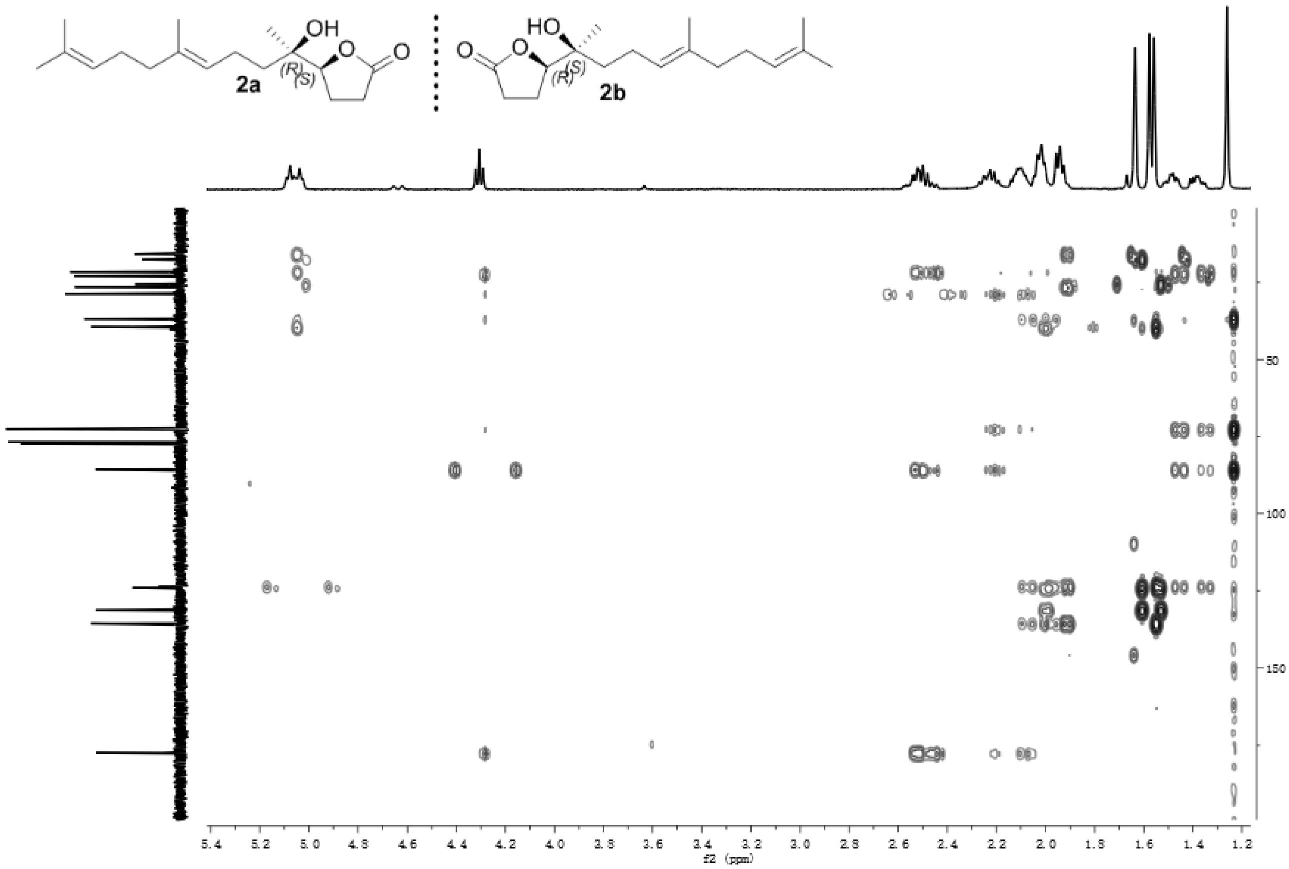

Figure S18. HMBC spectrum of compounds **2a/2b**.

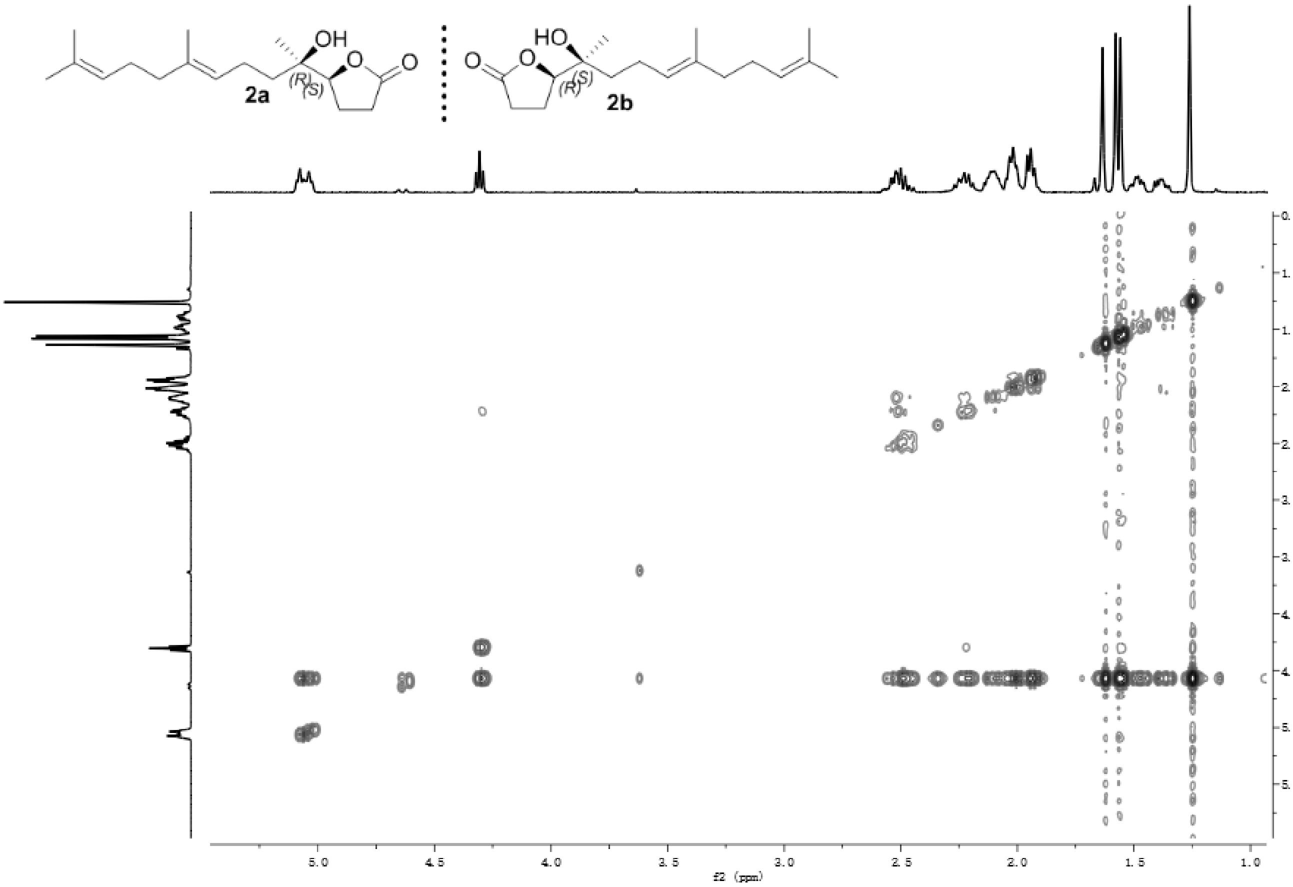

Figure S19. NOESY spectrum of compounds **2a/2b**.

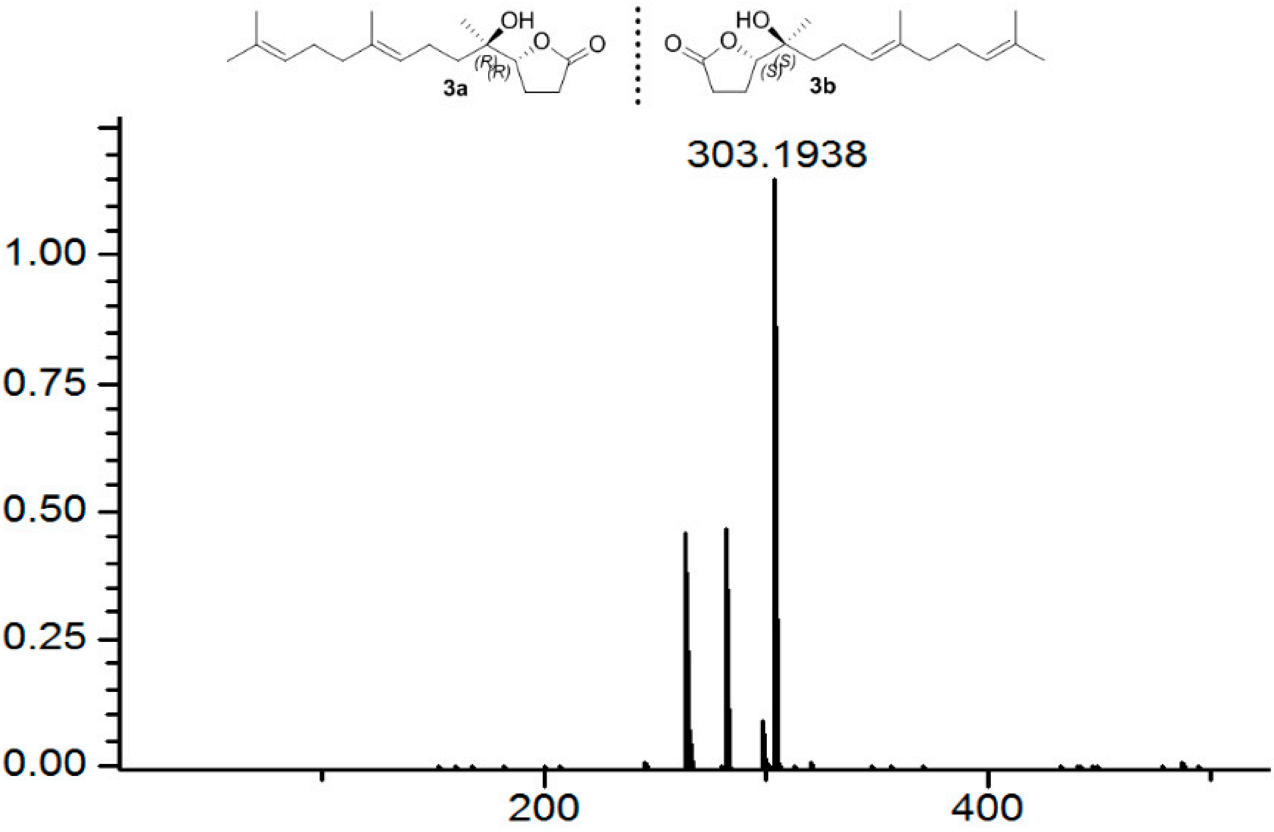

Figure S20. HR-ESI-MS of compounds **3a/3b**.

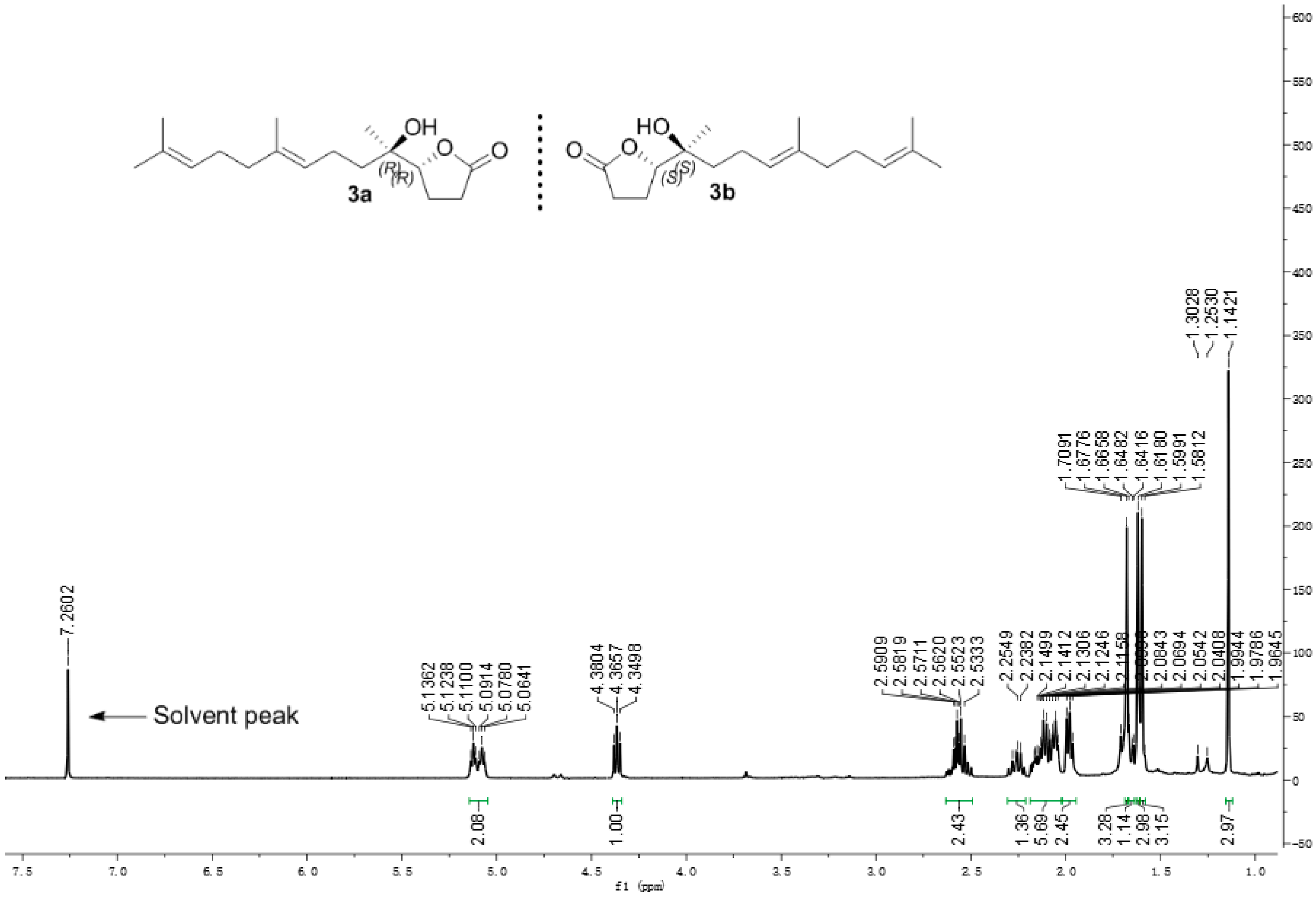

Figure S21. ^{1}H NMR spectrum (500 MHz, $CDCl_3$) of compounds **3a/3b**.

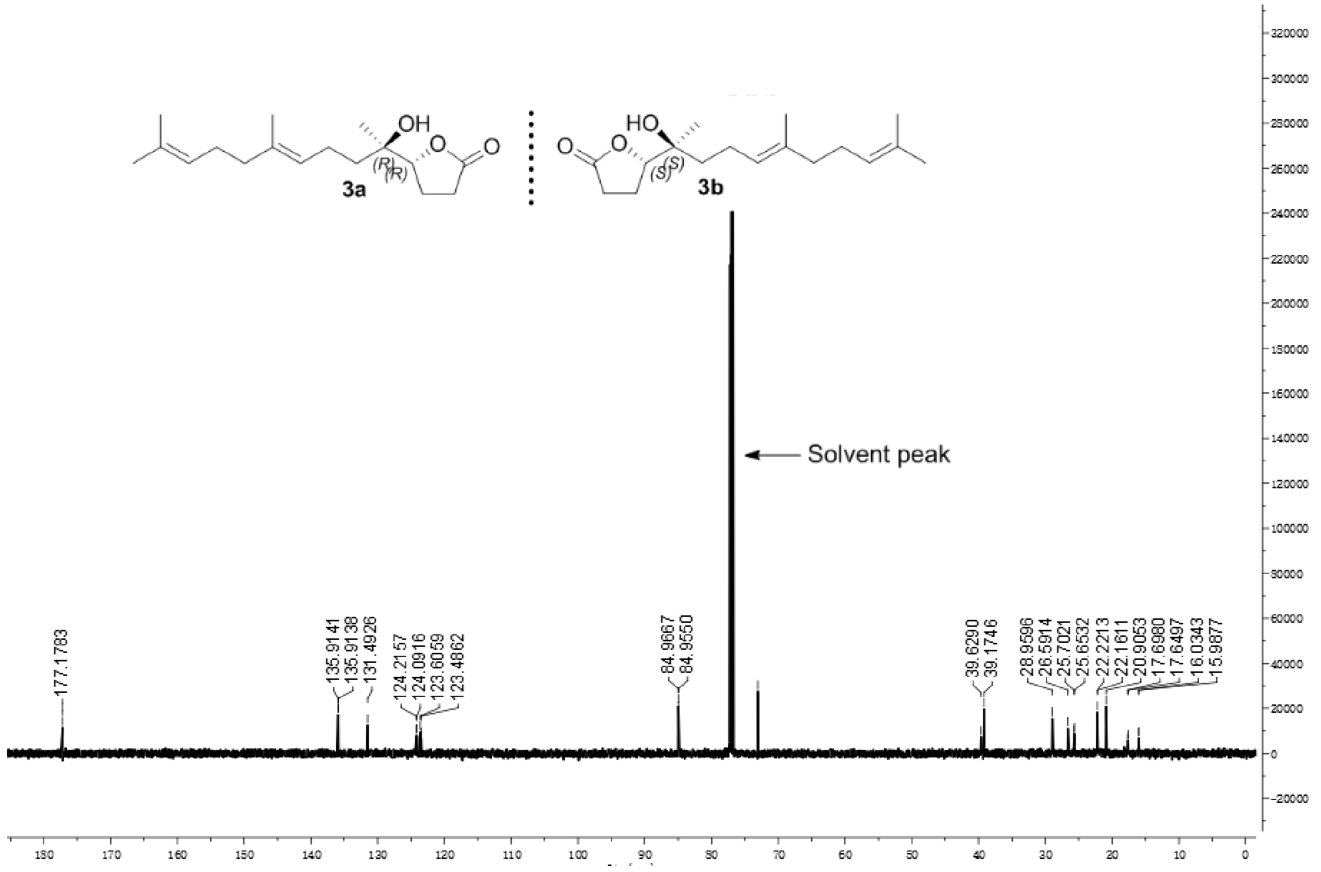

Figure S22. ^{13}C NMR spectrum (125 MHz, CDCl$_3$) of compounds **3a/3b**.

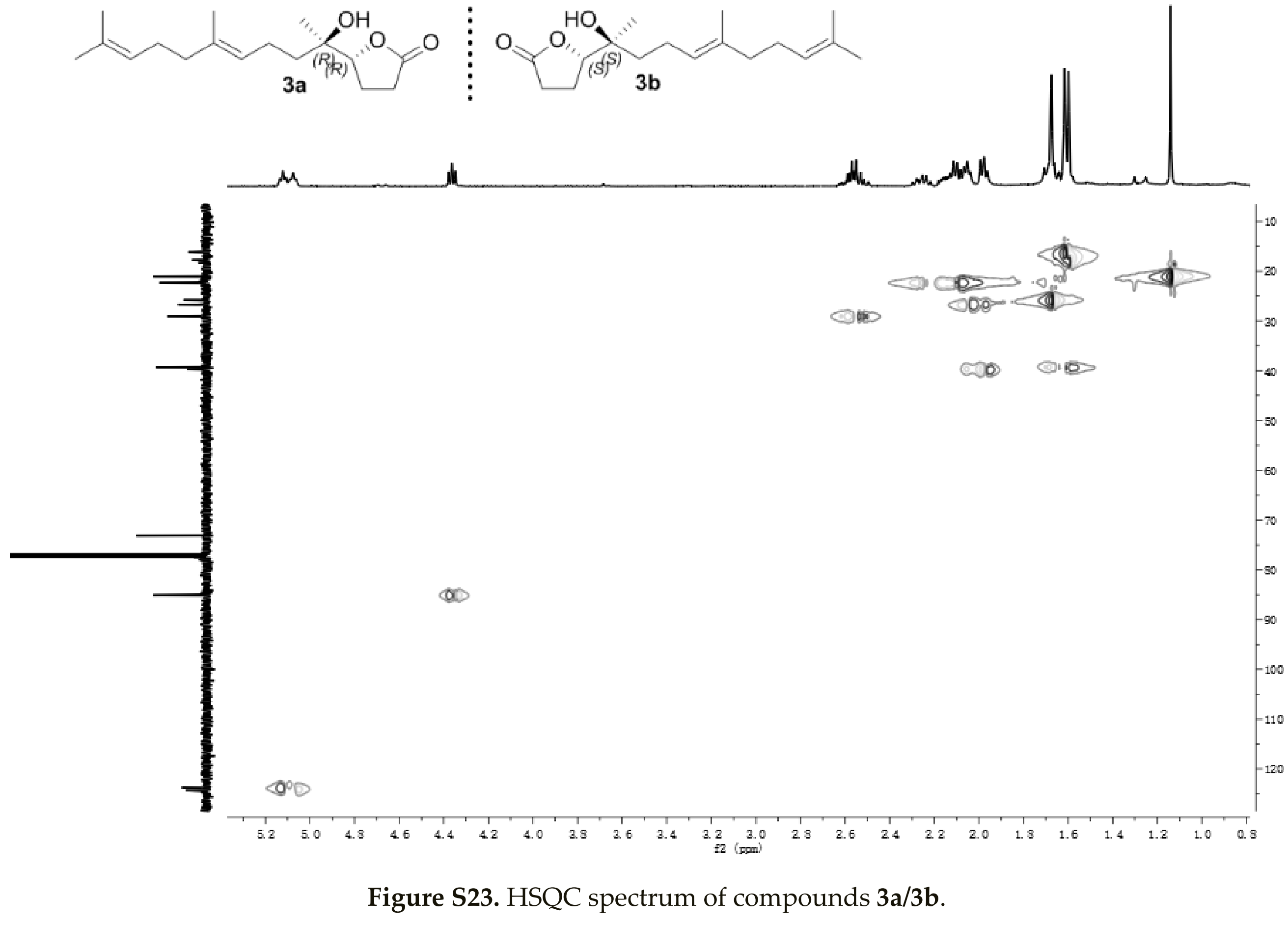

Figure S23. HSQC spectrum of compounds **3a/3b**.

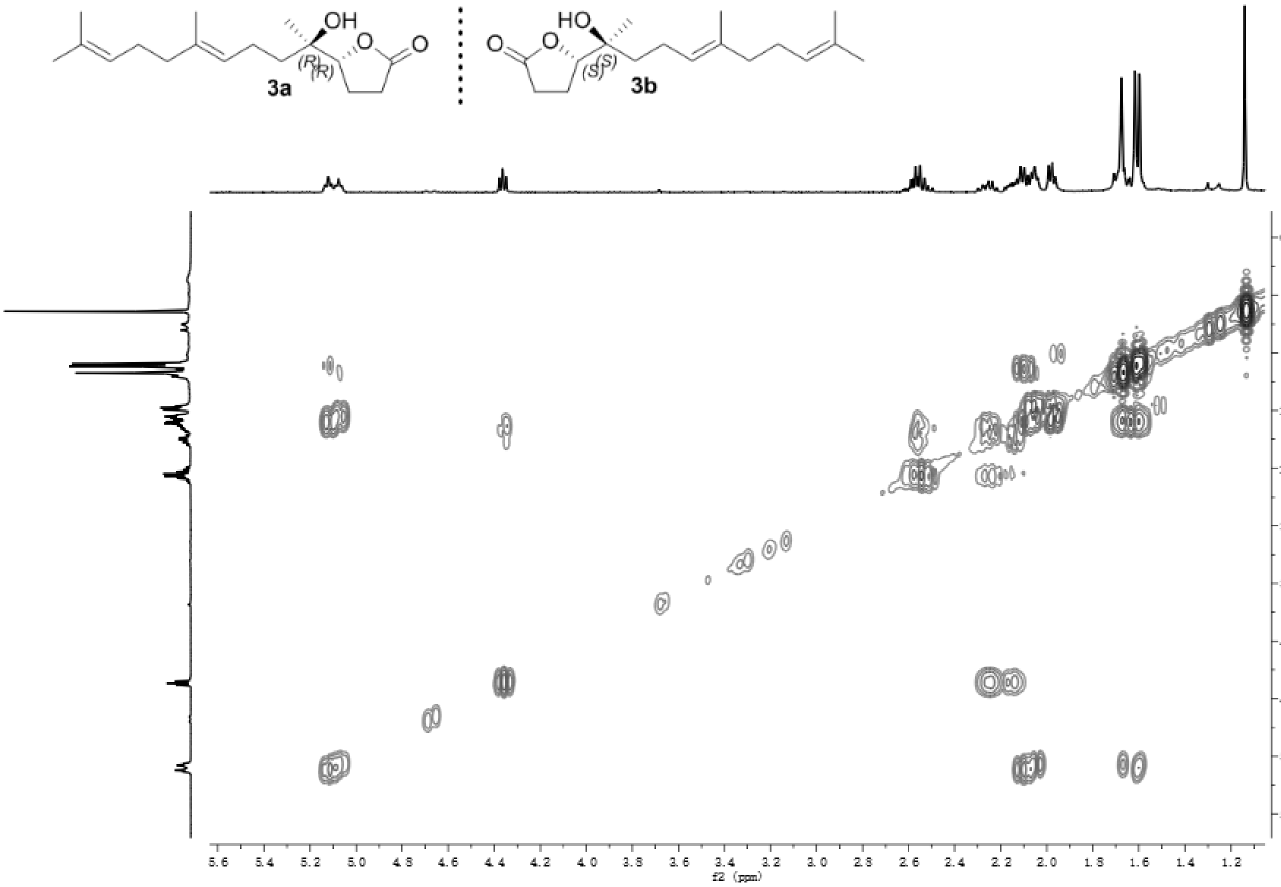

Figure S24. ^{1}H-^{1}H COSY spectrum of compounds **3a/3b**.

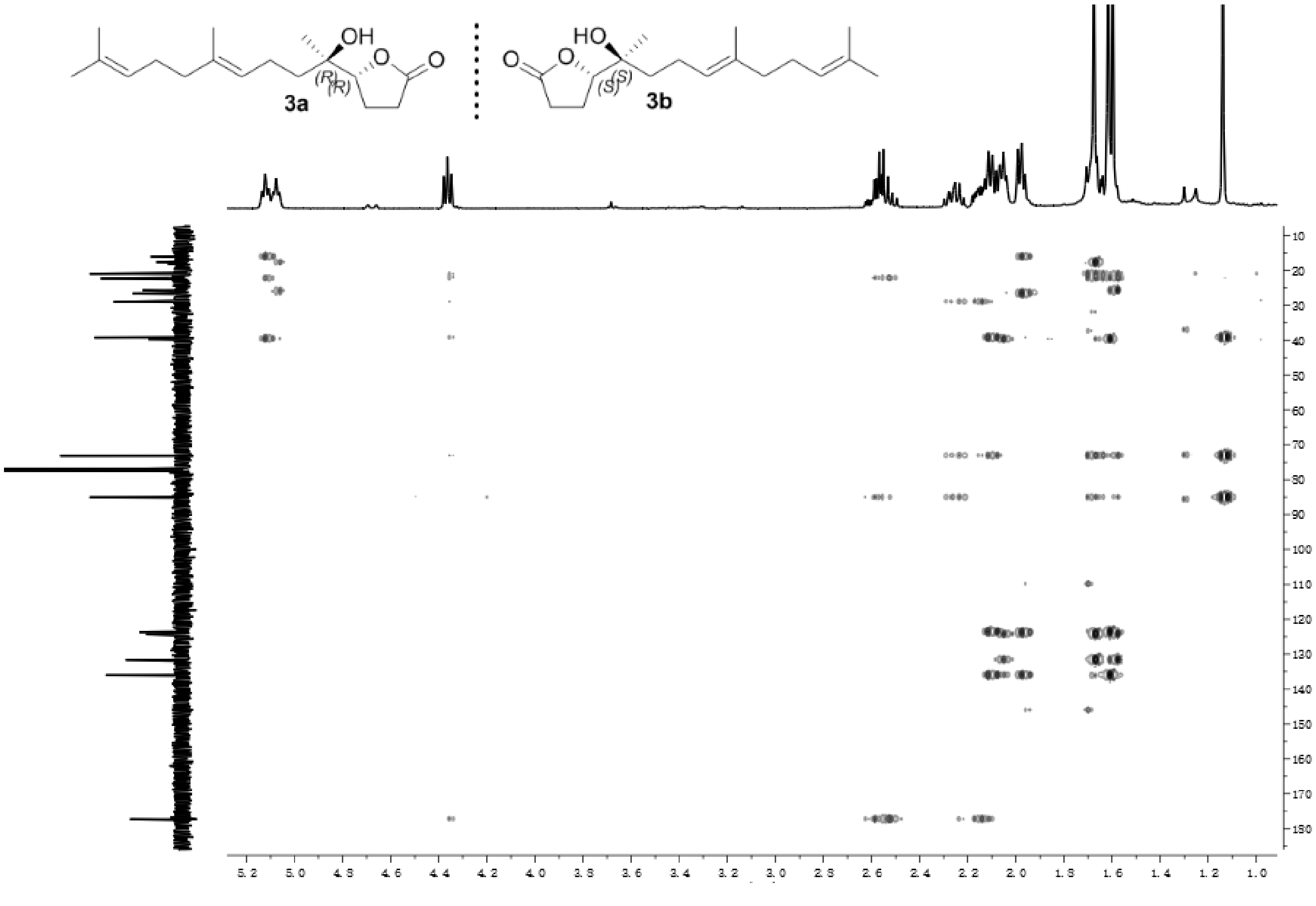

Figure S25. HMBC spectrum of compounds **3a/3b**.

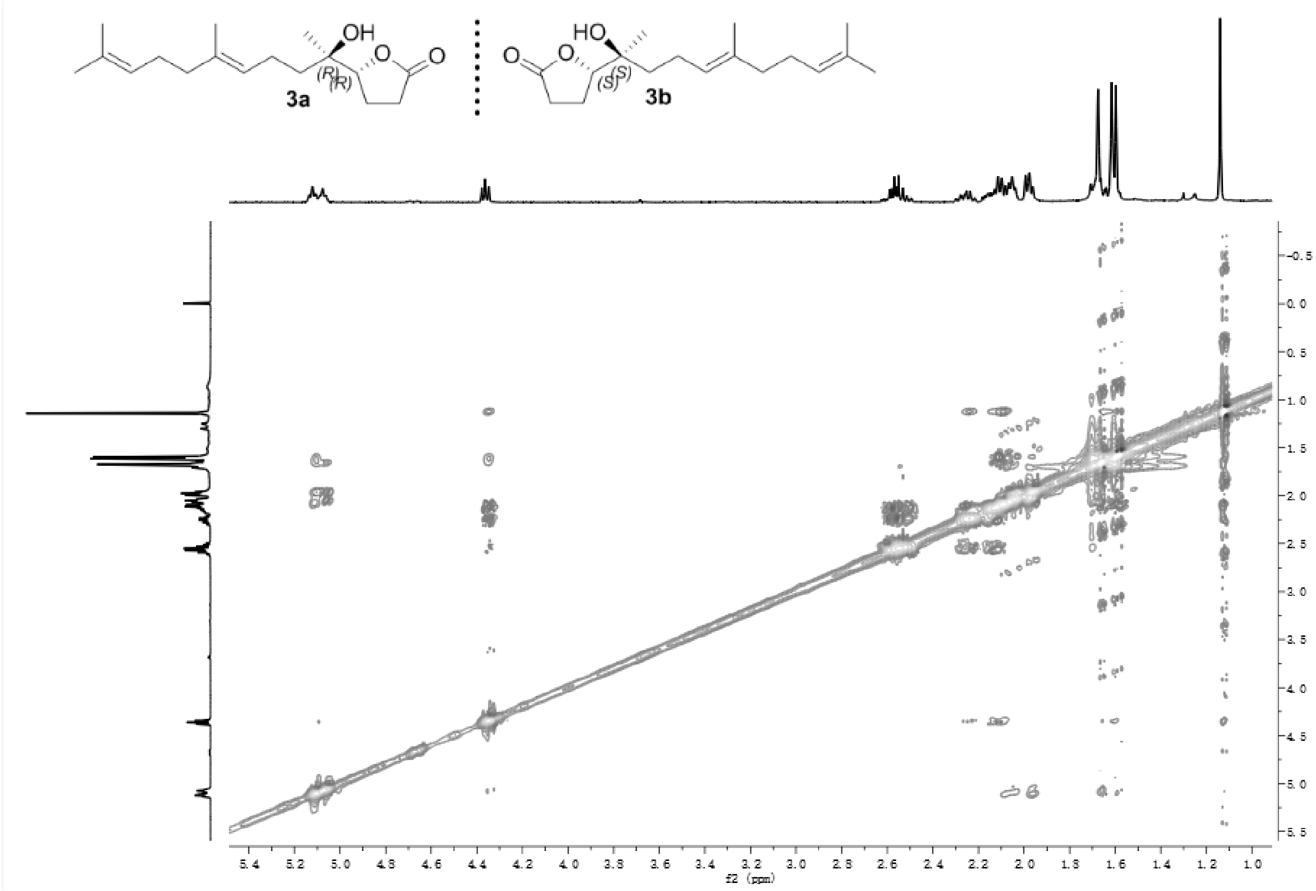

Figure S26. NOESY spectrum of compounds **3a/3b**.

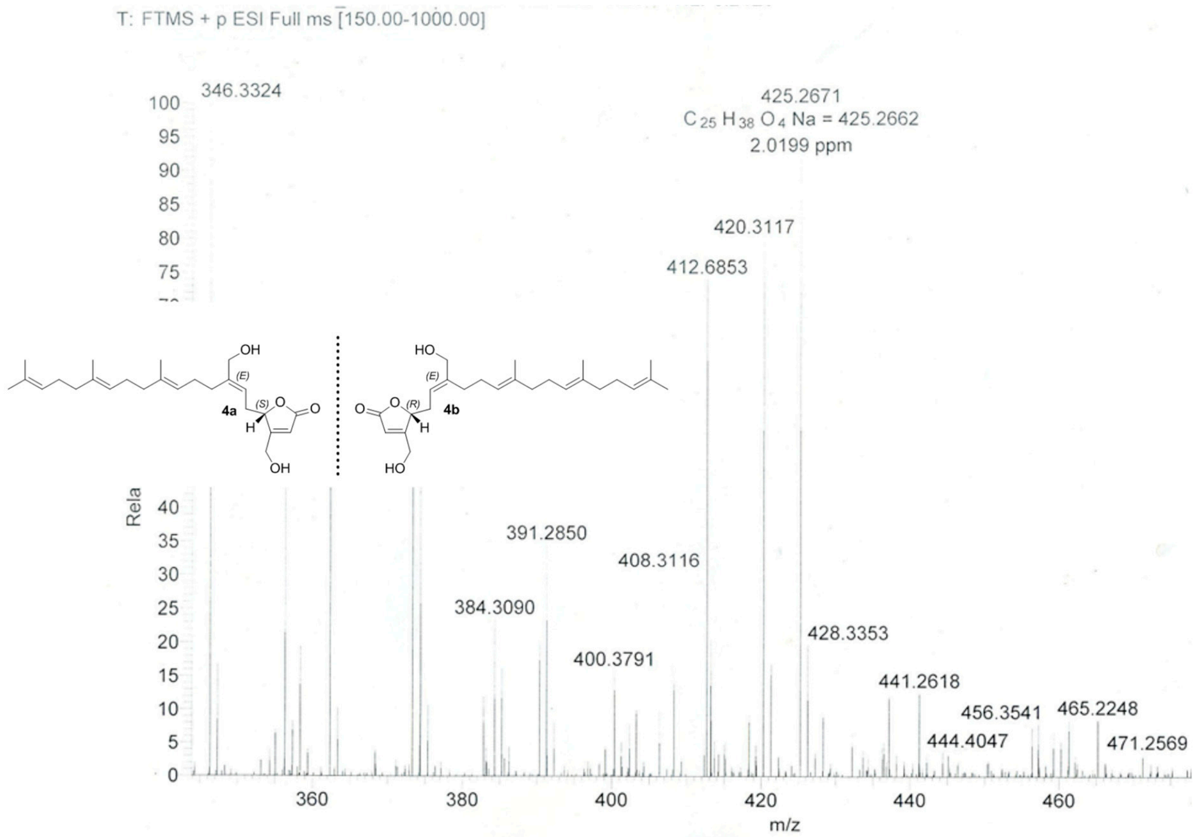

Figure S27. HR-ESI-MS of compounds **4a/4b**.

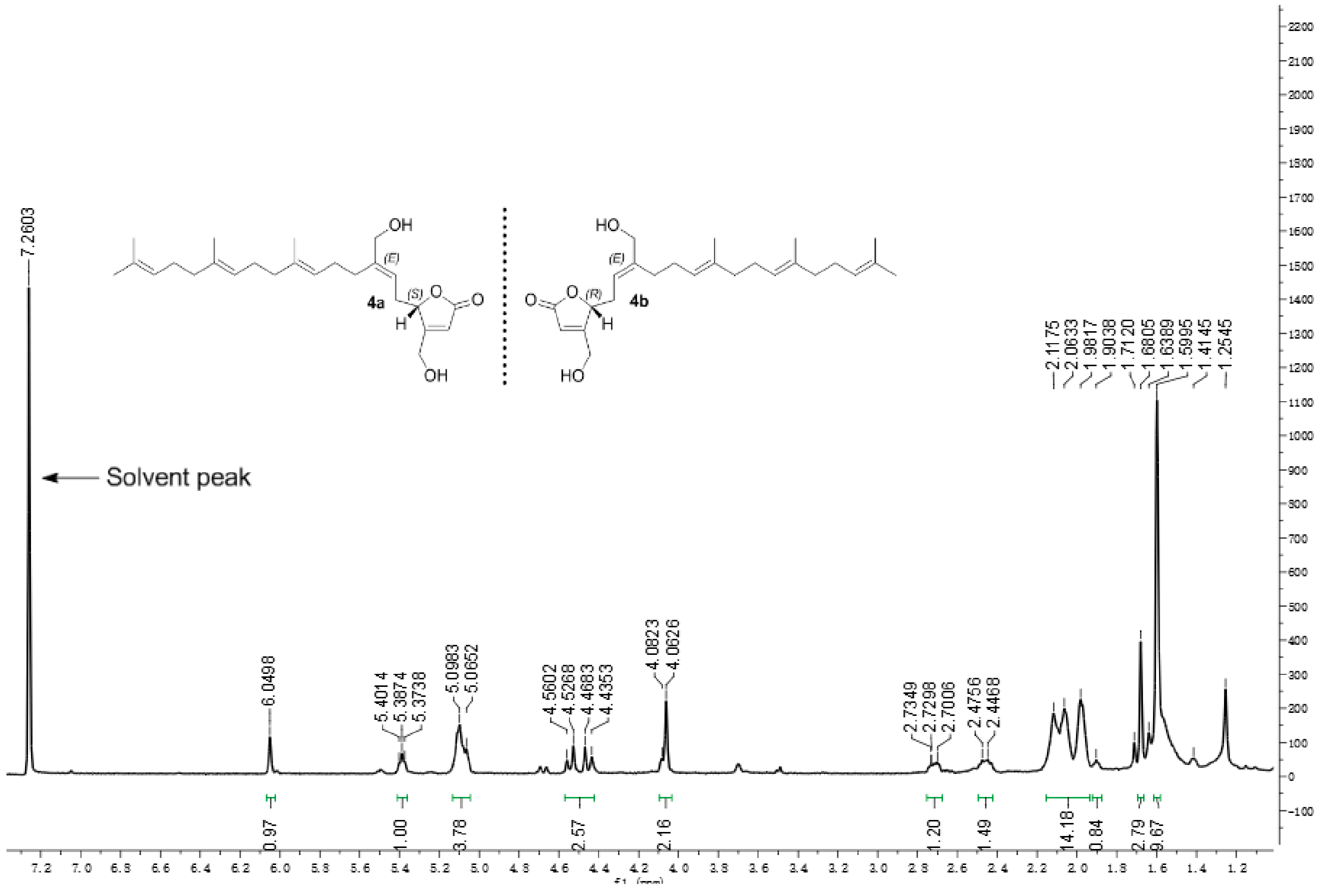

Figure S28. ^{1}H NMR spectrum (500 MHz, $CDCl_3$) of compounds **4a/4b**.

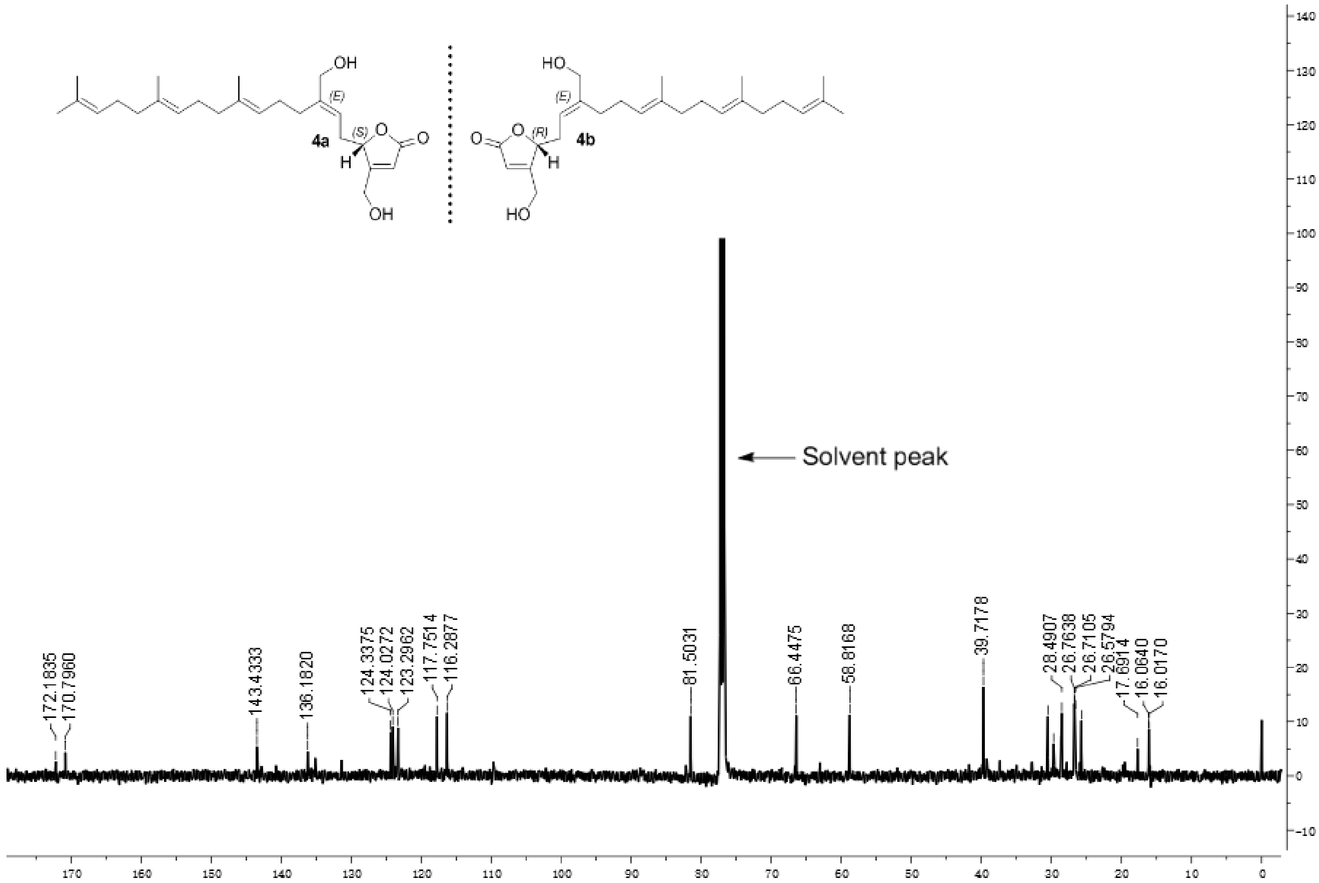

Figure S29. ^{13}C NMR spectrum (125 MHz, $CDCl_3$) of compounds **4a/4b**.

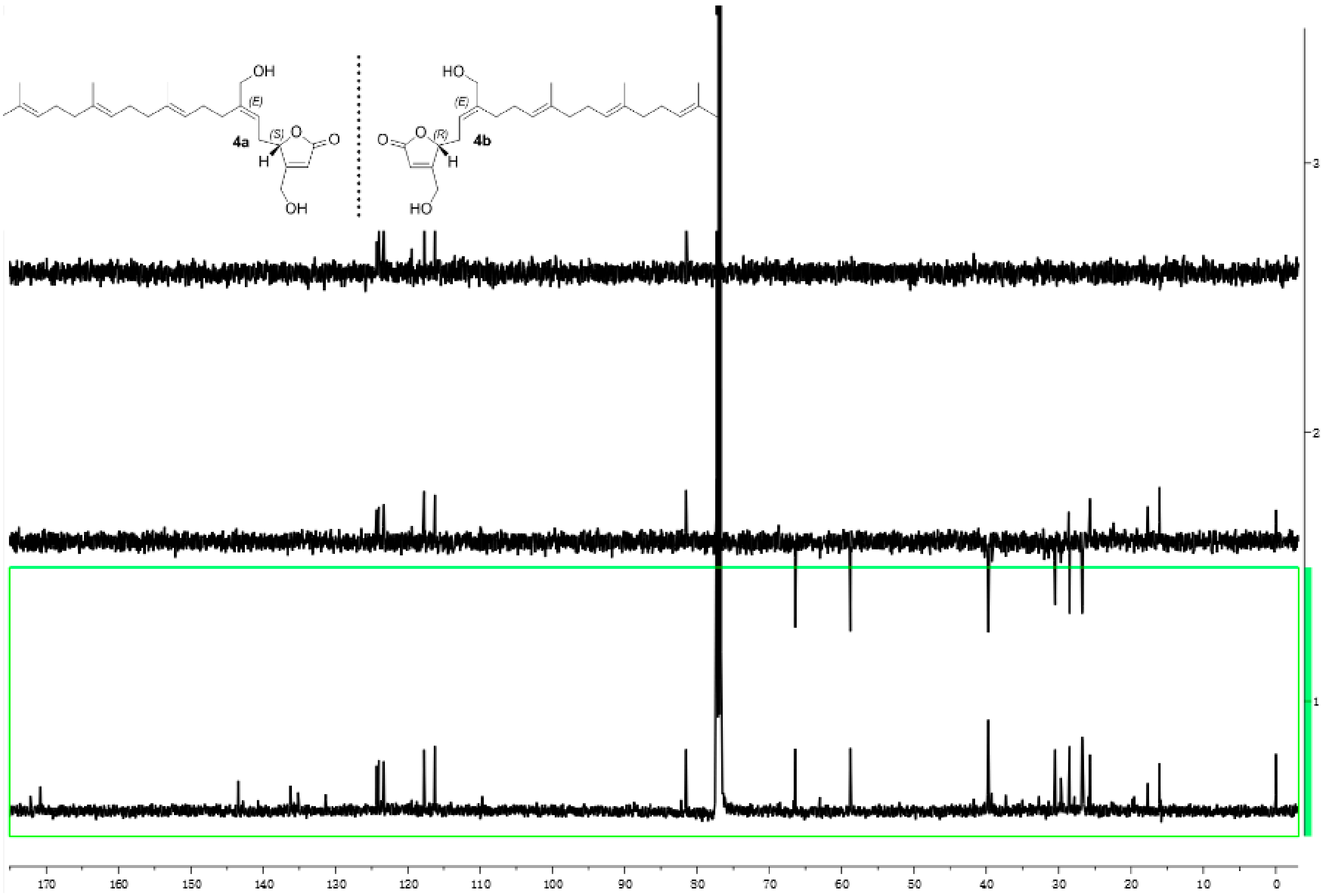

Figure S30. DEPT spectrum (125 MHz, CDCl3) of compounds **4a/4b**.

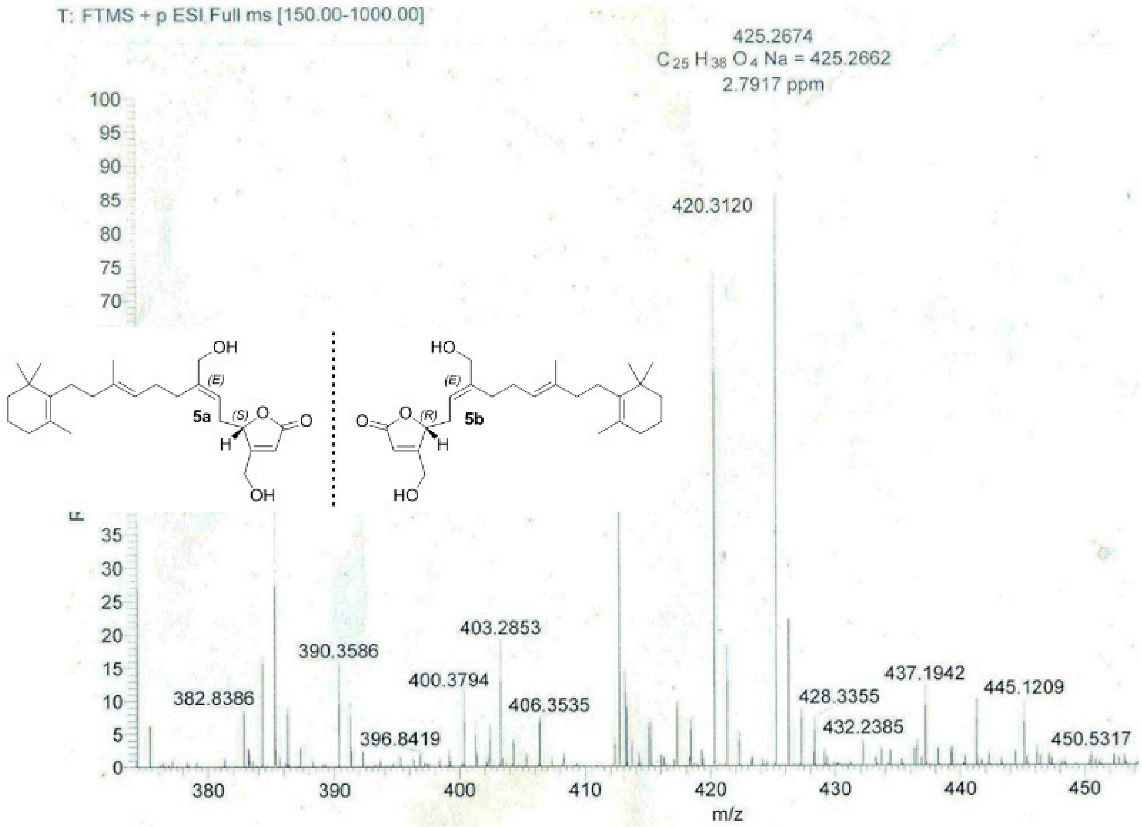

Figure S31. HR-ESI-MS of compounds **5a/5b**.

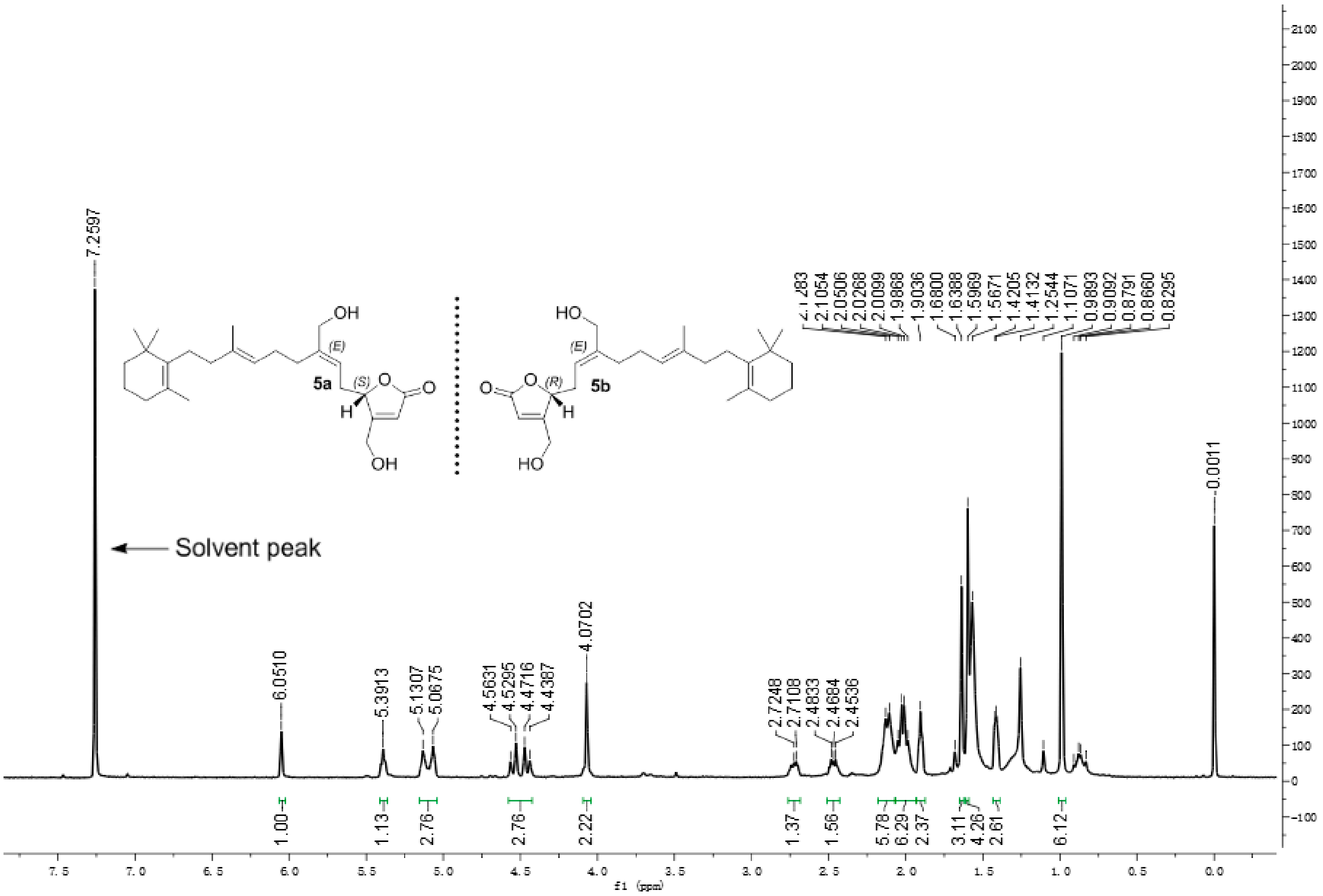

Figure S32. ^{1}H NMR spectrum (500 MHz, $CDCl_3$) of compounds **5a/5b**.

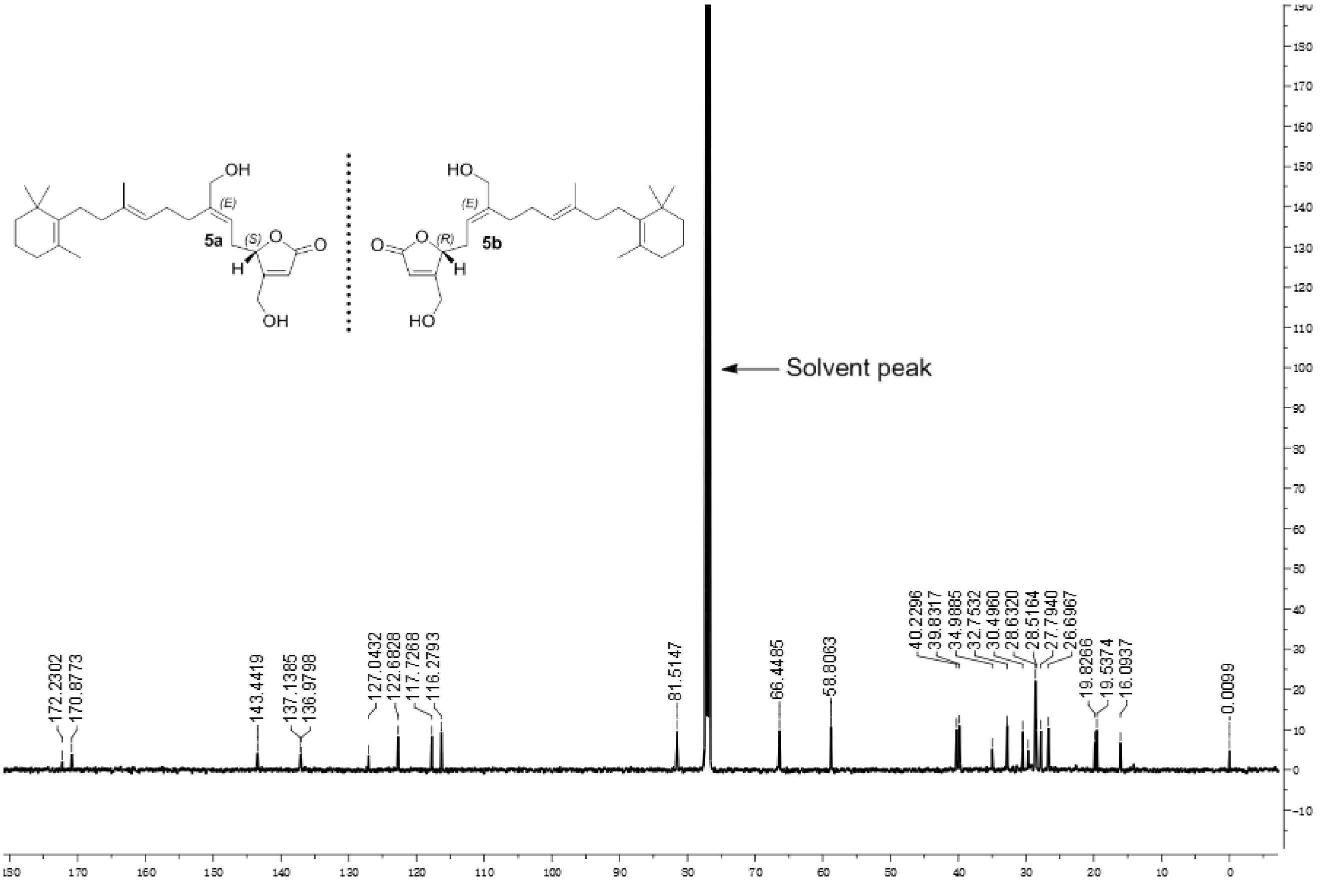

Figure S33. ^{13}C NMR spectrum (125 MHz, $CDCl_3$) of compounds **5a/5b**.

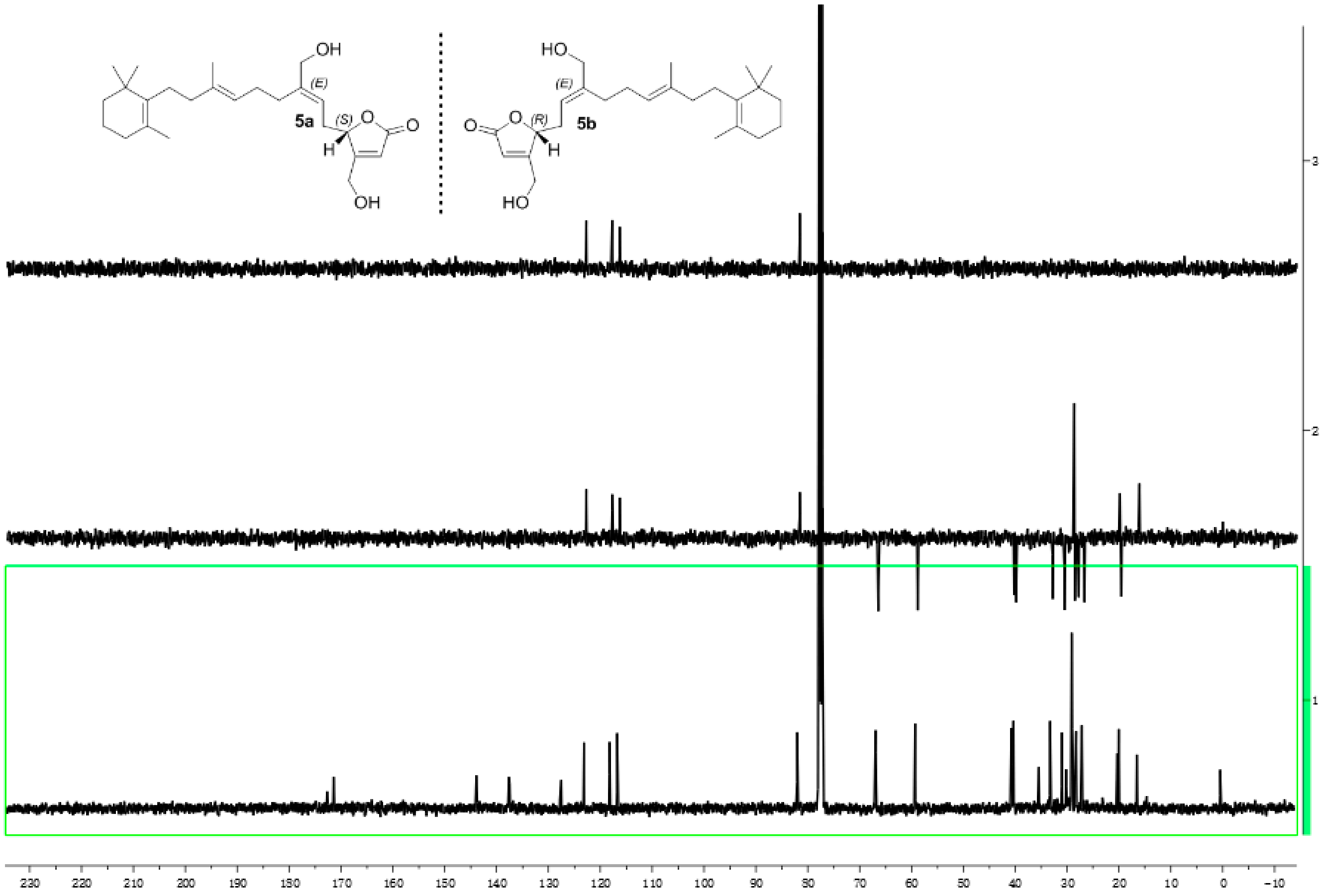

Figure S34. DEPT spectrum (125 MHz, CDCl3) of compounds **5a/5b**.

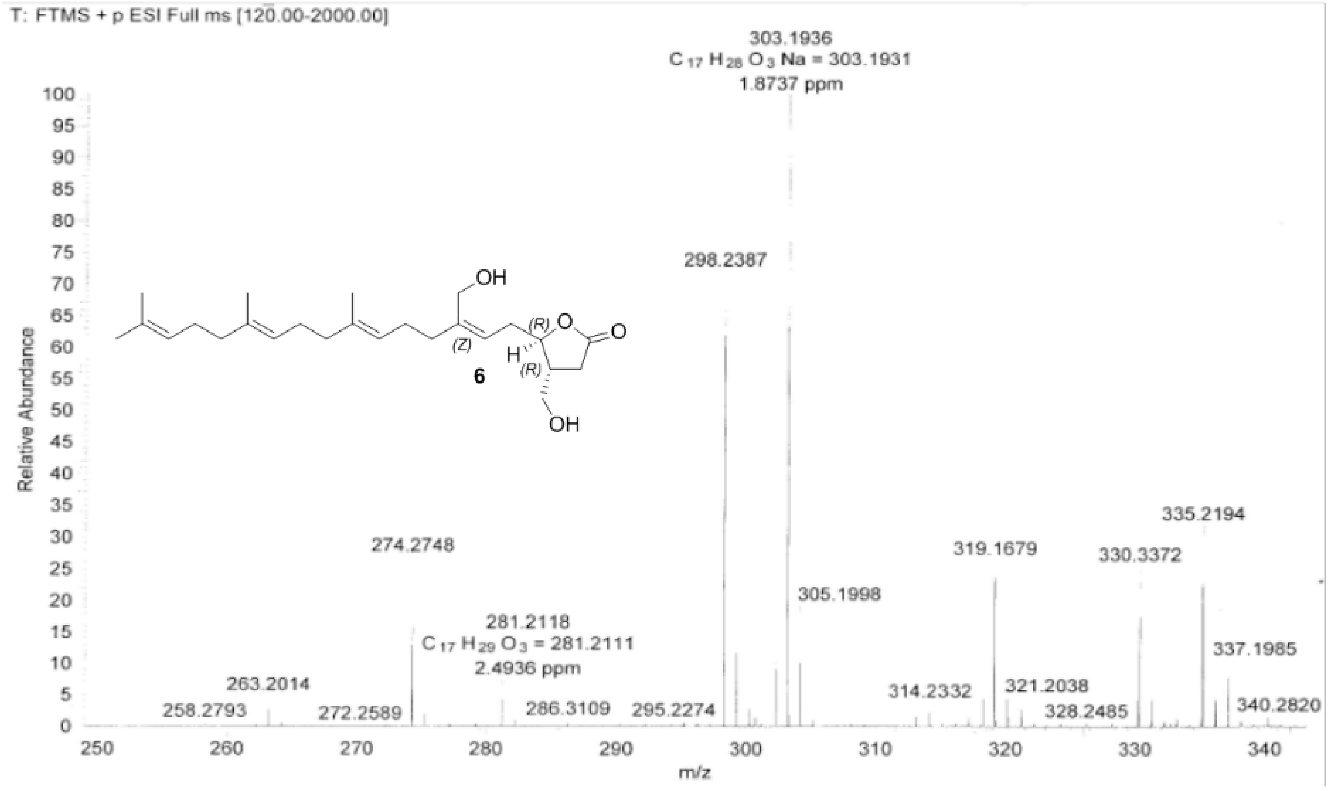

Figure S35. HR-ESI-MS of compound **6**.

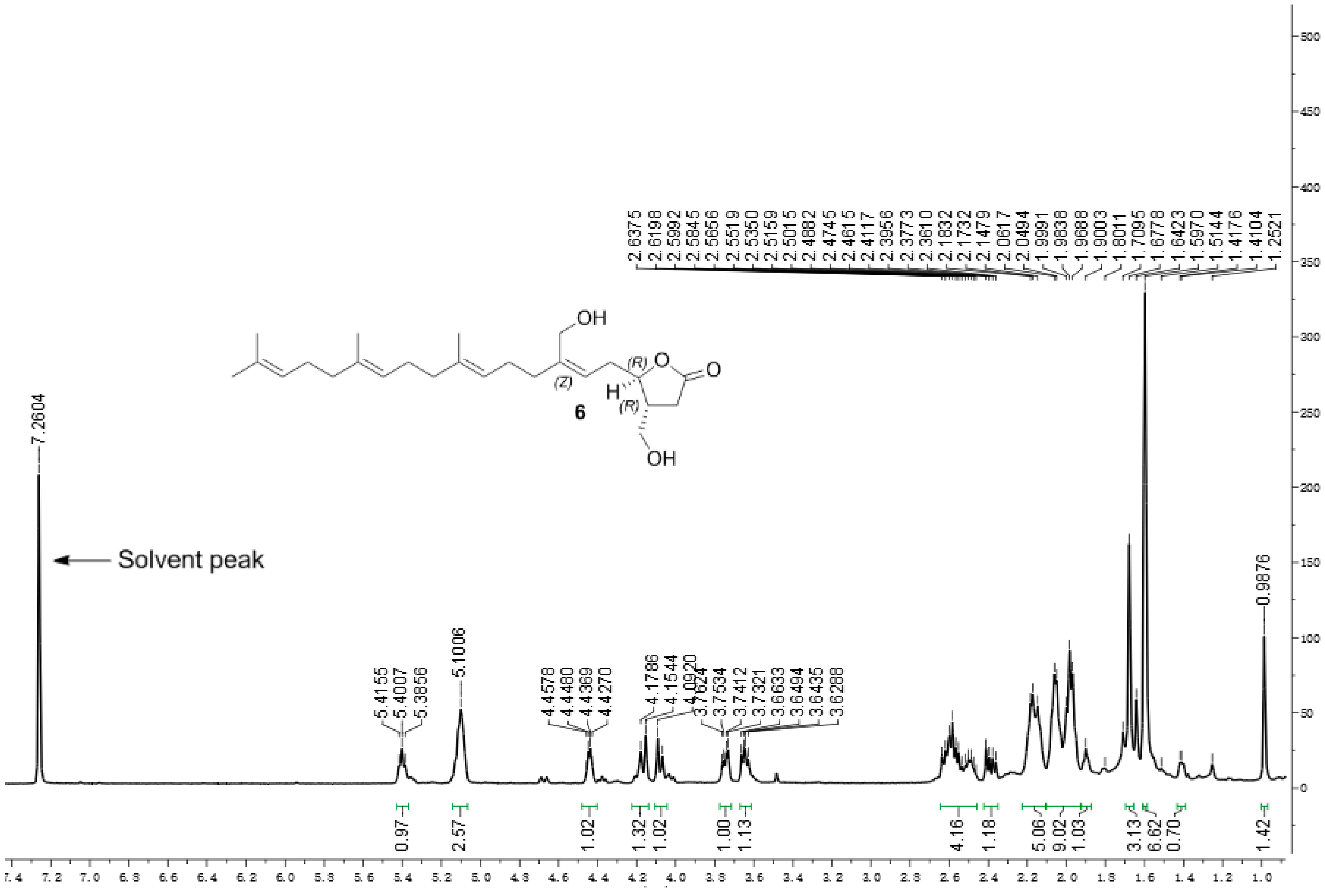

Figure S36. ^{1}H NMR spectrum (500 MHz, $CDCl_3$) of compound **6**.

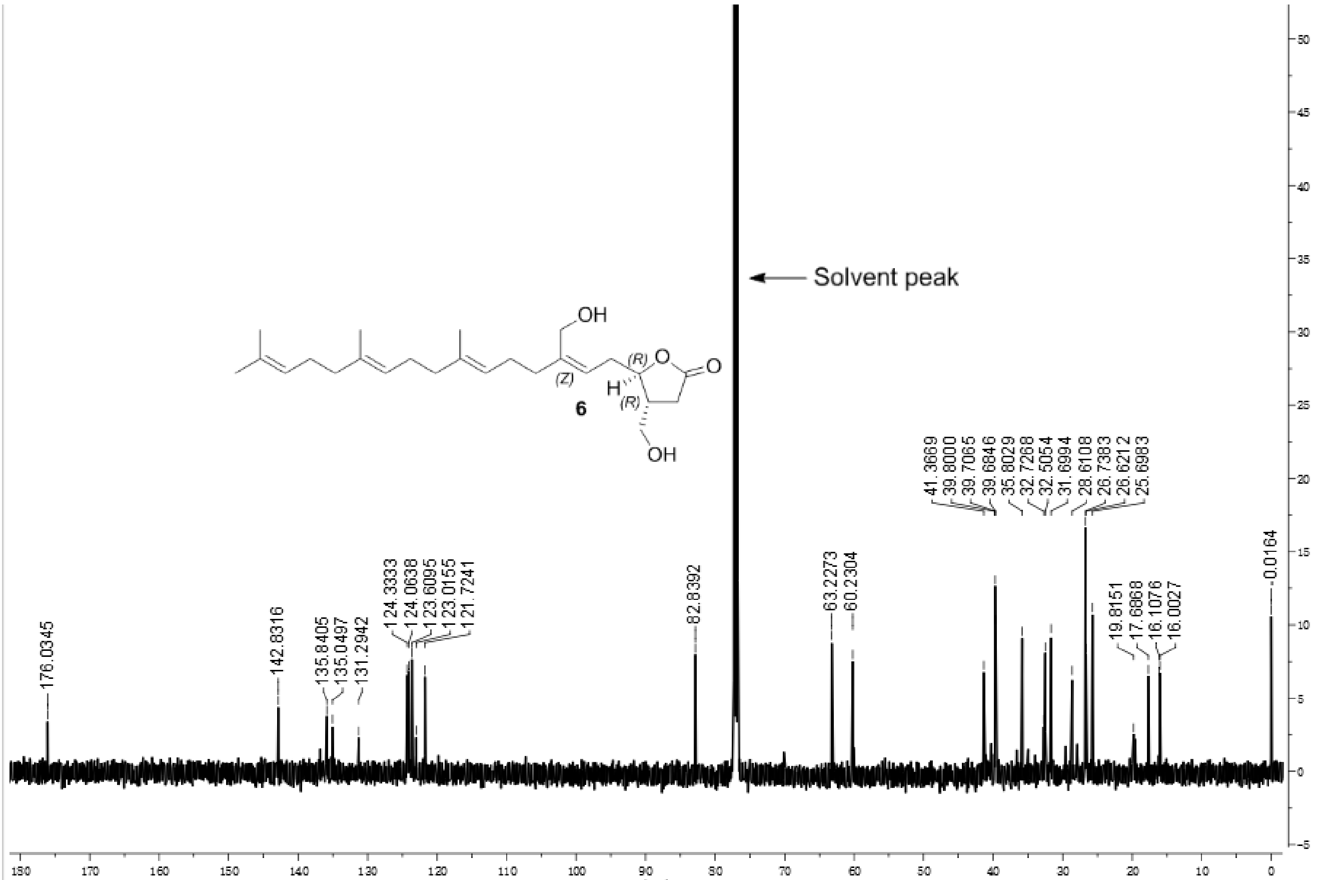

Figure S37. ^{13}C NMR spectrum (125 MHz, $CDCl_3$) of compound **6**.

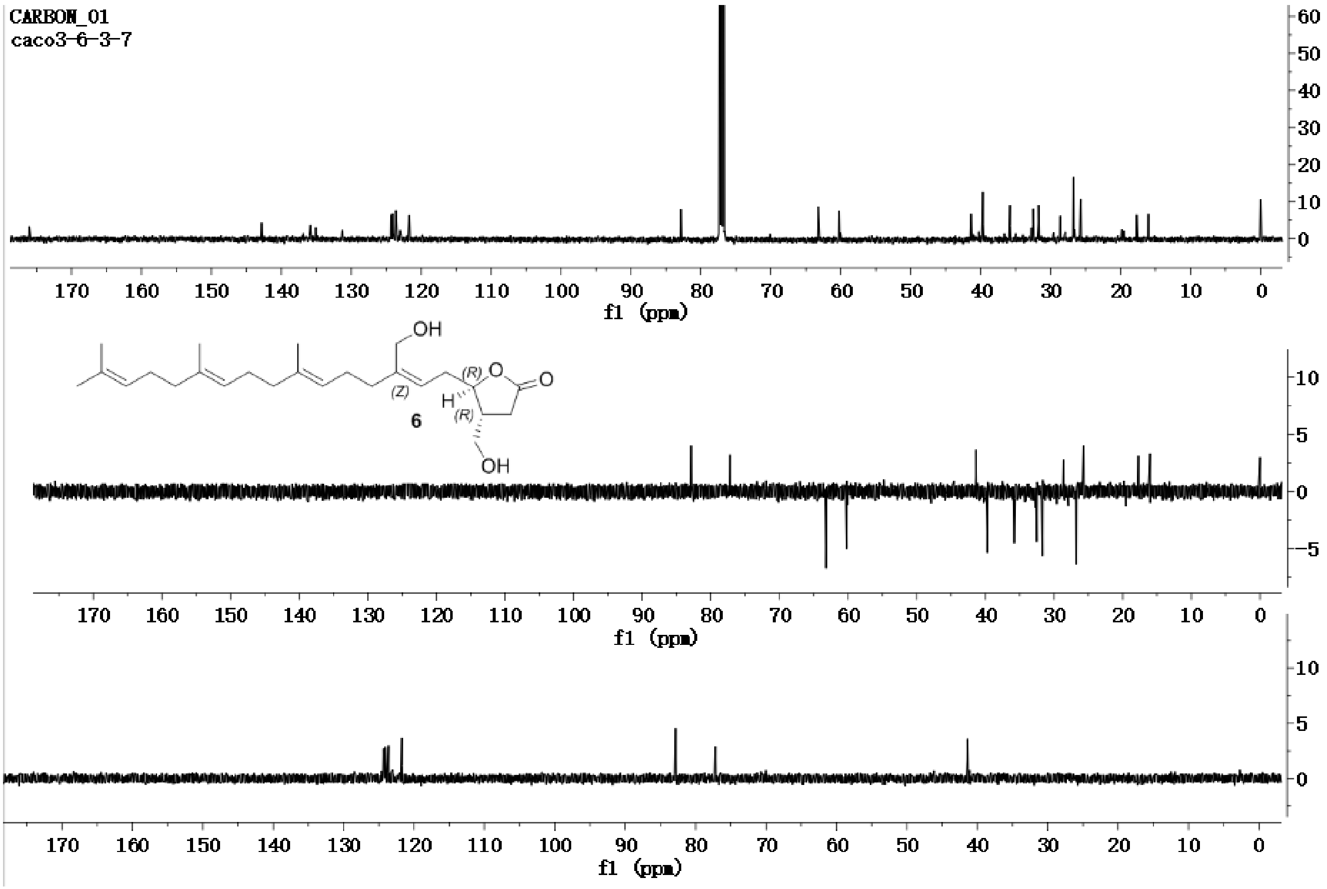

Figure S38. DEPT spectrum (125 MHz, $CDCl_3$) of compound **6**.

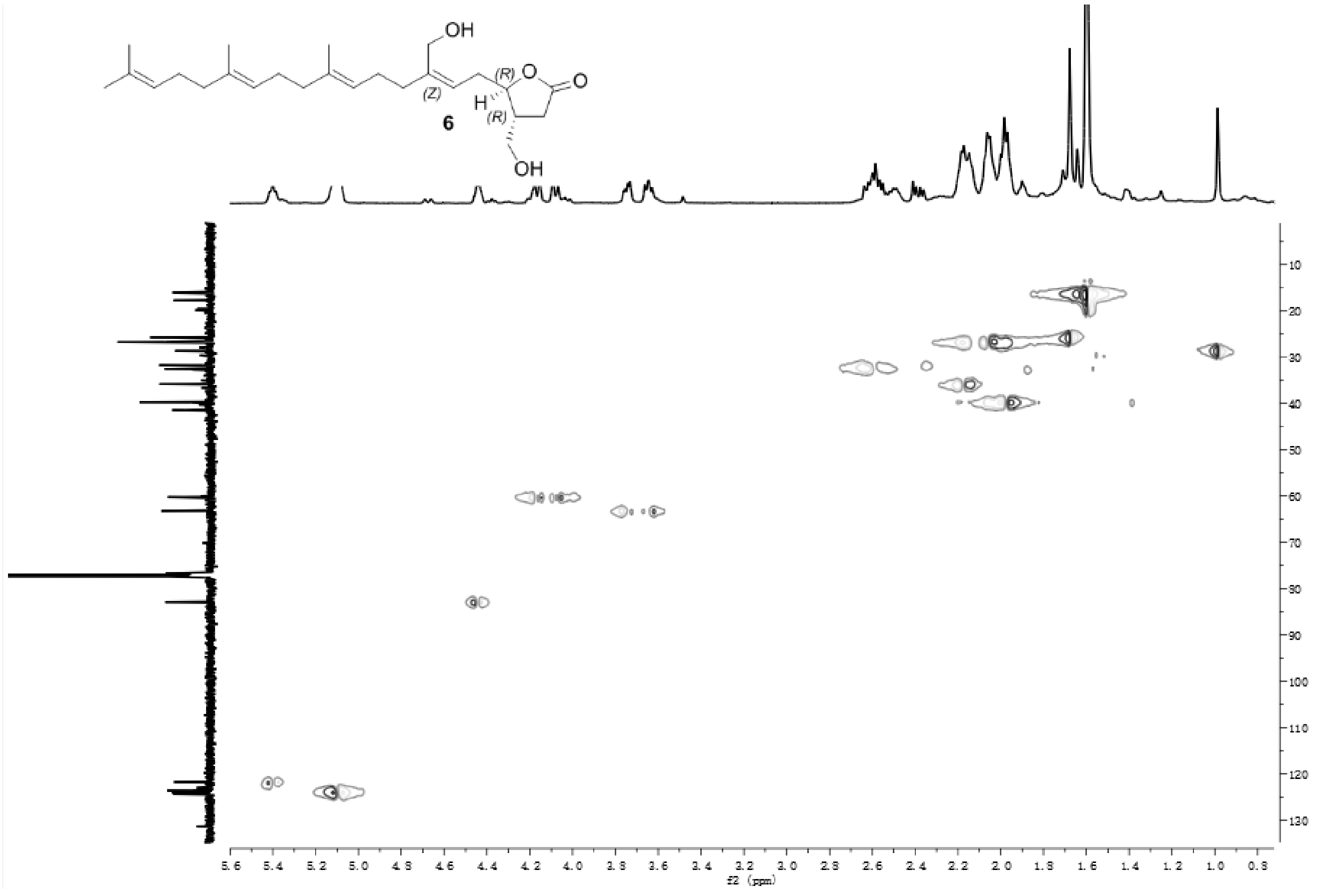

Figure S39. HSQC spectrum of compound **6**.

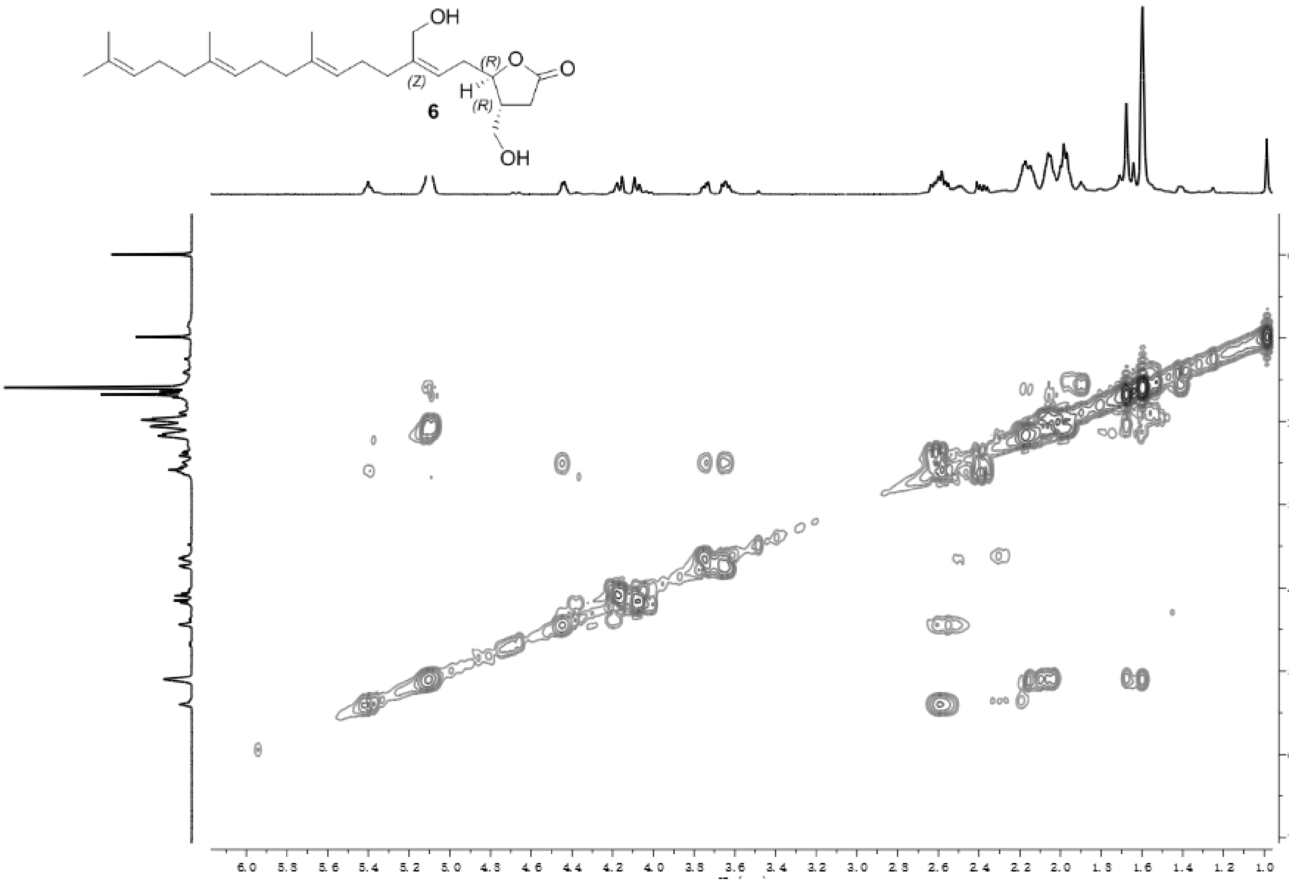

Figure S40. ^{1}H-^{1}H COSY spectrum of compound **6**.

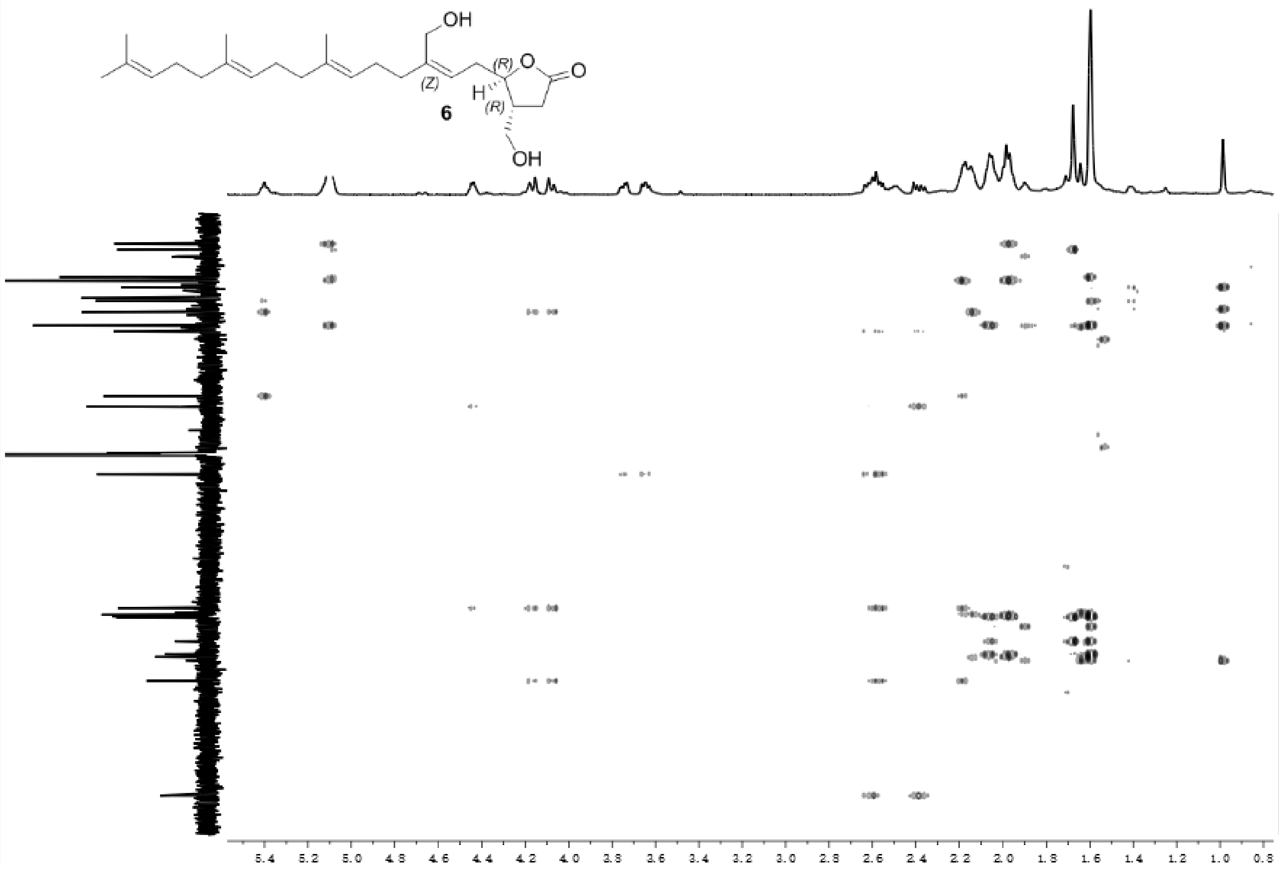

Figure S41. HMBC spectrum of compound **6**.

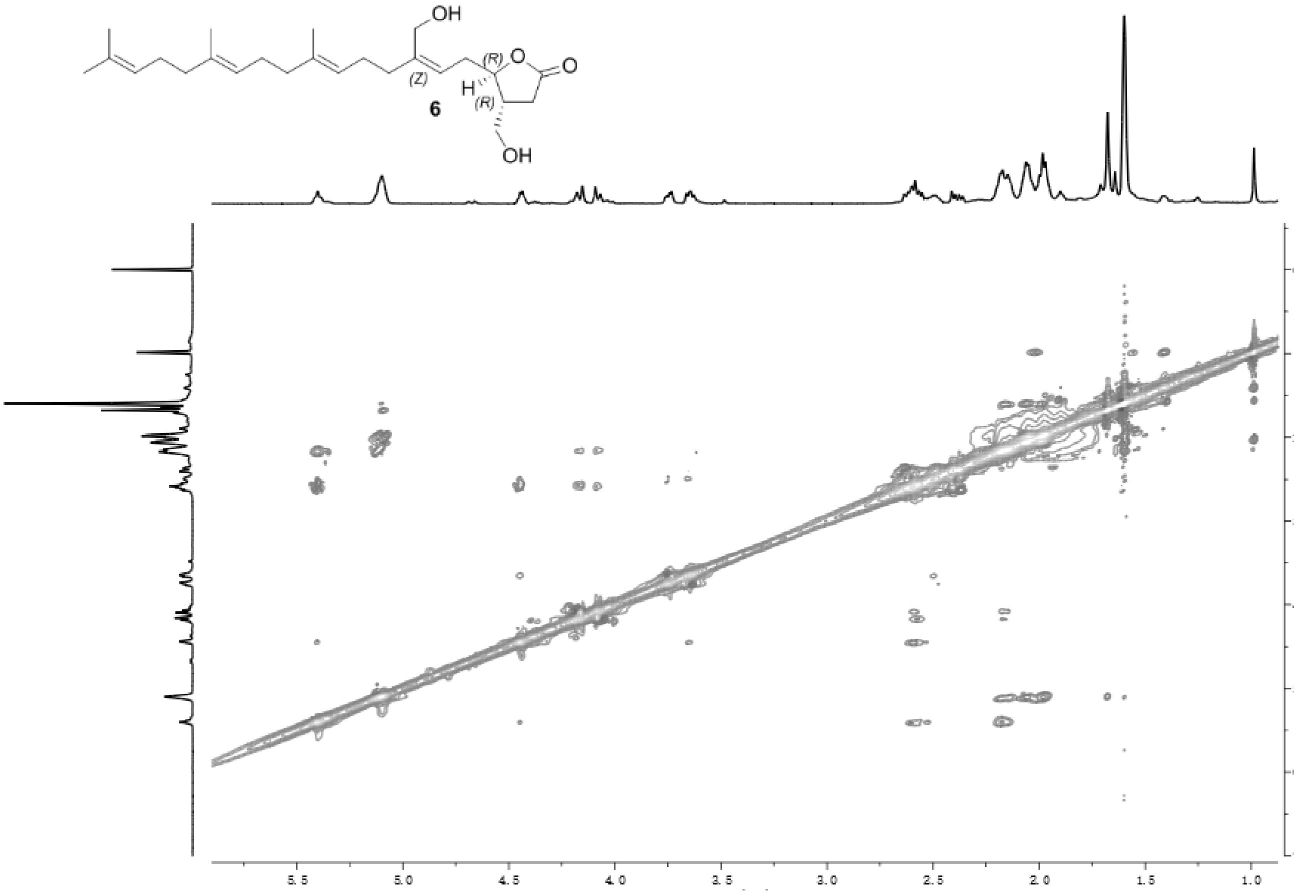

Figure S42. NOESY spectrum of compound **6**.

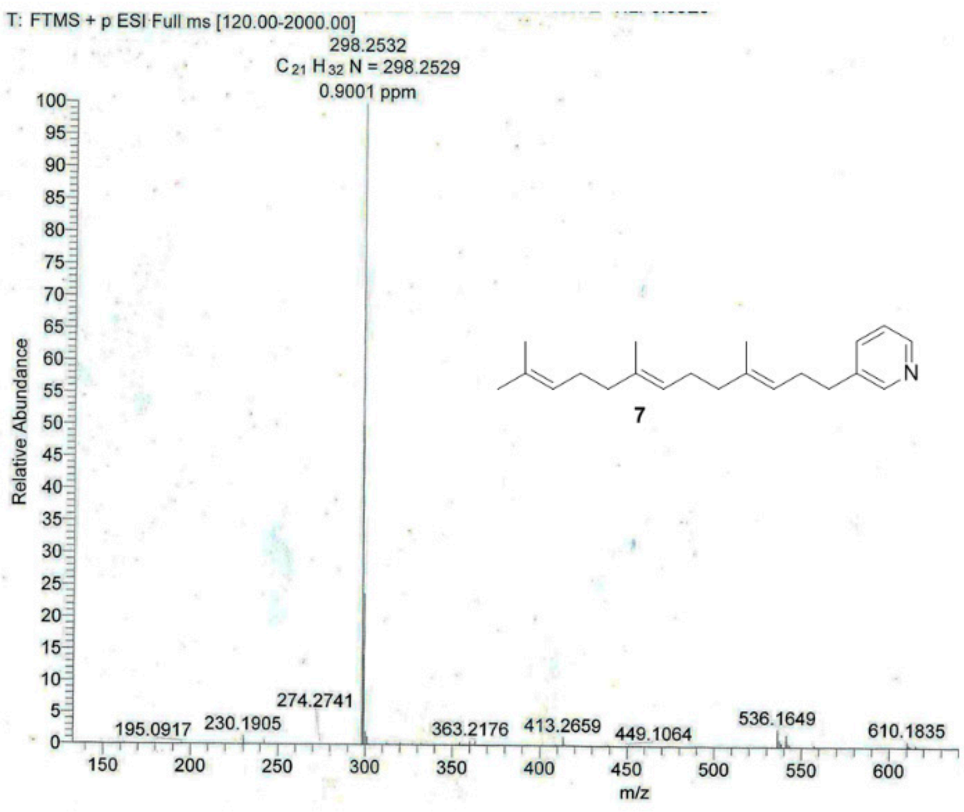

Figure S43. -of compound **7**.

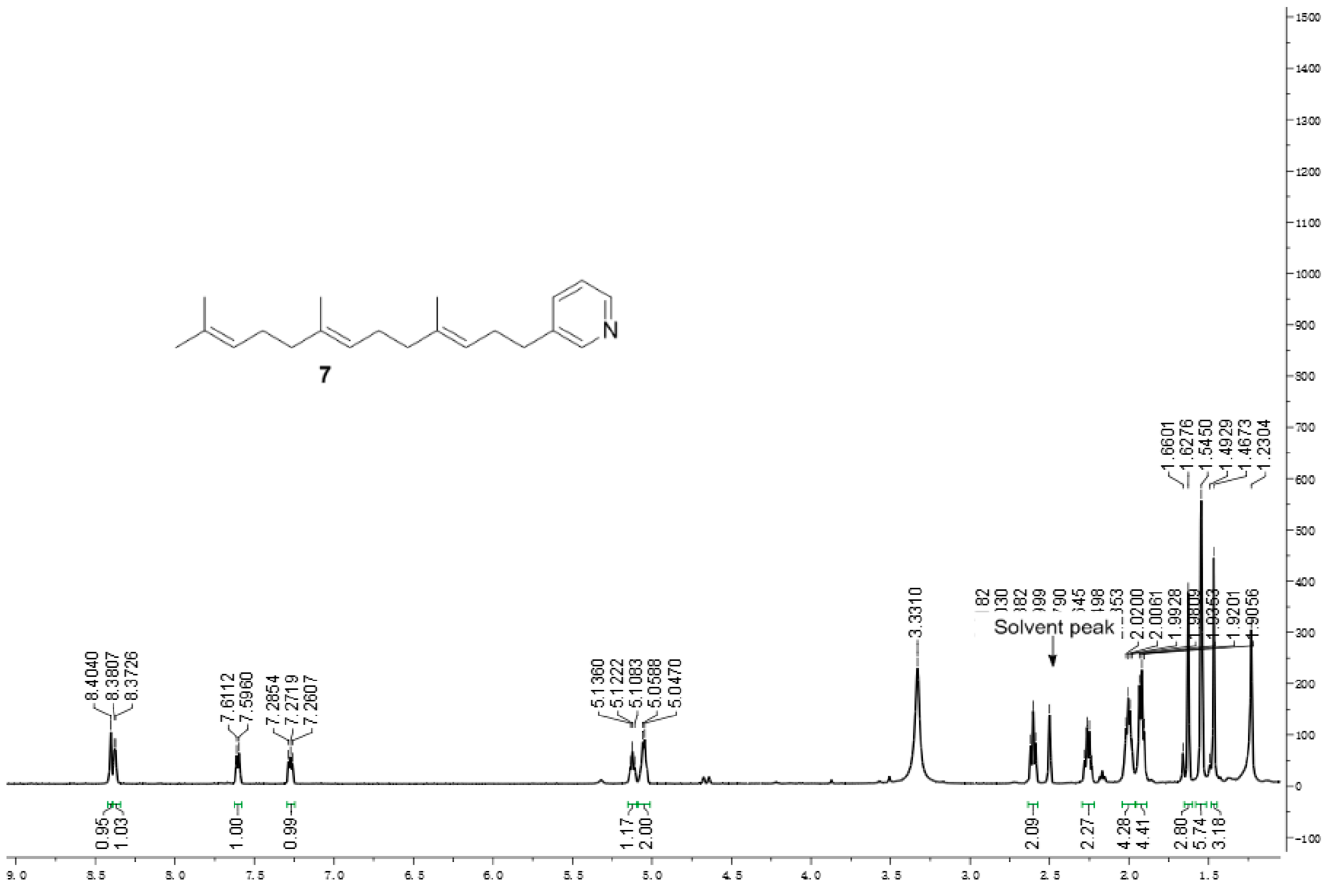

Figure S44. ^{1}H NMR spectrum (500 MHz, DMSO-d_6) of compound **7**.

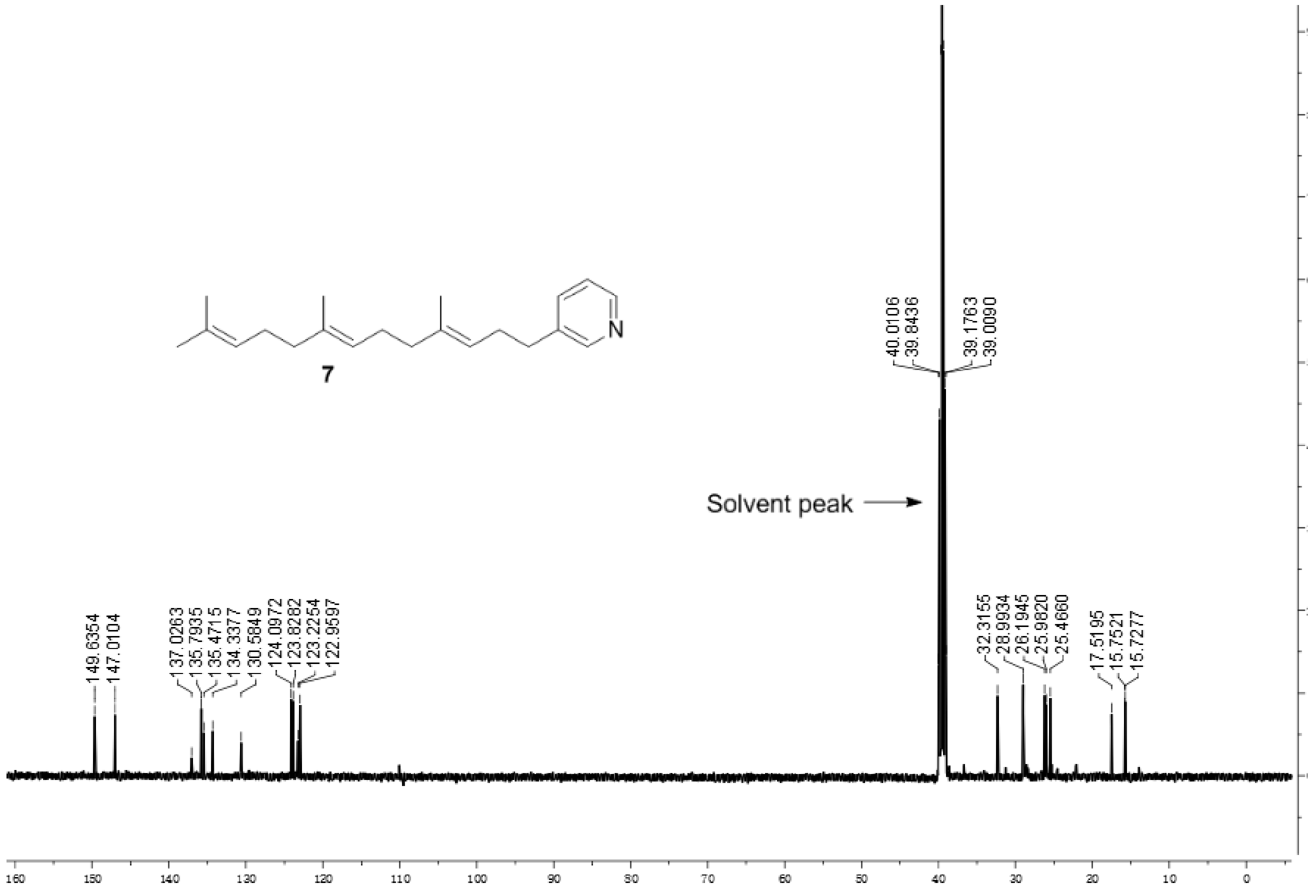

Figure S45. ^{13}C NMR spectrum (125 MHz, DMSO-d_6) of compound **7**.

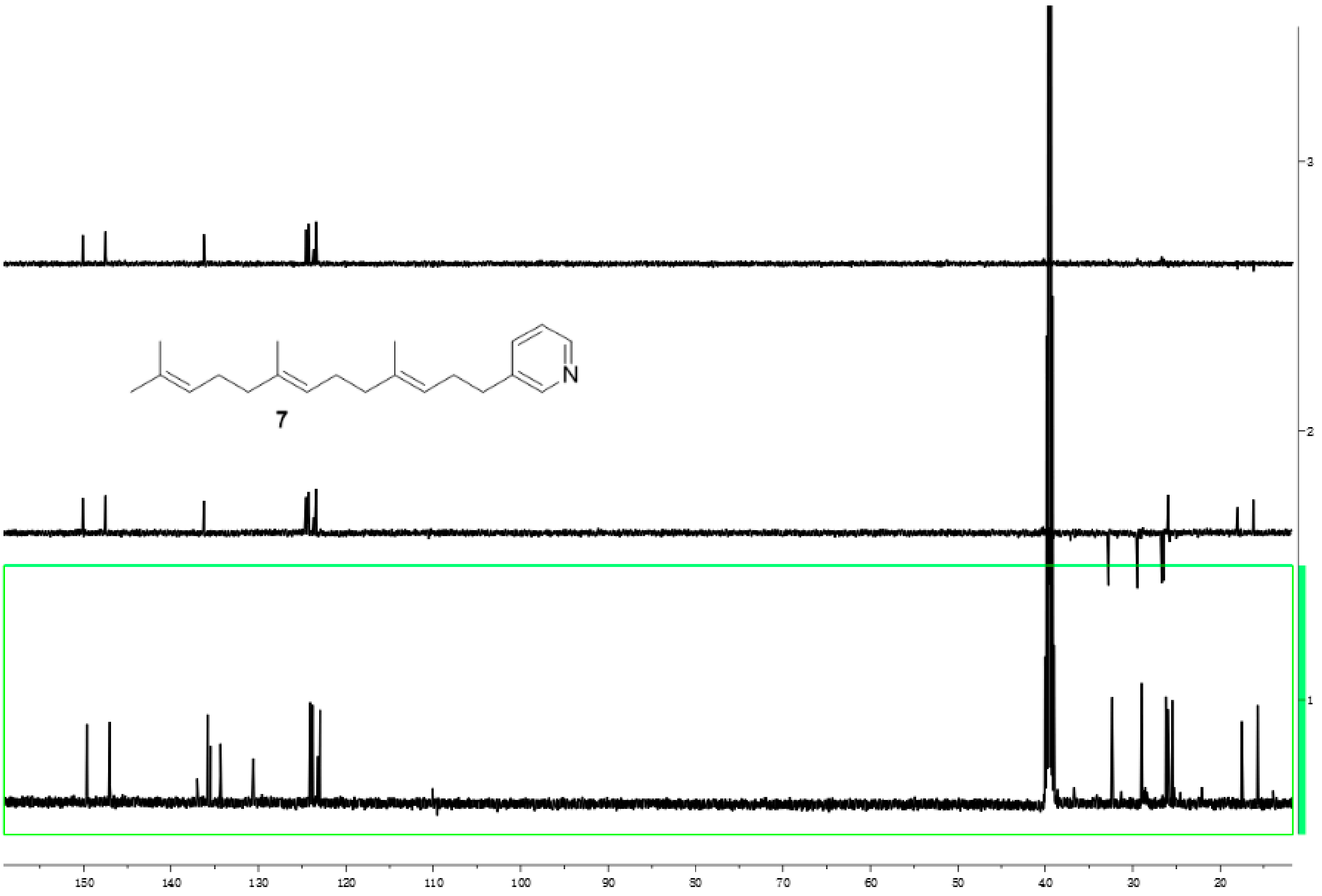

Figure S46. DEPT spectrum (125 MHz, DMSO-d_6) of compound **7**.

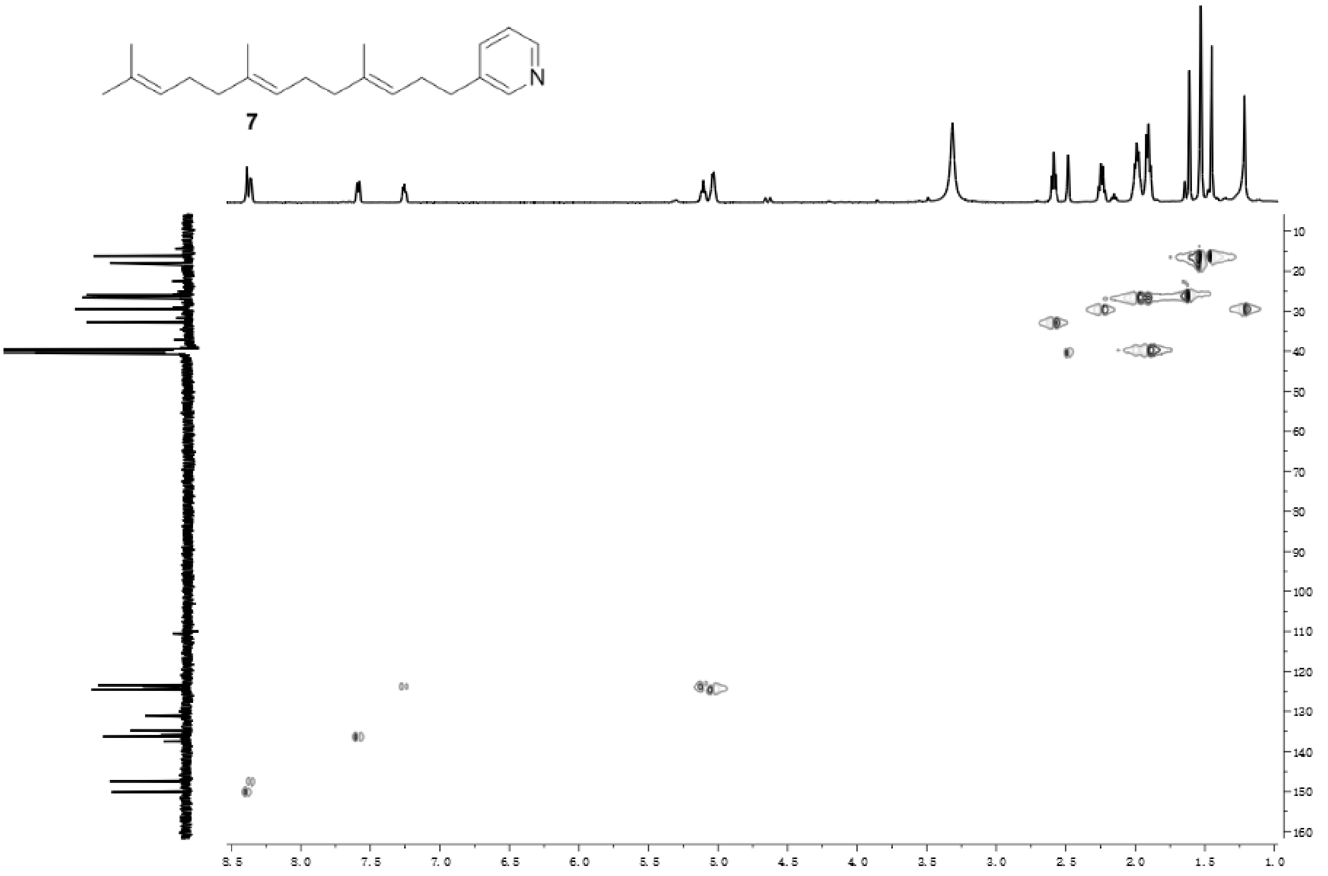

Figure S47. HSQC spectrum of compound **7**.

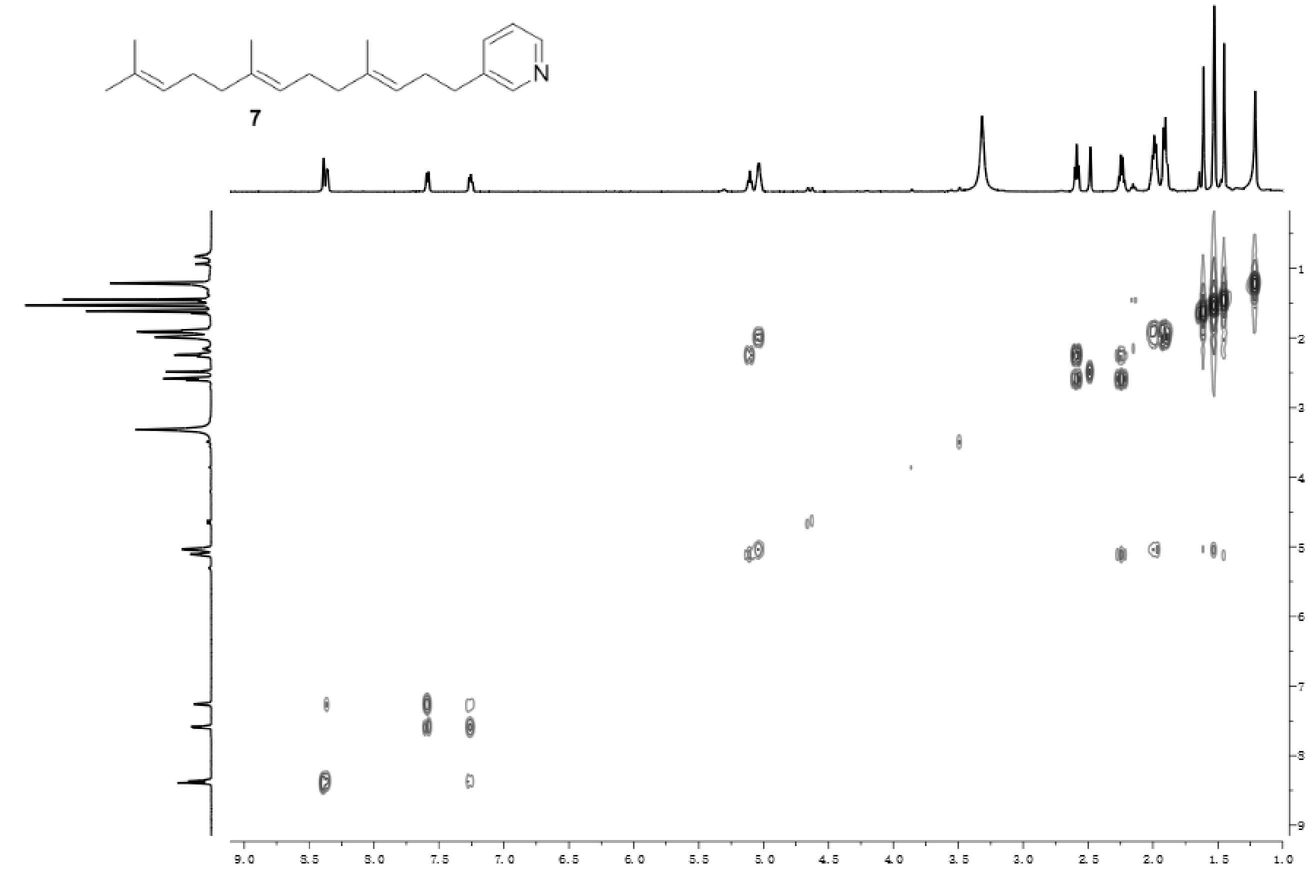

Figure S48. ^{1}H-^{1}H COSY spectrum of compound **7**.

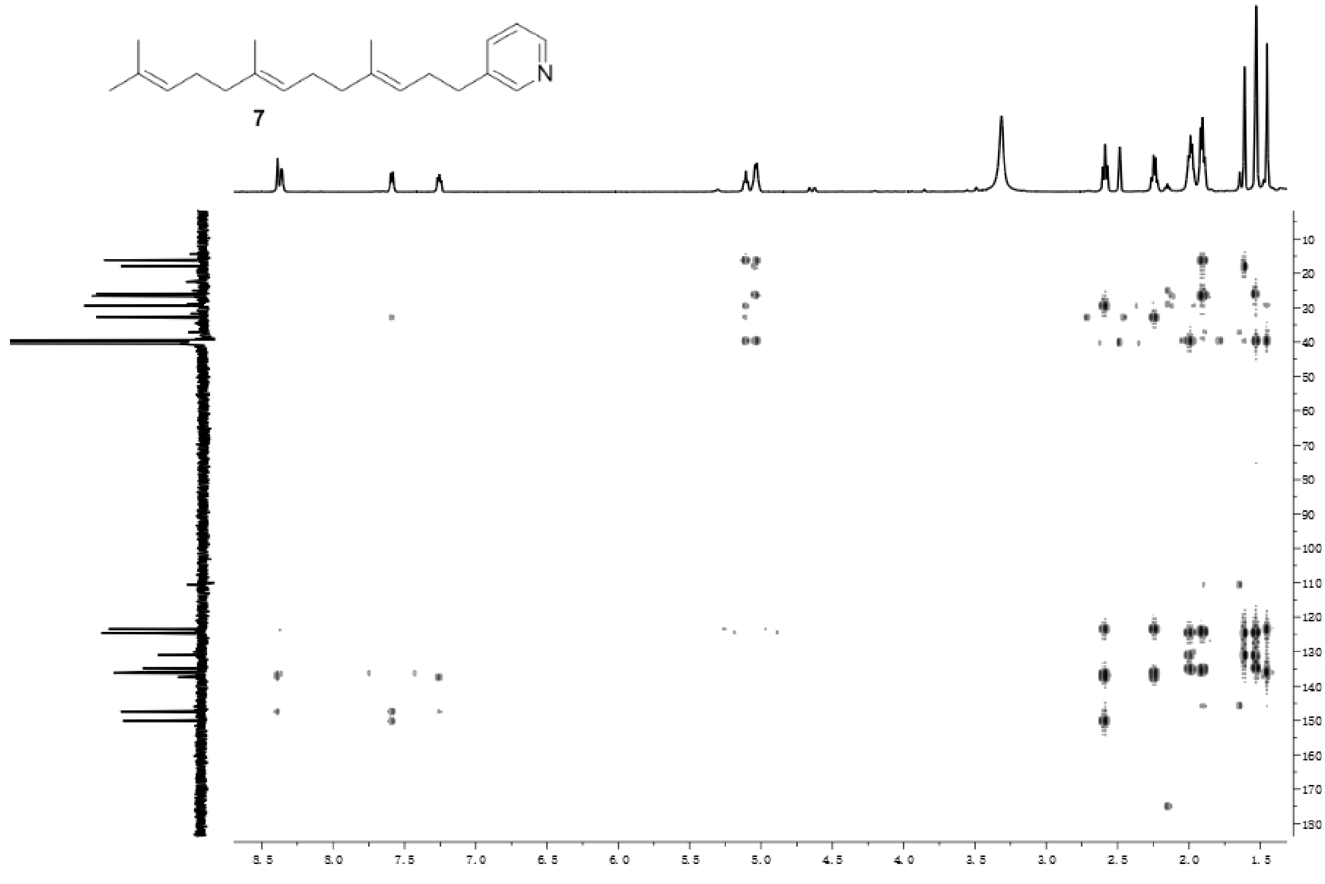

Figure S49. HMBC spectrum of compound **7**.
